# Aqueous Incubation Reveals Transformation of Rosmarinic Acid in Medicinally Important Nepetoideae Species and Leads to Novel Bioactive Compounds

**DOI:** 10.3390/pharmaceutics18081043

**Published:** 2026-08-21

**Authors:** Adila Nazli, Bernadett Szögi-Tatár, Szilvia Bősze, Gergő Tóth, Katalin Solymosi, Szabolcs Béni, Imre Boldizsár

**Affiliations:** 1 Department of Pharmacognosy, Semmelweis University, 1085 Budapest, Hungary; nazli.adila@phd.semmelweis.hu (A.N.); szogitatarbernadett@gmail.com (B.S.-T.); 2 HUN-REN-ELTE Research Group of Peptide Chemistry, Hungarian Research Network, ELTE Eötvös Loránd University, Pázmány Péter sétány 1/A, 1117 Budapest, Hungary; szilvia.bosze@ttk.elte.hu; 3 Department of Genetics, Cell- and Immunobiology, Semmelweis University, Nagyvárad tér 4, 1089 Budapest, Hungary; 4 Department of Pharmaceutical Chemistry, Semmelweis University, Hőgyes Endre u. 9, 1092 Budapest, Hungary; toth.gergo@semmelweis.hu; 5 Center for Pharmacology and Drug Research & Development, Semmelweis University, 1085 Budapest, Hungary; 6 Department of Plant Anatomy, Institute of Biology, Eötvös Loránd University, Pázmány Péter sétány 1/C, 1117 Budapest, Hungary; katalin.solymosi@ttk.elte.hu; 7 Integrative Health and Environmental Analysis Research Laboratory, Department of Analytical Chemistry, Institute of Chemistry, Eötvös Loránd University, 1117 Budapest, Hungary

**Keywords:** antioxidant, cytostatic, *Mentha*, Nepetoideae, nepetoidin, rosmarinic acid, *Salvia*, salvianolic acid

## Abstract

**Background:** Rosmarinic acid (RA) is a major phenylpropanoid constituent of many medicinal plants belonging to the Nepetoideae subfamily of the Lamiaceae family and contributes significantly to their biological activities. Traditionally, RA-containing extracts have been prepared using organic solvents or hot-water extraction. **Methods:** In addition to conventional extraction techniques, the phenylpropanoid composition of medicinally important tissues from 13 Nepetoideae species was investigated following incubation in an aqueous medium at room temperature. Phenylpropanoids in the differently prepared extracts were identified by extensive HPLC-UV-HR-MS/MS and NMR analyses. The antioxidant and cytostatic activities of isolated phenylpropanoids were evaluated using DPPH and Alamar Blue assays, respectively. **Results:** In tissues of all investigated species, RA underwent rapid enzyme-catalyzed conversion during aqueous incubation through oxidative decarboxylation, accompanied by the transient accumulation of nepetoidins A and B. The RA-related phenylpropanoid salvianolic acid K was identified in the roots of three *Salvia* species, while a previously undescribed isomer, designated salvianolic acid O, was discovered in the roots of *Salvia verticillata*. Both compounds underwent enzymatic transformations analogous to those of RA in aqueous medium, yielding two previously undescribed phenylpropanoids, salvianolic acids P and Q. Optimized aqueous incubation conditions enabled the high-yield accumulation of these conversion products. Together with their parent compounds, seven phenylpropanoids were isolated from selected plant tissues and were found to exhibit structure-dependent antioxidant and metabolic inhibitory activities in vitro. **Conclusions:** Aqueous incubation of Nepetoideae plant tissues markedly alters their phytochemical composition and provides access to previously unavailable bioactive phenylpropanoids.

## 1. Introduction

**Figure 1:**
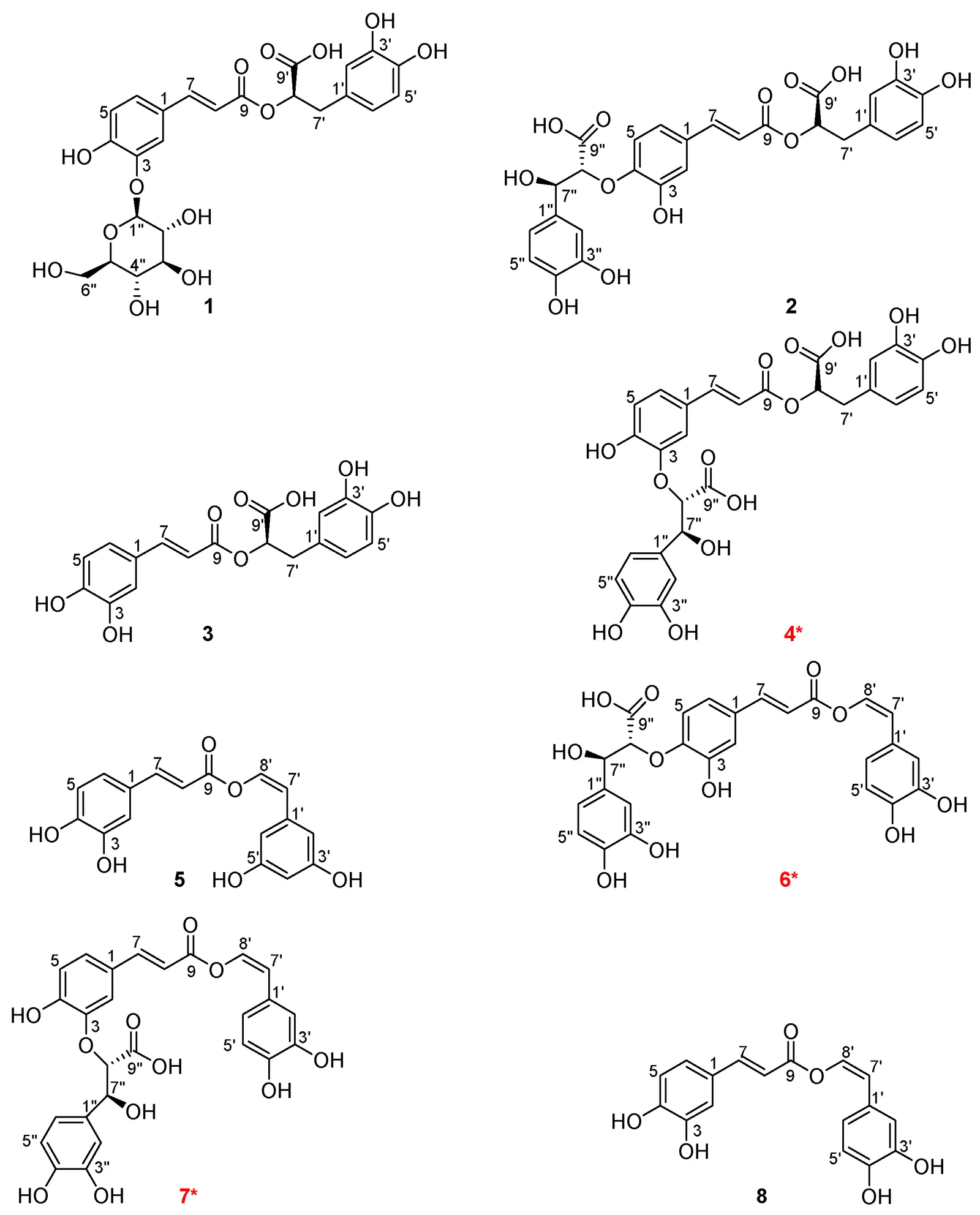
Structures of rosmarinic acid-3-O-β-D-glucoside (**1**), salvianolic acid K (**2**), rosmarinic acid (**3**), salvianolic acid O (**4**), nepetoidin A (**5**), salvianolic acid P (**6**), salvianolic acid Q (**7**), and nepetoidin B (**8**) identified in species of the Nepetoideae subfamily. Previously undescribed compounds are marked in red and indicated with an asterisk.

Rosmarinic acid (RA) is a polyphenolic specialized plant metabolite that is widely distributed across diverse plant taxa. Structurally, it is a dimeric compound composed of two phenylpropanoid (C6–C3) units, caffeic acid and 3-(3,4-dihydroxyphenyl)-2-hydroxypropanoic acid, linked by an ester bond (Figure 1). RA exhibits a broad range of promising biological activities, including antioxidant, anti-inflammatory, antidiabetic, antiproliferative, antiviral, hepatoprotective, and neuroprotective effects. Its high abundance is considered a chemotaxonomic characteristic of species in the Nepetoideae subfamily of the family Lamiaceae [1]. This subfamily includes numerous medicinal and aromatic plants widely used in phytotherapy and listed as official herbal drugs in pharmacopeias. Among Nepetoideae species, the leaves or aerial parts of *Mentha × piperita*, *Melissa officinalis*, *Origanum vulgare*, *Salvia officinalis*, and *Thymus vulgaris* are included in the European Pharmacopoeia (Ph. Eur.). The medicinal applications of these plants are largely attributed to their RA content, which often exceeds 2% of the dried plant material (Supplementary Table S1, [2,3,4,5,6,7,8,9,10,11,12,13,14,15,16,17,18,19,20,21,22,23,24,25,26,27]).

Several structurally related compounds are also characteristic metabolites of Nepetoideae species, including salvianolic acid (SAA) K, which differs from RA by the presence of an additional C6–C3 unit, namely 3-(3,4-dihydroxyphenyl)-2,3-dihydroxypropanoic acid; RA glucoside; and the nepetoidins (NEPs) A and B, which may theoretically be formed from RA through the loss of a formyl group (Figure 1). These compounds are generally regarded as minor metabolites, particularly NEP A and NEP B, which have only been detected in trace amounts (Supplementary Table S1). Previous phytochemical investigations have primarily been based on extracts prepared using organic solvents or hot water (Supplementary Table S1). Due to the limited availability of RA glucoside, SAA K, and NEP A, their biological activities have not been extensively studied. Although the total synthesis of NEP B has been reported, its biological activities have likewise remained largely unexplored [28,29]. Nevertheless, several activities have been reported, including xanthine oxidase inhibitory activity [19,21,23], and antifungal effects [20] for NEP A and NEP B; antioxidant activity for RA glucoside [30], and NEP B [24]; anti-inflammatory and antiviral activities for NEP B [22,27]; aldose reductase inhibitory activity for RA glucoside and SAA K [16], and efficacy of SAA K in diabetic nephropathy [31].

In addition to the Nepetoideae species officially recognized in the Ph. Eur., the aerial parts of several additional Nepetoideae species, such as *Mentha aquatica*, *Mentha spicata*, *Satureja hortensis*, and *Thymus serpyllum*, are widely used medicinally and have been extensively investigated phytochemically, confirming their high RA content (Supplementary Table S1). In contrast, four *Salvia* species common in Europe and Central Asia, *S. sclarea*, *S. nemorosa*, *S. pratensis*, and *S. verticillata*, have been less extensively studied for their specialized metabolites. However, their aerial parts are also used in traditional medicine [32]. These species possess well-developed root systems that, in addition to their aerial parts, may represent novel sources of bioactive compounds.

An alternative strategy for producing bioactive compounds is the cultivation of plants in in vitro biotechnological systems. Plants grown in vitro may exhibit metabolite profiles that differ substantially from those of field-grown plants, reflecting differences in the activity of enzymes involved in metabolite biosynthesis and biotransformation [33].

Many specialized plant metabolites are stored in plant tissues separately from the endogenous enzymes that can transform them. This compartmentalization plays a key role in plant defense against pathogens and pests, as tissue damage can bring metabolites and enzymes into contact, resulting in the formation of new compounds. Such transformation products may occur only in trace amounts or may be entirely absent in intact tissues, yet they can exhibit stronger protective activity than their precursors [34]. Similarly, mechanical disruption of plant tissues, for example, by suspending them in water at room temperature, may also trigger enzymatic transformations due to the loss of compartmentalization. Several such reactions have previously been demonstrated in our phytochemical studies, including the hydroxylation of phenylethanoids [35], the cleavage of saccharide units from lignan glycosides [36,37,38,39], and the removal of malonyl groups from lignans by ester hydrolysis [40].

Because RA and its related compounds (RA glucoside, SAA K, NEP A, and NEP B) contain ester bonds, and RA glucoside additionally contains a glycosidic bond susceptible to enzymatic hydrolysis, Nepetoideae plants may possess significant potential for enzymatic compound transformations, provided that the corresponding enzymes are present. Since previous phytochemical studies of Nepetoideae species have relied primarily on extraction with hot water or organic solvents, enzymatic transformations can largely be excluded because these conditions inactivate endogenous enzymes (Supplementary Table S1). Therefore, investigating the enzymatic conversion of RA and related compounds in aqueous suspensions prepared with room-temperature water is of particular importance. During the preparation of extracts under such conditions, the original phytochemical composition and bioactivity profile reported in the literature may change substantially due to enzymatic transformations.

The enzymatic conversion of compounds in aqueous media may also provide a practical approach for producing metabolites that are absent or present only in trace amounts in intact plant tissues [40]. Optimizing the incubation period may further improve the yield of these target compounds, thereby enhancing isolation efficiency.

Based on these considerations, the aims of the present study were to:

1. Monitor changes in the composition of RA and its derivatives in the medicinally relevant tissues of 13 selected Nepetoideae species (*Melissa officinalis*, *Mentha aquatica*, *M. × piperita*, *M. spicata*, *O. vulgare*, *Salvia sclarea*, *S. nemorosa*, *S. officinalis*, *S. pratensis*, *S. verticillata*, *Satureja hortensis*, *Thymus serpyllum*, and *T. vulgaris*) under different extraction conditions by analyzing the metabolite profiles of aqueous suspensions incubated for various time periods;
2. Isolate and identify potentially bioactive RA-related compounds from the selected tissues;
3. Evaluate the bioactive potential of the isolated compounds by assessing their in vitro antioxidant and metabolic activities; and
4. Highlight the importance of extraction conditions in promoting possible enzymatic transformations of RA and related compounds, thereby significantly influencing the metabolite composition of the resulting extracts.

## 2. Materials and Methods

### 2.1. General Experimental Procedures

Analytical HPLC with UV and High-Resolution Orbitrap Mass Spectrometry. A Dionex UltiMate 3000 HPLC system (3000RS diode array detector (DAD), TCC-3000RS column thermostat, HPG-3400RS pump, SRD-3400 solvent rack degasser, WPS-3000TRS autosampler) connected to an Orbitrap Q Exactive Focus Mass Spectrometer equipped with electrospray ionization (ESI) (Thermo Fisher Scientific, Waltham, MA, USA) was used. Column: Kinetex C18 column (75 × 3 mm; 2.6 μm) (Phenomenex, Torrance, CA, USA). Eluents: eluent A, 0.1% *v*/*v* formic acid, eluent B, acetonitrile:0.1% *v*/*v* formic acid (80:20, *v*/*v*). Gradient: 0.0 min, 10% B; 10.0 min, 50% B (linear gradient); 12.0 min, 90% B (linear gradient); 13.5 min, 90% B (isocratic). Flow rate: 0.3 mL/min; column temperature: 25 °C; injected volume: 2.0 μL. Ionization: The ESI source was operated in polarity-switching mode, acquiring data in both positive- and negative-ion modes. Fragmentations: data-independent acquisition (DIA) with isolation widths of 100–300 *m*/*z*, 295–500 *m*/*z*, 495–700 *m*/*z*, 695–850 *m*/*z*, and 845–1000 *m*/*z*, and collision energies of 15, 30, and 45 eV; parallel reaction monitoring (PRM) method with an isolation width of 0.4 *m*/*z*, and collision energies ranging from 10 eV to 45 eV. Resolution: full MS, 70,000; MS/MS, 35,000. Full MS scans range: *m*/*z* 100–1000. UV scan range: 230–600 nm. Each HPLC-UV chromatogram represents the sum of signal intensities detected within this range (other operating parameters were consistent with those described in our recent article [40].

Quantification of compounds: An external standard method was employed using an HPLC-UV technique. The compounds used as calibration standards were isolated in this study. Linear regression analyses were performed for the isolated compounds in the following concentration ranges: 0.250–100.0 µg/mL (**1**), 0.250–200.0 µg/mL (**2**), 0.20–400.0 µg/mL (**3**), 0.40–200.0 µg/mL (**4**), 0.20–100.0 µg/mL (**5**), 0.16–100.0 µg/mL (**6**), and 0.20–100.0 µg/mL (**8**), with the lower concentration values corresponding to the respective limits of quantitation (LOQs). The limits of detection (LODs), defined as UV signals exceeding three times the noise level, determined by HPLC–UV were 0.075 µg/mL for compounds **1** and **2**, 0.061 µg/mL for compounds **3**, **5**, and **8**, 0.12 µg/mL for compound **4**, and 0.048 µg/mL for compound **6**. The LODs determined by HPLC–MS by plotting selected-ion chromatograms of the deprotonated compounds in negative-ionization mode were identical to those determined by HPLC–UV, except for compound **8**, for which an LOD of 0.020 µg/mL was obtained. All compounds exhibited r^2^ values greater than 0.999. The quantities of compound **7** were calculated using the calibration curve of compound **6**, allowing only the semiquantitative determination of compound **7**. Compound contents are expressed on a dry plant tissue basis.

Stability of compounds: Isolated pure compounds (**1**–**6** and **8**) were dissolved in 80% (*v*/*v*) methanol at comparable concentrations. Two parallel mixture samples were prepared and analyzed by HPLC-UV-MS immediately after preparation, without heating. Aliquots of the same samples were then heated at 60 °C for 30 min and subsequently analyzed by HPLC-UV-MS under identical conditions. Comparison of the HPLC-UV peak areas of the corresponding compounds in the non-heated and heated samples revealed no significant changes in their amounts following heating. This was confirmed by the relative percent differences (RPDs) between the mean peak areas of the corresponding compounds in the non-heated and heated samples, which ranged from 0.3% (compound **3**) to 2.4% (compound **2**).

Isolation of Compounds by Preparative HPLC. A Dionex UltiMate 3000 HPLC System (VWD-3400RS photometer, HPG-3200BX pump, WPS-3000TSL autosampler, VF-F11-A-01 Fraction Collector F) (Thermo Fisher Scientific, Waltham, MA, USA) was used. Detection: 330 nm. Column: Gemini NX-C18 column (250 × 10 mm; 5 μm) (Phenomenex, Torrance, CA, USA). Eluents: eluent A, 0.1% *v*/*v* formic acid, eluent B, acetonitrile. Gradient program 1 for compounds **1**, **2**, **3**, and **4**: 0.0 min, 15% B; 5.0 min, 15.0% B (isocratic); 20.0 min, 30% B (linear gradient); 25.0 min, 35% B (linear gradient); 26.0 min, 80% B (linear gradient); 30.0 min, 80% B (isocratic). Gradient program 2 for compounds **5**, **6**, and **8**: 0.0 min, 15% B; 5.0 min, 15.0% B (isocratic); 20.0 min, 30% B (linear gradient); 35.0 min, 45% B (linear gradient); 37.0 min, 80% B (linear gradient); 40.0 min, 80% B (isocratic).

NMR Analysis. NMR spectra of the isolated compounds were recorded by using a Bruker Avance III HD 500 (500/125 MHz) spectrometer (Bruker Corp., Billerica, MA, USA) at 298 K. HSQC and HMBC spectra of compound **4** were recorded at 328 K. The NMR spectra of the compounds were recorded in various deuterated solvents, such as methanol-*d*_4_ (compounds **1**, **3**), acetone-*d*_6_ (compounds **5**, **8**), and DMSO-*d*_6_ + D_2_O (compounds **2**, **4**, **6**), to overcome severe line broadening of certain ^1^H NMR resonances. Spectra were acquired through Bruker TopSpin 3.6 software employing standard pulse sequences provided in the software library. Spectra were processed and analyzed by the MestReNova NMR processor. ^1^H and ^13^C chemical shifts (*δ*) are given in ppm relative to the residual solvent peaks (methanol-*d*_4_; 3.31/49.00 ppm, DMSO-*d*_6_; 2.50/39.52 ppm for ^1^H and ^13^C NMR respectively, and acetone-*d*_6_ 2.50 ppm for ^1^H NMR only), while coupling constants (*J*) are given in Hz. NMR resonance assignments were completed based on 1D 1H NMR, ^13^C NMR and 2D ^1^H–^1^H COSY, ^1^H–^1^H NOESY, ^1^H–^13^C HSQC, and ^1^H–^13^C HMBC experiments.

Optical rotation measurements. Optical rotations were measured in methanol at 25 °C on a Jasco P-200 polarimeter (Jasco Inc., Tokyo, Japan) using the sodium D line (589 nm).

Ultraviolet spectroscopy. λ*_max_* and specific absorbance (log ε) values of compounds were determined in MeOH by a Jasco V-550 UV/Vis spectrophotometer (Jasco Inc., Tokyo, Japan).

Structural characterization of previously undescribed isolated compounds **4** and **6**

Salvianolic acid O (**4**): light yellow amorphous solid; ${\left[\alpha \right]}_{D}^{25}$ = 56.9 (*c* 0.2, MeOH); UV (MeOH) λ_max_ (log ε) 320.5 nm (4.19), 288 nm (4.12); HRESIMS (negative) *m*/*z* 555.1144 [M − H]^−^ (calcd for 555.1144, C_27_H_23_O_13_); for all HR-Orbitrap-MS data, see Table 1; for ^1^H NMR and ^13^C NMR data, see Table 2. 

**Table 1:** Mass spectrometry (MS) data of compounds (**1**–**8**), determined by HPLC high-resolution Orbitrap-MS using negative and positive electrospray ionization (ESI) modes.

No.^a^	Formula	MS Mode ^b^	Detected Ion	Measured Mass (*m*/*z*)	Mass Error (ppm)
**1**	C_24_H_26_O_13_	+	[M + NH_4_]^+^	540.1705	−0.696
			[M + H]^+^	523.1437	−0.937
		−	[M − H]^−^	521.1301	0.006
**2**	C_27_H_24_O_13_	+	[M + Na]^+^	579.1096	−1.292
			[M + NH_4_]^+^	574.1547	−0.796
			[M + H]^+^	557.1279	−1.037
		−	[M − H]^−^	555.1144	−0.034
**3**	C_18_H_16_O_8_	+	[M + H]^+^	361.0909	−0.914
			[M + NH_4_]^+^	378.1175	−0.823
			[M + Na]^+^	383.0728	−0.959
		−	[M − H]^−^	359.0773	0.089
**4**	C_27_H_24_O_13_	+	[M + Na]^+^	579.1095	−1.412
			[M + NH_4_]^+^	574.1545	−0.976
			[M + H]^+^	557.1276	−1.407
		−	[M − H]^−^	555.1144	−0.034
**5**	C_17_H_14_O_6_	+	[M + H]^+^	315.0858	−1.591
		−	[M − H]^−^	313.0720	0.259
**6**	C_26_H_22_O_11_	+	[M + Na]^+^	533.1050	−0.393
			[M + NH_4_]^+^	528.1493	−0.687
			[M + H]^+^	511.1229	−0.608
		−	[M − H]^−^	509.1090	0.045
**7**	C_26_H_22_O_11_	+	[M + Na]^+^	533.1047	−0.733
			[M + NH_4_]^+^	528.1490	−1.077
			[M + H]^+^	511.1230	−0.478
		−	[M − H]^−^	509.1092	0.225
**8**	C_17_H_14_O_6_	+	[M + H]^+^	315.0859	−0.405
		−	[M − H]^−^	313.0720	0.259
			[M − HCOO]^−^	359.0775	0.269

^a^ Compound numbers correspond to rosmarinic acid-3-O-β-D-glucoside (**1**), salvianolic acid K (**2**), rosmarinic acid (**3**), salvianolic acid O (**4**), nepetoidin A (**5**), salvianolic acid P (**6**), salvianolic acid Q (**7**), and nepetoidin B (**8**). ^b^ High-resolution mass spectrometry (HR-MS) detection was operated in positive (+) and negative (−) ionization modes.

**Table 2:** ^1^H and ^13^C NMR data (*δ* in ppm, *J* in Hz) of compounds **1**–**4**. Spectra were recorded in methanol-*d*_4_ (**1**, **3**) and DMSO-*d*_6_/D_2_O (**2**, **4**) at 500/125 MHz, except for compound **1** (400/100 MHz).

No	1		2		3		4	
	*δ*_H_	*δ*_C_	*δ*_H_	*δ*_C_	*δ*_H_	*δ*_C_	*δ*_H_	*δ*_C_
1	-	127.9	-	128.7	-	127.6	-	125.52
2	7.54, d (2.0)	118.05	7.09, d (2.1)	115.37	7.04, d (2.1)	115.2	7.03, br s	** 116.33
3	-	147.1	-	* 148.13	-	146.81	**-**	** 151.1
4	-	151.1	-	148.9	-	149.7	**-**	146.5
5	6.87, d (8.3)	117.41	6.62, m (ov)	115.73	6.78, d (8.2)	116.49	6.80, d (8.1)	116.65
6	7.19, dd (8.4, 2.0)	126.2	7.01, dd (8.5, 2.2)	121.2	6.95, dd (8.2, 2.1)	123.1	7.15, dd (8.4, 2.0)	125.25
7	7.59, d (15.9)	146.9	7.45, d (15.9)	145.73	7.55, d (15.9)	* 147.6	7. 43, d (15.8)	145.95
8	6.38, d (15.9)	115.6	6.34, d (16.0)	115.58	6.27, d (15.9)	114.4	6.26, d (16.0)	114.1
9	-	168.4	-	166.2	-	168.4	-	166.3
1′	-	129.6	-	127.6	-	129.3	-	127.6
2′	6.76, d (2.1)	117.65	6.67, m (ov)	116.9	6.75, d (2.1)	117.5	6.66, m (ov)	116.89
3′	-	146.07	-	145.04	-	146.16	-	145.05
4′	-	145.2	-	144.13	-	145.3	-	144.13
5′	6.69, d (8.0)	116.3	6.64, m (ov)	115.73	6.70, d (8.1)	116.28	6.64, d (8.0)	115.74
6′	6.62, dd (8.1, 2.1)	121.8	6.52, dd (8.1, 2.1)	120.5	6.61, dd (8.1, 2.1)	121.7	6.53, dd (8.1, 2.1)	120.5
7′	2.99, dd (14.3, 8.6) 3.09, dd (14.3, 4.1)	38.07	2.89, dd (14.4, 8.5) 2.97, dd (14.4, 4.3)	36.3	3.00, dd (14.3, 8.4) 3.10, dd (14.3. 4.3)	37.9	2.90, dd (14.4, 8.5) 2.98, dd (14.4, 4.4)	36.3
8′	5.16, dd (8.6. 4.0)	* 75.26	5.02, dd (8.3, 4.3)	73.2	5.18, dd (8.4, 4.3)	74.7	5.03, dd (8.4, 4.5)	73.26
9′	-	n.d.	-	171.12	-	173.6	-	** 171.16
1″	4.82, m (ov)	104.2	-	132.4	-	-	-	132.3
2″	3.51, m (ov)	74.92/77.61/78.52	6.87, d (2.0)	115.01	-	-	6.88, d (2.0)	115.10
3″	3.51, m (ov)	74.92/77.61/78.52	-	144.71/144.72	-	-	-	144.69
4″	3.39, m (ov)	71.52	-	144.71/144.72	-	-	-	144.71
5″	3.51, m (ov)	74.92/77.61/78.52	6.66, m (ov)	115.16	-	-	6.67, m (ov)	115.10
6″	3.72, m 3.97, dd (12.1, 2.2)	62.54	6.72, dd (8.2, 1.9)	118.7	-	-	6.73, dd (8.2, 2.0)	118.7
7″	-	-	4.81, d (6.0)	73.09	-	-	4.83, d (5.8)	73.13
8″	-	-	4.49, br s	* 83.2	-	-	4.65, br s	** 83.2
9″	-	-		* 171.46	-	-		** 171.49

* Signals deduced from HSQC and HMBC spectra; ** Signals deduced from HSQC and HMBC spectra recorded at elevated temperature (328 K), n.d., not detected.

Salvianolic acid P (**6**): light yellow amorphous solid; ${\left[\alpha \right]}_{D}^{25}$ = −37.2 (*c* 0.2, MeOH); UV (MeOH) λ_max_ (log ε) 331 nm (4.32), 300 nm (4.31), 249.5 nm (3.41); for ^1^H NMR and ^13^C NMR data, see Table 3; HRESIMS (negative) *m*/*z* 509.1090 [M − H]^−^ (calcd for 509.1089, C_26_H_21_O_11_); for all HR-Orbitrap-MS data, see Table 1.

### 2.2. Materials and Reagents

All materials and reagents used for compound analysis and isolation, maintenance of in vitro plant cultures, and in vitro bioactivity assays were of analytical grade and of the highest purity available. Acetonitrile, distilled water, formic acid, and methanol were purchased from Reanal (Budapest, Hungary). Acetone-*d*_6_, D_2_O, and DMSO-*d*_6_ were obtained from VWR Chemicals (Leuven, Belgium). Ascorbic acid, chlorogenic acid, DPPH (2,2-diphenyl-1-picrylhydrazyl), Murashige-Skoog medium, and Trolox were sourced from Sigma-Aldrich (Steinheim, Germany). Trypan blue, L-glutamine, phosphate-buffered saline (PBS), and Dulbecco’s Modified Eagle’s Medium (DMEM) were obtained from Lonza (Basel, Switzerland). Penicillin-streptomycin, nonessential amino acids, and trypsin were supplied by Gibco (Thermo Fisher Scientific, Waltham, MA, USA), while fetal bovine serum (FBS) was purchased from EuroClone (Pero, Italy).

### 2.3. Plant Material

Plant samples of *Mentha aquatica* L., *Origanum vulgare* L., *Salvia nemorosa* L., *Salvia pratensis* L., *Salvia verticillata* L., and *Thymus serpyllum* L. were collected from natural populations at various locations in Hungary during the flowering stage between 2022 and 2025. *Mentha × piperita* L., *Mentha spicata* L., *Salvia sclarea* L., *Salvia officinalis* L., *Satureja hortensis* L., and *Thymus vulgaris* L. were cultivated in home gardens from plants purchased from commercial horticultural nurseries in Hungary and were likewise collected during the flowering stage between 2022 and 2025. All plant materials were botanically identified at the Department of Plant Anatomy, Eötvös Loránd University (ELTE), Budapest, Hungary.

At each sampling time, at least five individual plants were collected. Plant organs were manually separated and pooled to prepare root, leaf, and aerial-part samples, which were lyophilized on the day of collection. Voucher specimens of all dried samples have been deposited at the Department of Plant Anatomy, Eötvös Loránd University, Budapest, Hungary. Prior to extraction, all samples were pulverized using a coffee grinder.

Commercial filter teas. Commercial filter teas of *Melissa officinalis* L., *Mentha × piperita* L., and *Salvia officinalis* L. were obtained from a local pharmacy and complied with pharmacopeial quality requirements.

In vitro-grown plant material. Actively growing apical and axillary shoot tips (approximately 2 cm long) were collected from mint plants. After leaf removal, the explants were washed under running tap water for 10 min with Tween 20 added as a wetting agent. Surface sterilization was carried out by immersion in a sodium hypochlorite solution for 5 min, followed by three rinses in sterile distilled water (15 min each) on a shaker. Under aseptic conditions in a laminar-flow cabinet, fresh cut surfaces were prepared using sterile instruments, and the explants were transferred onto solid Murashige-Skoog medium [41]. Cultures were maintained at 22 ± 1 °C under a 12 h light/12 h dark photoperiod and a photon flux density of 100 μmol m^−2^ s^−1^. Explants were subcultured onto fresh Murashige-Skoog medium every 28 days. Each species was represented by five plants independently grown in vitro. The aerial parts were lyophilized immediately after harvesting.

### 2.4. Preparation of Plant Extracts for Analysis and Isolation

Extraction of untreated samples for metabolite analysis. Lyophilized and pulverized plant tissues (20.0 mg; pulverized for 2 min using a conventional laboratory coffee grinder) were extracted with 3.0 mL of 80% (*v*/*v*) methanol at 60 °C for 30 min in 5 mL screw-capped vials. The extracts were centrifuged at 5000 rpm and diluted with 80% (*v*/*v*) methanol prior to HPLC-UV-HR-MS analysis.

Sample treatments for investigating the effects of endogenous enzymes on metabolite composition.

Treatment 1. Pulverized tissues (20.0 mg) were suspended in 600 µL distilled water by vortexing for 10 s and then incubated at room temperature for 5, 15, 30, 45, 60, or 135 min.

Treatment 2. Pulverized tissues (20.0 mg) were suspended in 600 µL distilled water by vortexing for 10 s, incubated at room temperature for 15 min, heated at 100 °C for 2 min, and subsequently incubated at room temperature for an additional 120 min.

Treatment 3. Pulverized tissues (20.0 mg) were suspended in hot distilled water by vortexing for 10 s, heated at 100 °C for 2 min, and subsequently incubated at room temperature for 45 or 60 min.

Following Treatments 1–3, 2.4 mL of absolute methanol was added to each sample to obtain a final methanol concentration of 80% (*v*/*v*). Samples were then heated at 60 °C for 30 min in 5 mL screw-capped vials, centrifuged at 5000 rpm, and diluted with 80% (*v*/*v*) methanol prior to HPLC-UV-HR-MS analysis.

Extraction procedures for compound isolation. Isolation of compounds **1**–**4**. Pulverized plant material (10 g; pulverized for 2 min using a conventional laboratory coffee grinder) was extracted with 50 mL of 80% (*v*/*v*) methanol at 60 °C for 30 min in screw-capped vials. After centrifugation at 5000 rpm, the residue was re-extracted with an additional 50 mL of 80% (*v*/*v*) methanol under identical conditions. The combined supernatants were concentrated to dryness using a rotary vacuum evaporator. The residue was dissolved in 8 mL methanol and centrifuged at 5000 rpm. The resulting supernatant was filtered through Minisart RC 15 syringe filters (0.2 µm pore size; Sartorius AG, Göttingen, Germany) and subjected to preparative HPLC. Pulverized root tissue of *S. verticillata* (Sample 3) was used for the isolation of compounds **1**, **3**, and **4**, whereas pulverized root tissue of *S. pratensis* (Sample 1) served as the source of compound **2**.

Isolation of compounds **5**, **6**, and **8**. Pulverized plant material (10 g; pulverized for 2 min using a conventional laboratory coffee grinder) was suspended in 40 mL distilled water by vortexing for 10 s and then incubated at room temperature for 30 min (compounds **5** and **8**) or 5 min (compound **6**). Subsequently, 50 mL of methanol was added, and the samples were heated at 60 °C for 30 min. After centrifugation at 5000 rpm, the residue was extracted a second time with 50 mL of methanol at 60 °C for 30 min, then centrifuged again. The combined supernatants were processed as described above to isolate compounds **1**–**4**. Pulverized leaf tissue of *S. officinalis* (Sample 2) was used for the isolation of compounds **5** and **8**, whereas pulverized root tissue of *S. sclarea* (Sample 2) was used for the isolation of compound **6**.

### 2.5. In Vitro Bioactivity Assays

Antioxidant activity. Radical scavenging activity was evaluated using the DPPH assay according to a previously published protocol [42]. Serial dilutions of the test compounds, prepared in methanol, were incubated with a DPPH solution at room temperature for 6 min. Absorbance was measured at 515 nm using a HITACHI U-2000 spectrophotometer (Hitachi Ltd., Tokyo, Japan). Half-maximal inhibitory concentration (IC_50_) values were calculated, and all measurements were performed in triplicate. The statistical significance of differences among the IC_50_ values of the investigated compounds was determined by one-way analysis of variance (ANOVA), followed by Tukey’s post hoc test. Prior to analysis, data normality was verified using the Shapiro–Wilk test. A *p*-value of less than 0.05 was considered statistically significant. Statistical computations were performed using GraphPad Prism 8.0.1.

Activity of the compounds on different tumorous cell cultures. The in vitro activity of the test compounds was evaluated using an Alamar Blue endpoint cell viability assay [43]. Cell cultures were maintained under standard culture conditions appropriate for each cell type. MM6 (MonoMac-6, human monocytic leukemia-derived line with monocyte-like features) and HT-29 (human colorectal adenocarcinoma epithelial cell line) cells were cultured in RPMI-1640 medium, whereas H838 (human lung adenocarcinoma, non-small cell lung cancer line), U87 (human glioblastoma/glioma cell line), and U251 (human glioblastoma/glioma cell line) cells were maintained in Dulbecco’s Modified Eagle’s Medium (DMEM). Both media were supplemented with 10% fetal bovine serum (FBS), 2 mM L-glutamine, 50 IU/mL penicillin, and 50 μg/mL streptomycin. In addition, DMEM was supplemented with 1 mM sodium pyruvate and 1% (*v*/*v*) non-essential amino acid solution. Cultures were maintained at 37 °C in a humidified atmosphere containing 5% CO_2_. Cell lines were obtained from authenticated laboratory stocks (HT-29, U87, and U251 were generous gifts from Prof. József Tóvári, National Institute of Oncology, Budapest, Hungary; MM6 was purchased from DSMZ). Cultures were checked for mycoplasma contamination before experiments and were found to be negative. The cells were grown to confluence, then divided into 96-well tissue culture plates at an initial cell density of 5.0 × 10^3^ cells/well. After 24 h of incubation at 37 °C, the cells were treated with the compounds in 200 μL final volume at 0.16–100 μM concentration overnight. Control cells were treated with serum-free medium only under the same conditions. After this incubation period, cells were washed twice with serum-free medium, and following that, they were cultured for another 72 h in 10% serum-containing medium at 37 °C. Each concentration was measured in four parallel wells, and the experiment was performed in two independent biological repeats. The Alamar Blue assay is based on the reduction in the blue, weakly fluorescent redox indicator, resazurin, to the pink, highly fluorescent product, resorufin, by metabolically active cells. In the endpoint assay format used in this study, a decrease in fluorescence intensity following treatment reflects reduced cellular metabolic activity and/or a reduction in the number of viable cells relative to untreated control cultures. Resazurin sodium salt (Merck, Darmstadt, Germany) was dissolved in PBS at a concentration of 0.15 mg/mL, pH 7.4. 32.5 uL of the dye was added to each well and incubated at 37 °C for 3 h until the pink color of the reduced dye appeared. Fluorescence intensity in each well was measured using a Synergy H4 multimode microplate reader (BioTek, Winooski, VT, USA); at λex = 530/30 and λem = 610/10 nm. The decrease in cellular metabolic activity/apparent cytostatic effect was calculated relative to the untreated control according to: Alamar Blue signal reduction (%) = [1 − (fluorescence intensity_treated/fluorescence intensity_control)] × 100. IC_50_ values were obtained by fitting a sigmoid concentration-response curve to the data points using Microcal Origin/OriginPro software (version 2018) and calculating the X value at Y = 50. IC_50_ values are reported in µM. Compounds that did not reduce the Alamar Blue signal by at least 50% within the tested range are reported as IC_50_ > 100 µM.

## 3. Results and Discussion

### 3.1. Enzymatic Conversion of Rosmarinic Acid into Nepetoidins A and B in the Leaves of Salvia officinalis During Aqueous Extraction

To reveal possible enzymatic conversions of RA, dried, pulverized leaf samples of *S. officinalis* were analyzed after their extracts were prepared using three different methods (Sample 1; all related measurements, including replicate measurements, are provided in Supplementary Table S2A). Firstly, pulverized tissues were suspended in distilled water and incubated at room temperature for varying durations. Secondly, pulverized tissues were suspended in hot distilled water and incubated at 100 °C for 2 min, then incubated at room temperature for an additional 60 min. After the aqueous incubation steps, absolute methanol (MeOH) was added to the suspensions to obtain a final concentration of 80% (*v*/*v*) MeOH. The samples were subsequently heated at 60 °C to extract the compounds for HPLC-HR-MS-UV analysis. In the third extraction method, pulverized tissues were extracted directly with 80% MeOH at 60 °C without prior water treatment, yielding extracts from untreated tissue samples.

**Figure 2:**
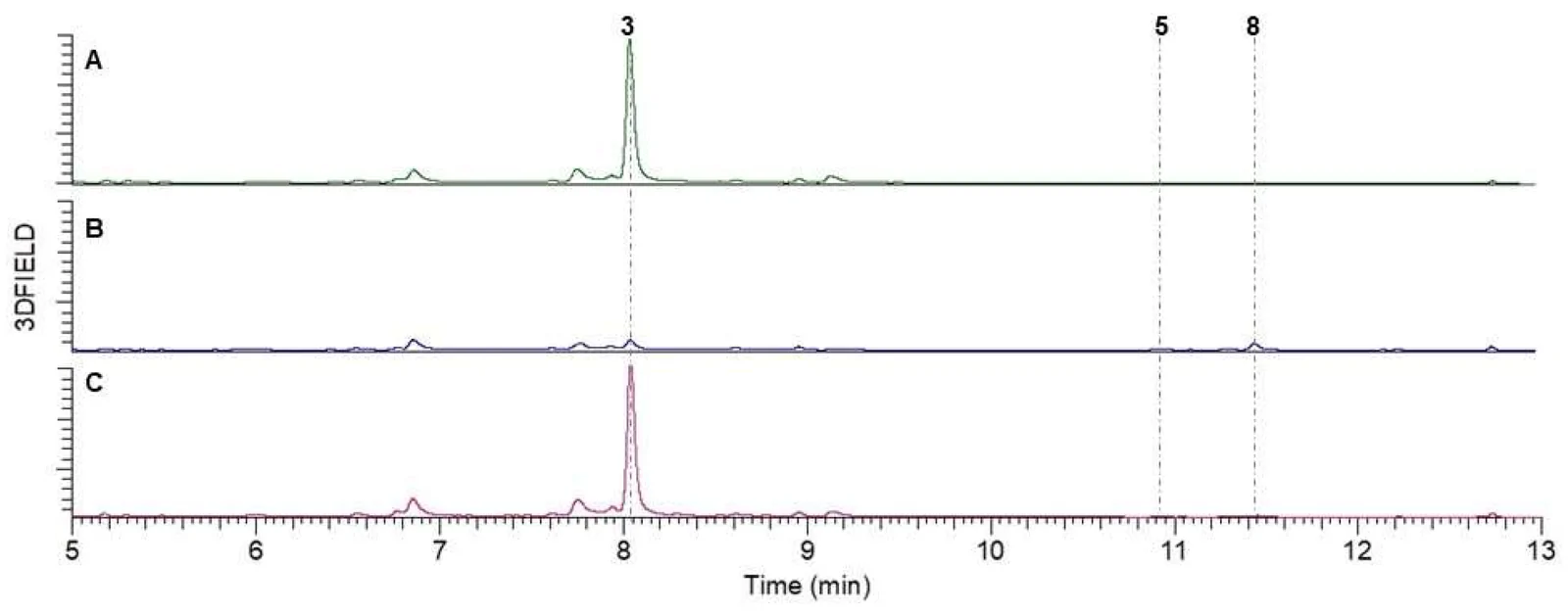
HPLC-UV chromatograms of *Salvia officinalis* leaf extracts prepared variously. A: pulverized tissues were extracted directly with 80% MeOH; B: pulverized tissues were suspended in distilled water and incubated at room temperature for 15 min; C: pulverized tissues were suspended in hot distilled water and incubated at 100 °C for 2 min, then incubated at room temperature for an additional 60 min. After the aqueous incubation steps (extracts B and C), absolute methanol was added to the suspensions to obtain a final concentration of 80% (*v*/*v*) methanol. Extracts A, B, and C correspond to samples SO/1/UT, SO/1/DW15, and SO/1/100 °C-DW60 in Supplementary Table S2A, respectively, where their quantitative compound compositions are detailed. Compound numbers correspond to rosmarinic acid (**3**), nepetoidin A (**5**), and nepetoidin B (**8**).

**Figure 3:**
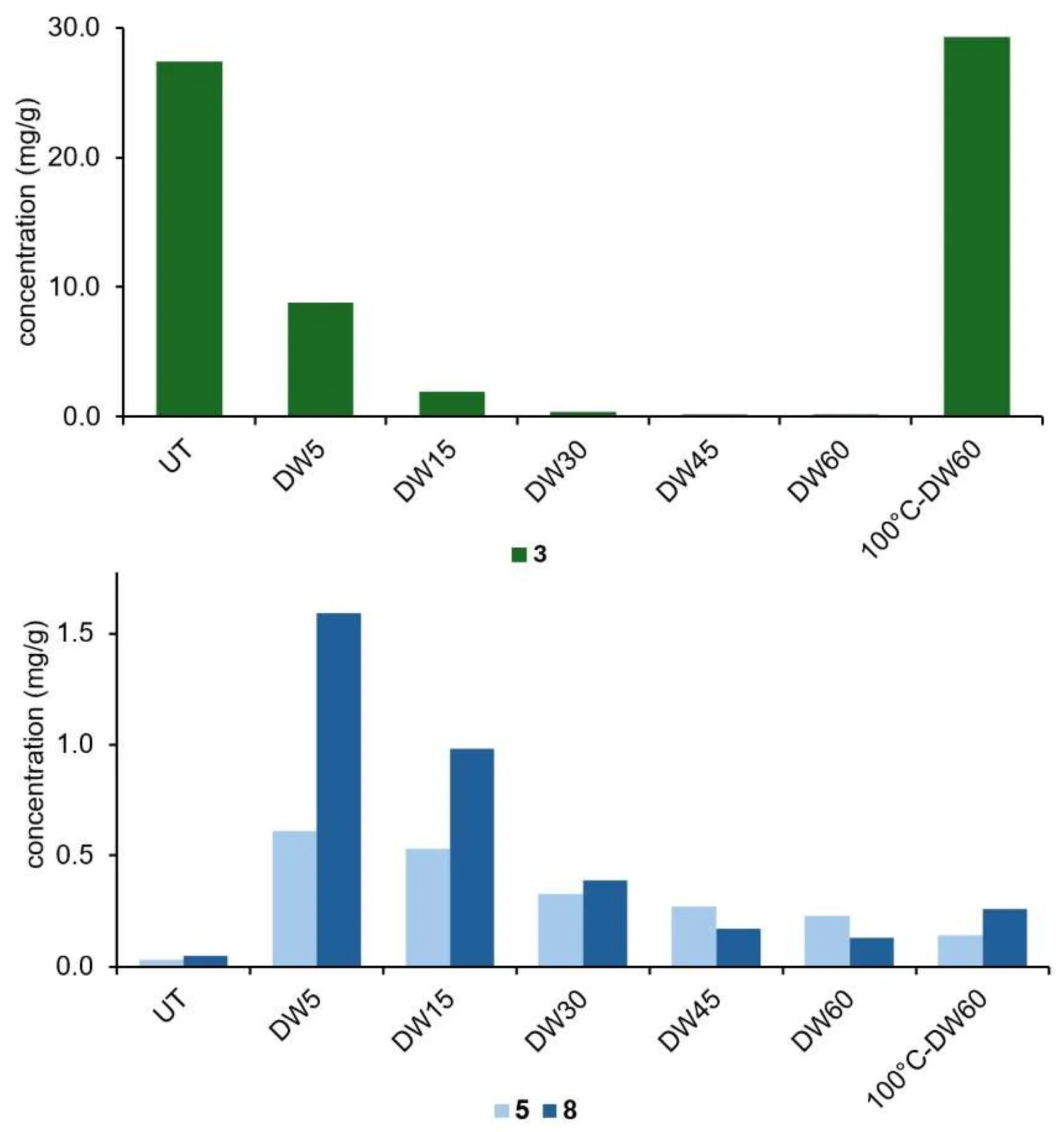
Contents of rosmarinic acid (**3**) and nepetoidins A (**5**) and B (**8**) in the dried leaf tissues of *Salvia officinalis* subjected to various extraction procedures. UT: untreated, prepared directly with 80% MeOH; DW5–DW60: tissues suspended in distilled water and incubated at room temperature for 5, 15, 30, 45, and 60 min, respectively; 100 °C-DW60: tissues suspended in hot distilled water, incubated at 100 °C for 2 min, and subsequently kept at room temperature for an additional 60 min. Following the aqueous incubation steps, absolute methanol was added to the suspensions to achieve a final concentration of 80% (*v*/*v*). These samples correspond to SO/1/UT, SO/1/DW5–DW60, and SO/1/100 °C-DW60 in Supplementary Table S2A. Results for samples UT and DW15 represent the mean values obtained from HPLC–UV analyses of two independently prepared parallel extracts. The corresponding relative percent difference (RPD) values for the UT and DW15 samples, respectively, were 1.8% and 10.7% for RA, 0.0% and 7.6% for NEP A, and 22.2% and 1.0% for NEP B.

Comparison of the chromatograms obtained from the three extraction methods revealed characteristic differences in their peak profiles (Figure 2). The 80% MeOH extract prepared without prior water treatment (untreated sample) contained compound **3** as the major constituent (Figure 2, chromatogram A). Compound **3** was identified as RA based on HR-MS and UV spectral data, which matched literature reports (Table 1, Supplementary Figures S1 and S2). Following its isolation, the exact structure was confirmed by NMR analysis (Section 3.8). The concentration of RA in the plant samples was determined by HPLC-UV using a calibration curve based on the relationship between peak area and the concentration of isolated RA (Supplementary Figures S3 and S4). In agreement with previous studies reporting RA as the major constituent of the leaves and aerial parts of *S. officinalis* at high concentrations ranging from 9–39 mg/g on a dry weight basis (Supplementary Table S1), its content in the untreated sample was determined to be 27.4 mg/g with a relative percent difference (RPD) of 1.83% (Figure 3, sample UT and Supplementary Table S2A, sample SO/1/UT). Samples incubated in water at room temperature for 5, 15, 30, 45, and 60 min showed a significant decrease in RA content as incubation time increased (Figure 3).

After a 15-min incubation, only 1.97 mg/g RA (RPD = 10.7%) was detected, while after 60 min, less than 1/180 of the initial quantity remained (0.15 mg/g) (Figure 3, Supplementary Table S2A). The relatively rapid decomposition of RA in an aqueous medium suggests enzyme-mediated conversion rather than degradation traceable to chemical instability.

The RA content of the pulverized tissue sample heated at 100 °C for 2 min prior to a 60 min incubation in water at room temperature was comparable to that of the untreated samples (29.3 and 27.4 mg/g, respectively; Figure 3, Supplementary Table S2A). This finding indicates that the time-dependent conversion of RA observed in the aqueous medium at room temperature was mediated by endogenous enzyme(s) present in the plant tissues. The preservation of RA following the heat treatment suggests that these enzymes were effectively inactivated at 100 °C.

The levels of two low-abundance compounds, **5** and **8**, also changed during aqueous incubation of the samples at room temperature (Figure 3). Based on their HR-MS and UV spectral data, which were consistent with published literature, compounds **5** and **8** were identified as the isomeric phenylpropanoids NEP A and NEP B, respectively (Table 1, Supplementary Figures S1 and S2). Both compounds have previously been reported as minor metabolites in the leaves of *S. officinalis* (Supplementary Table S1). NEP A and NEP B were isolated from their respective optimal sources following the optimized water-treatment procedure described in Section 3.5 (Supplementary Figure S4).

The exact structures of the isolated compounds were confirmed by NMR analysis (Section 3.8). Subsequently, their quantification in dried leaf tissues was performed by HPLC–UV, using calibration curves of the isolated compounds (Supplementary Figures S3 and S4). Consistent with previous reports, untreated leaf samples contained only trace amounts of NEPs A and B (0.03 mg/g and 0.05 mg/g dry weight, with RPDs of 0.0% and 22.2%, respectively). However, incubation of leaf tissue in water at room temperature for 5 min significantly increased their concentrations to 0.61 mg/g and 1.59 mg/g, respectively, representing approximately 20-fold and 30-fold increases relative to the untreated samples. Prolonged incubation in room-temperature water resulted in a gradual decline in the levels of both compounds (Figure 3, Supplementary Table S2A).

Considering the enzymatic decomposition of RA, the formation of NEPs A and B followed by their rapid decline during aqueous incubation, and the close structural relationship between RA and the NEPs, it is reasonable to propose that NEPs are generated from RA and subsequently undergo further enzymatic degradation by endogenous enzymes. Accordingly, the maximum accumulation of NEPs during tissue incubation in water at room temperature is likely governed by the balance between the enzymatic conversion of RA into NEPs and the subsequent enzymatic degradation of NEPs.

NEPs have previously been considered unstable compounds based on observations that their levels decreased during the drying of plant tissues [6]. However, their chemical stability during drying has subsequently been demonstrated, and the apparent loss of NEPs from dried basil was attributed to reduced extractability with organic solvents rather than to their degradation [6].

Comparing the maximum combined concentration of NEPs A and B measured in the water-treated tissue after 5 min (0.61 mg/g + 1.59 mg/g = 2.20 mg/g) with their combined concentration in the untreated tissue (0.03 mg/g + 0.05 mg/g = 0.08 mg/g), the net accumulation during water incubation was 2.12 mg/g (2.20 − 0.08 mg/g). Given that NEPs A and B have the same molecular weight (314 g/mol), this increase corresponds to 6.75 µmol/g (2.12 mg/g divided by the molecular weight of NEPs A and B). The untreated tissue contained 27.4 mg/g RA, corresponding to 76.1 µmol/g (27.4 mg/g divided by the molecular weight of RA, 360 g/mol). Thus, the amount of NEPs formed during incubation (6.75 µmol/g) represents only 8.9% of the maximum theoretical yield obtainable from the RA pool present in the untreated tissue. This low conversion efficiency suggests that extensive enzymatic degradation of NEPs occurred during incubation.

However, it remains necessary to confirm that the degradation of NEPs is enzymatically driven rather than a result of chemical instability in aqueous media. Investigating the conversion of RA into NEPs A and B, using aerial parts of three *Mentha* species cultivated both in vitro and under field conditions, could address this question.

### 3.2. Enzymatic Conversion of Rosmarinic Acid into Nepetoidins A and B in the Aerial Parts of Mentha Species During Aqueous Extraction

**Figure 4:**
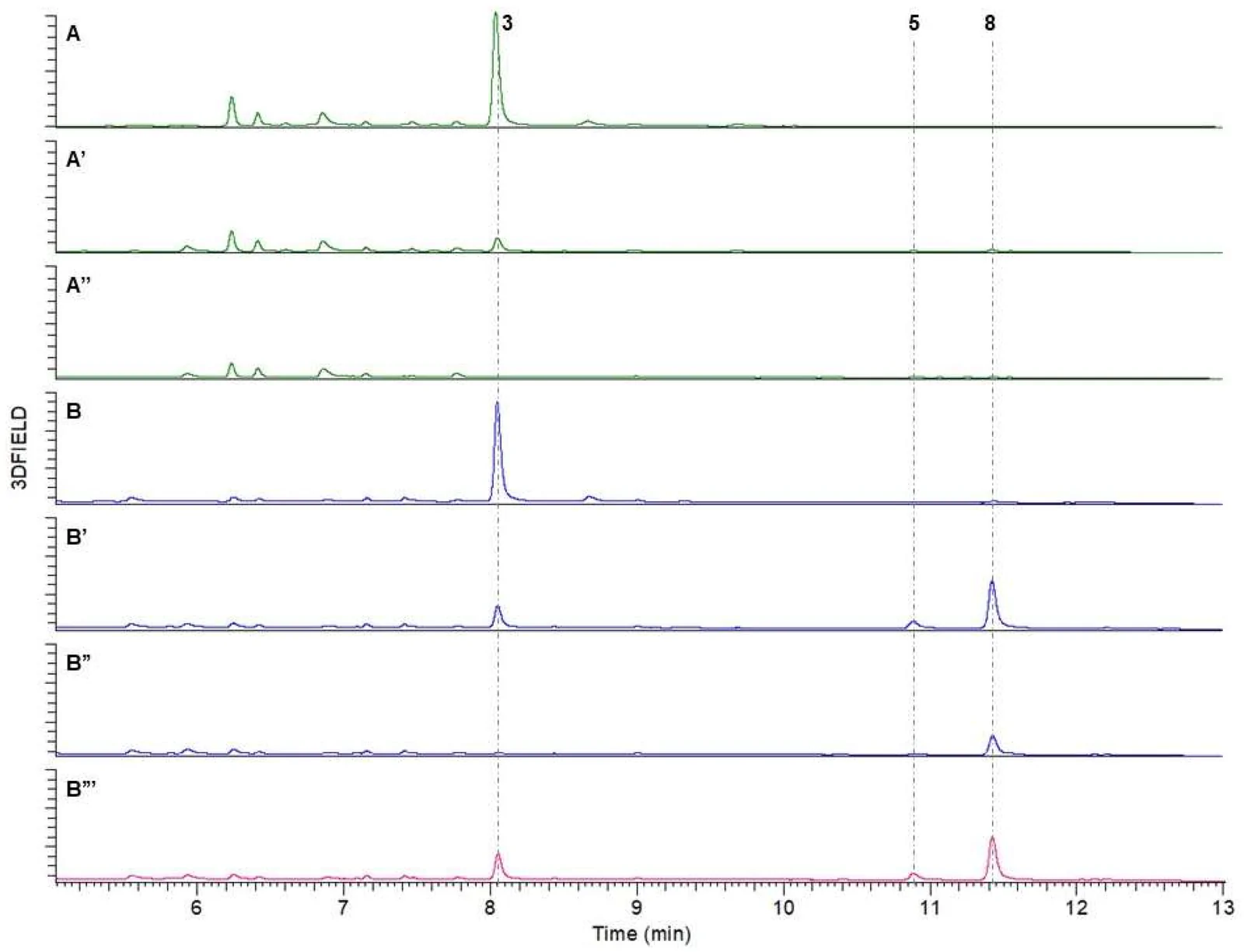
HPLC-UV chromatograms of extracts prepared from field-grown (A, A′, and A″) and in vitro-grown (B, B′, B″, and B‴) aerial parts of *Mentha aquatica* using different extraction procedures. Pulverized tissues were extracted directly with 80% MeOH. Alternatively, pulverized tissues were suspended in distilled water and incubated at room temperature for 15 min (A′ and B′), 45 min (A″), or 135 min (B″). In vitro-grown tissues were also subjected to a treatment consisting of incubation in distilled water at room temperature for 15 min, heating at 100 °C for 2 min, and a subsequent incubation at room temperature for 120 min (B‴). Following the aqueous incubation steps, absolute methanol was added to the suspensions to obtain a final methanol concentration of 80%. Chromatograms A, A′, and A″ correspond to samples MA/FG/1/UT, MA/FG/1/DW15, and MA/FG/1/DW45, respectively, whereas chromatograms B, B′, B″, and B‴ correspond to samples MA/IV/1/UT, MA/IV/1/DW15, MA/IV/1/DW135, and MA/IV/1/DW15-100 °C-DW120, respectively, in Supplementary Table S2B, where their quantitative phenylpropanoid compositions are provided. Compound numbers correspond to rosmarinic acid (**3**), nepetoidin A (**5**), and nepetoidin B (**8**).

**Figure 5:**
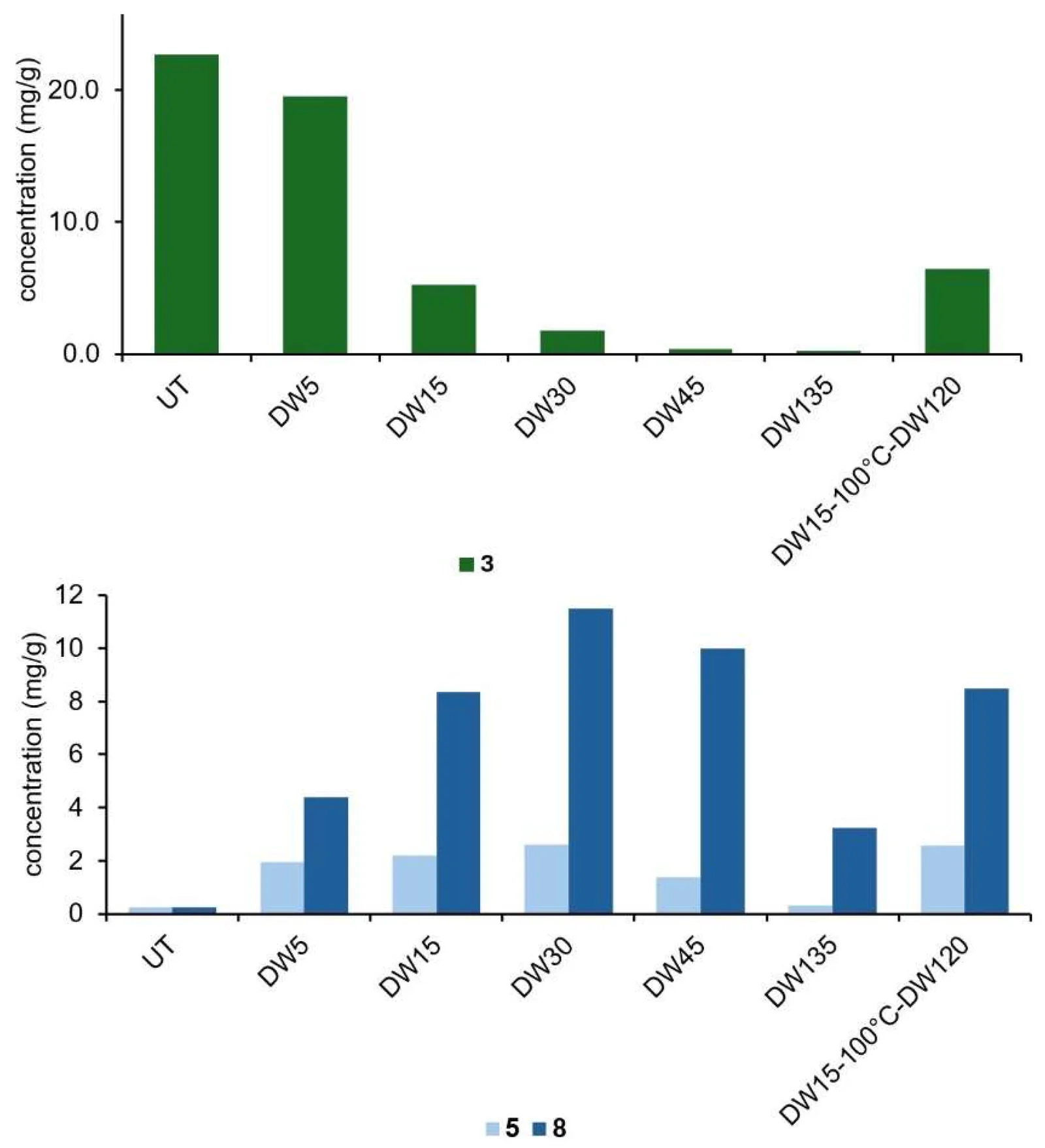
Contents of rosmarinic acid (**3**) and nepetoidins A (**5**) and B (**8**) in in vitro-grown aerial parts of *Mentha aquatica* subjected to various extraction procedures. UT: tissues extracted directly with 80% MeOH. DW5–DW135: tissues suspended in distilled water and incubated at room temperature for 5, 15, 30, 45, or 135 min before methanol extraction. DW15-100 °C-DW120: tissues suspended in distilled water, incubated at room temperature for 15 min, heated at 100 °C for 2 min, and subsequently incubated at room temperature for 120 min. Following the aqueous incubation steps, absolute methanol was added to obtain a final methanol concentration of 80% (*v*/*v*). These samples correspond to MA/IV/1/UT, MA/IV/1/DW5–DW135, and MA/IV/1/DW15-100 °C-DW120, respectively, in Supplementary Table S2B, where their quantitative phenylpropanoid compositions are provided. Results for samples UT, DW15, DW135, and DW15-100 °C-DW120 represent the mean values obtained from HPLC–UV analyses of two independently prepared parallel extracts. The relative percent difference (RPD) values ranged from 0.76% to 8.33% for RA, from 0.78% to 4.3% for NEP A, and from 0.0% to 10.9% for NEP B across these samples.

High concentrations of RA, coupled with negligible amounts of NEPs A and B, were detected in the field-grown *M. aquatica*, *M. × piperita*, and *M. spicata* samples, confirming previous findings (Supplementary Tables S1 and S2B). Room-temperature incubation in an aqueous medium yielded results comparable to those reported for *S. officinalis*: rapid decomposition of RA, followed by the formation and subsequent degradation of NEPs A and B (Figure 4 and Figure 5, Supplementary Table S2B). The enzyme-catalyzed nature of this transformation was confirmed by preheating the samples to 100 °C, which completely prevented RA degradation during subsequent room-temperature incubations (all related measurements, including replicate measurements, are provided in Supplementary Table S2B).

Among the field-grown *Mentha* tissue samples, the highest concentrations of NEPs A and B were detected after 5 or 15 min of incubation in water. Specifically, the total amounts reached 2.43 mg/g in *M. aquatica* (5 min), 0.69 mg/g and 1.10 mg/g in two *M. × piperita* samples (15 min), and 0.55 mg/g and 1.44 mg/g in two *M. spicata* samples (5 and 15 min) (Supplementary Table S2B). Assuming NEPs A and B are generated from RA during water treatment, these yields represent only 5–15% of the maximum theoretical yield calculated from the RA content of the corresponding intact, untreated samples.

In contrast, in vitro-grown *Mentha* samples yielded a significantly higher proportion of NEPs A and B after incubation in room-temperature water (Figure 4 and Figure 5; Supplementary Table S2B). Relative to the RA content in untreated in vitro samples (*M. × piperita*: 8.59 mg/g; *M. spicata*: 10.9 mg/g; *M. aquatica* Sample 1: 22.7 mg/g (RPD = 2.2%); Sample 2: 17.3 mg/g), the maximum values for NEPs A and B after a 15 min incubation were 0.62 mg/g and 4.18 mg/g for *M. × piperita*, 0.60 mg/g and 6.13 mg/g for *M. spicata*, 2.38 mg/g and 7.87 mg/g for *M. aquatica* (Sample 2), respectively. For *M. aquatica* (Sample 1), the maximum values were 2.61 mg/g and 11.5 mg/g after a 30 min incubation. Consequently, in the water-treated samples, approximately 10% and more than 50% of the RA initially present in the untreated tissues were recovered as NEP A and NEP B, respectively (on a molar basis after correction for the different molecular weights of RA and NEPs).

These findings suggest a rapid enzymatic transformation of RA into NEPs A and B in both field- and in vitro-grown tissues. This is followed by rapid NEP degradation in field-grown tissues, but significantly slower degradation in in vitro cultures. Therefore, NEP degradation in aqueous media is likely also enzyme-catalyzed rather than a consequence of chemical instability. To verify the role of endogenous enzymes, in vitro *M. aquatica* tissues (Sample 1) were incubated in distilled water at room temperature for 15 min, heated to 100 °C for 2 min, and then incubated for an additional 120 min at room temperature. To account for the total 135 min exposure to room-temperature water, the metabolite profile of this sample was compared with that of in vitro *M. aquatica* tissues incubated continuously for 135 min and 15 min (Figure 4 and Figure 5).

A substantial portion of the RA from the untreated control (sample UT, 22.7 mg/g, (RPD = 2.2%) was converted into NEPs A and B within the first 15 min, leaving 5.23 mg/g (RPD = 0.76%) of RA, alongside 2.20 mg/g (RPD = 2.7%) of NEP A and 8.36 mg/g (RPD = 4.3%) of NEP B in the sample DW15 (Figure 5). This metabolite composition remained stable during the subsequent 120-min incubation when heat treatment was applied beforehand: sample DW15-100 °C-DW120 contained 6.45 mg/g (RPD = 5.3%) RA, 2.58 mg/g (RPD = 0.78%) NEP A, and 8.49 mg/g (RPD = 3.8%) NEP B. In contrast, a 135-min incubation without the heating step (sample DW135) resulted in a severe reduction across all compounds, yielding only 0.24 mg/g (RPD = 8.3%) of RA, 0.32 mg/g (RPD = 3.2%) of NEP A, and 3.23 mg/g (RPD = 10.9%) of NEP B. These results confirm that NEPs A and B are chemically stable in aqueous media over the tested duration, and their degradation is driven by endogenous enzymatic activity (Figure 4 and Figure 5).

### 3.3. Enzymatic Conversion of Rosmarinic Acid into Nepetoidins A and B in the Aerial Parts of Melissa officinalis, Origanum vulgare, Satureja hortensis, Thymus serpyllum, and Thymus vulgaris During Aqueous Extraction

Tissues from five additional Nepetoideae species, all medicinal plants known to contain high levels of RA (Supplementary Table S1), namely the leaves of *M. officinalis* (20.6 mg/g RA) and the aerial parts of *O. vulgare* (13.2 mg/g in Sample 1 and 25.1 mg/g in Sample 2), *S. hortensis*, *T. serpyllum*, and *T. vulgaris* (11.7, 13.7, and 13.0 mg/g RA, respectively), also exhibited rapid enzymatic conversion of RA into NEPs A and B (Supplementary Table S2A). This was followed by extensive degradation of the NEPs during incubation of the tissues in an aqueous medium at room temperature. The maximum yields of NEPs A and B were observed after 5 or 15 min of incubation and corresponded to 3–9% (*T. vulgaris* and *O. vulgare*, Sample 1) of the RA content of the respective untreated tissues (on a molar basis, adjusting for the molecular weight differences between RA and the NEPs).

### 3.4. Comparison of Rosmarinic Acid Conversion Efficiency into Nepetoidins A and B Among Different Samples

The conversion patterns of RA in the tissues of the above five field-grown plant species were comparable to those observed in the tissues of the three field-grown *Mentha* species and *S. officinalis* (Sample 1), as the maximum combined levels of NEPs in the water-treated tissues accounted for less than 15% of the RA content of the corresponding untreated samples. In contrast, in tissues from in vitro-grown *Mentha* plants, the maximum combined amount of NEPs formed during water treatment was equivalent to more than 60% of the RA content of the corresponding untreated tissues.

Similar differences were observed in RA conversion between two *S. officinalis* leaf tissue samples, both derived from field-grown plants. Sample 1 was collected from a home-grown plant, whereas Sample 2 was obtained commercially as dried tea. As described above (Section 3.1), Sample 1 exhibited a maximum increase in the combined concentration of NEPs A and B of 2.12 mg/g after 5 min of water incubation, corresponding to less than 10% of the RA content present in the untreated tissue (27.4 mg/g, RPD = 1.8%) (Figure 3, Supplementary Table S2A). In contrast, in Sample 2, the maximum combined concentration of NEPs A and B reached 4.83 mg/g after 30 min of water incubation, corresponding to approximately 40% of the RA content of the untreated tissue (14.9 mg/g, RPD = 13.4%) (on a molar basis after correction for the different molecular weights of RA and NEPs).

These results indicate that the rapid decomposition of RA, accompanied by the formation of NEPs A and B and their subsequent degradation, is a common enzyme-catalyzed process during the aqueous incubation of pulverized tissues across the nine medicinal plant species examined, representing six genera within the Nepetoideae subfamily.

Based on the structural relationship between RA, NEP A, and NEP B (Figure 1), the endogenous enzymes active in an aqueous medium could theoretically convert RA into NEP B via an oxidative decarboxylation process. Based on established oxidative decarboxylation mechanisms of plant metabolites [44], we propose that the reaction is initiated by hydride transfer from the Cα atom of the 3-(3,4-dihydroxyphenyl)-2-hydroxypropanoic acid moiety of RA, generating a quinoid intermediate. The resulting p-quinone methide acts as an electron sink, promoting Cβ decarboxylation and leading to the formation of the corresponding styryl-dihydroxyphenyl derivative. The formation of NEP A would additionally require enzymatic isomerization of the 3′,4′-ortho-dihydroxy substitution pattern of NEP B to the corresponding 3′,5′-meta-dihydroxy arrangement (Figure 1). Alternatively, analogous to the formation of NEP B from RA, NEP A may also originate from a precursor compound; however, such a precursor has not yet been identified. Since we could not find examples in the literature of enzymes catalyzing the conversion of a 3′,4′-ortho-dihydroxy substitution pattern into a 3′,5′-meta-dihydroxy arrangement, the precursor hypothesis appears to be a more plausible explanation. Although the levels of NEPs A and B decreased during prolonged incubations, no corresponding degradation products were detected by HPLC-UV-MS analysis.

However, substantial differences in the efficiency of RA conversion to NEPs were observed both between field-grown tissues of the same species (i.e., the two *S. officinalis* samples) and between field-grown and in vitro-grown tissues of *Mentha* species. Thus, conversion efficiency was influenced not only by the origin of the plant material (field-grown versus in vitro-grown) but also by sample-specific factors, leading to considerable variation in the maximum NEP yields attained during incubation. We hypothesize that the generally lower accumulation of NEPs during aqueous incubation in field-grown plants, compared with in vitro-grown plants, is attributable to higher activity of NEP-degrading enzyme(s) in field-grown tissues. This enhanced enzymatic activity may reflect stress responses induced by environmental factors, such as interactions with pathogenic or symbiotic bacteria and fungi, which are typically more prevalent in the natural habitats of field-grown plants than under in vitro culture conditions. Moreover, because these environmental factors can vary among habitats, they may also contribute to the observed differences in enzyme activity among field-grown samples.

### 3.5. Practical Implications of Rosmarinic Acid Conversion

For the isolation of NEPs A and B, plant materials that exhibited relatively high levels of these compounds following aqueous incubation were considered the most promising sources. These included the aerial parts of three in vitro-grown *Mentha* species, *M. aquatica* (Sample 2), *M. × piperita*, and *M. spicata*, incubated in water at room temperature for 15 min, as well as *M. aquatica* (Sample 1) and *S. officinalis* (Sample 2, obtained commercially as dried tea), incubated under the same conditions for 30 min (Supplementary Table S2A,B). However, due to the limited availability of biomass from the in vitro-grown *Mentha* species, *S. officinalis* (Sample 2) was selected for preparative isolation. According to HPLC-UV quantification, *S. officinalis* (Sample 2) contained 0.90 mg/g NEP A and 3.93 mg/g NEP B after 30 min of aqueous incubation. Using this incubation period for preparative-scale isolation, NEPs A and B were obtained in yields of 0.012% and 0.067%, respectively (on a dry-weight basis), through a simple one-step preparative HPLC procedure. Notably, these yields exceed the highest values previously reported in the literature by more than 50-fold for NEP A and 15-fold for NEP B (Supplementary Table S1). The isolated compounds were obtained in sufficient amounts for structural confirmation by NMR spectroscopy and for subsequent in vitro bioactivity assays, as detailed in Section 3.8 and Section 3.9.

### 3.6. Rosmarinic Acid and Related Phenylpropanoids in Salvia nemorosa, S. pratensis, S. sclarea, and S. verticillata: Identification and Conversions in Aqueous Medium

Although *S. officinalis* has been extensively characterized phytochemically, considerably less is known about the specialized metabolites of its related species, *S. nemorosa*, *S. pratensis*, *S. sclarea*, and *S. verticillata*. Given their well-developed root systems, roots and aerial parts (or leaves) have been analyzed separately, revealing substantial levels of RA in both belowground and aboveground tissues (Supplementary Table S1). In addition to the widespread occurrence of RA, its glucoside has previously been reported in the aerial parts and roots of *S. pratensis* and in the aerial parts of *S. verticillata* (Supplementary Table S1). Our HPLC-UV-HR-MS analysis confirmed these literature findings and revealed high concentrations of RA in both roots and aerial parts of the investigated *Salvia* species. In the roots of *S. nemorosa*, *S. pratensis*, *S. sclarea*, and *S. verticillata*, RA contents ranged from 10.8–18.2, 16.2–24.0, 3.21–4.15, and 27.7–55.5 mg/g, respectively. Corresponding concentrations in the aerial parts were 19.3–21.3, 18.6–23.4, 17.1–20.1, and 13.0–59.1 mg/g, respectively (all related measurements, including replicate measurements, are provided in Supplementary Table S2C–F). These results confirm that both organs of these four *Salvia* species represent rich sources of RA. Compared to RA, its glucoside was a minor constituent, detected predominantly in the roots of the studied species. Its concentration ranged from 1.13 mg/g in *S. nemorosa* (Sample 2) to 3.10 mg/g in *S. pratensis* (Sample 1). In contrast, only trace levels were found in *S. sclarea* (0.05–0.08 mg/g). The roots of *S. verticillata* exhibited substantial variability, with concentrations ranging from 8.22 mg/g (Sample 3) to trace amounts below the limit of quantification (Sample 6) (Supplementary Table S2C–F). For quantification purposes, a calibration curve for RA glucoside was constructed using the compound isolated from its richest identified source, *S. verticillata* (Sample 3) (Supplementary Figures S3 and S4). The identity of the isolated compound was subsequently confirmed by NMR spectroscopy (Section 3.8).

As with the field-grown plant tissues described above, incubation of root and aerial tissues of *S. nemorosa*, *S. pratensis*, *S. sclarea*, and *S. verticillata* in water at room temperature led to rapid decomposition of RA. RA glucoside also underwent decomposition under the same conditions. Concurrently with the decline of these compounds, the concentrations of NEPs A and B increased, reaching maximum levels after 5 min of incubation. Prolonged incubation subsequently led to extensive degradation of both NEPs. In the aerial tissues, the combined maximum yields of NEPs A and B corresponded to 1.5–15.2% of the initial RA content on a molar basis, with the lowest and highest conversions observed in *S. pratensis* Sample 1 and *S. verticillata* Sample 5, respectively. In the root tissues, the corresponding values ranged from 2.2% (*S. verticillata* Sample 5) to 9.2% (*S. nemorosa* Sample 3) (Supplementary Table S2C–F).

**Figure 6:**
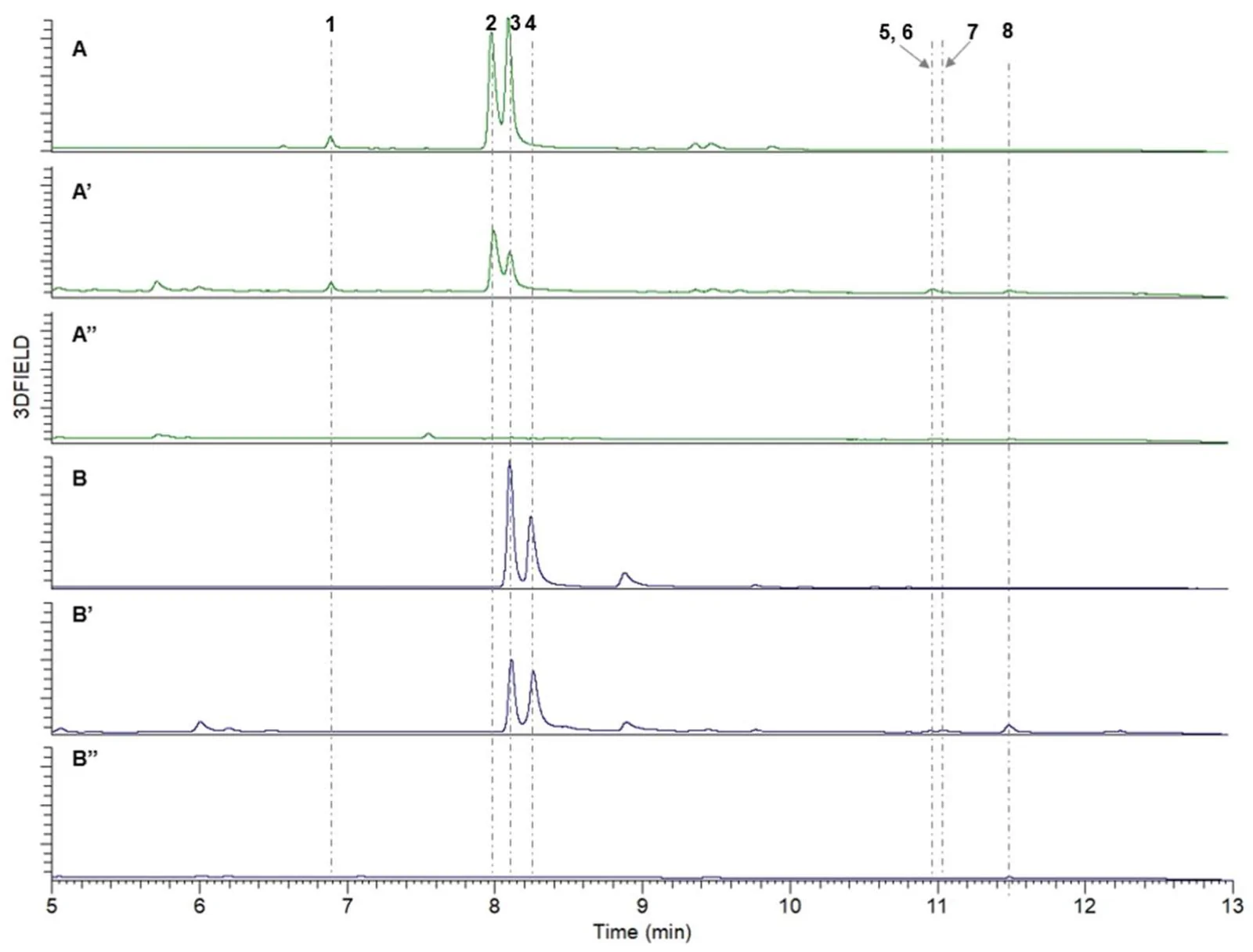
HPLC–UV chromatograms of extracts prepared from the roots of *Salvia pratensis* (A, A′, A″) and *Salvia verticillata* (B, B′, B″) using different extraction procedures. Pulverized tissues were extracted directly with 80% MeOH (A and B). Alternatively, pulverized tissues were suspended in distilled water and incubated at room temperature for 5 min (A′ and B′) or 45 min (A″ and B″). Following aqueous incubation, absolute methanol was added to obtain a final methanol concentration of 80%. Chromatograms A, A′, and A″ correspond to samples SP/R/1/UT, SP/R/1/DW5, and SP/R/1/DW45, respectively, whereas chromatograms B, B′, and B″ correspond to samples SV/R/8/UT, SV/R/8/DW5, and SV/R/8/DW45, respectively, in Supplementary Table S2C,D, where the quantitative phenylpropanoid compositions of these samples are provided. For improved visualization of peak profiles, the chromatograms were displayed using signal intensity scales of 2×, 4×, 4×, 1×, 2×, and 2× for A, A′, A″, B, B′, and B″, respectively. Compound numbers correspond to rosmarinic acid-3-*O*-β-D-glucoside (**1**), salvianolic acid K (**2**), rosmarinic acid (**3**), salvianolic acid O (**4**), nepetoidin A (**5**), salvianolic acid P (**6**), salvianolic acid Q (**7**), and nepetoidin B (**8**).

Comparison of the chromatograms obtained from untreated root extracts of *S. pratensis* and *S. verticillata* with those of the corresponding tissues incubated in water at room temperature revealed, in addition to the extensive decomposition of RA and RA glucoside, the marked decomposition of two other compounds (Figure 6). In *S. pratensis*, a compound eluting immediately before RA (compound **2**) showed substantial degradation, whereas in *S. verticillata*, a compound eluting immediately after RA (compound **4**) exhibited a similar behavior (Figure 6).

**Figure 7:**
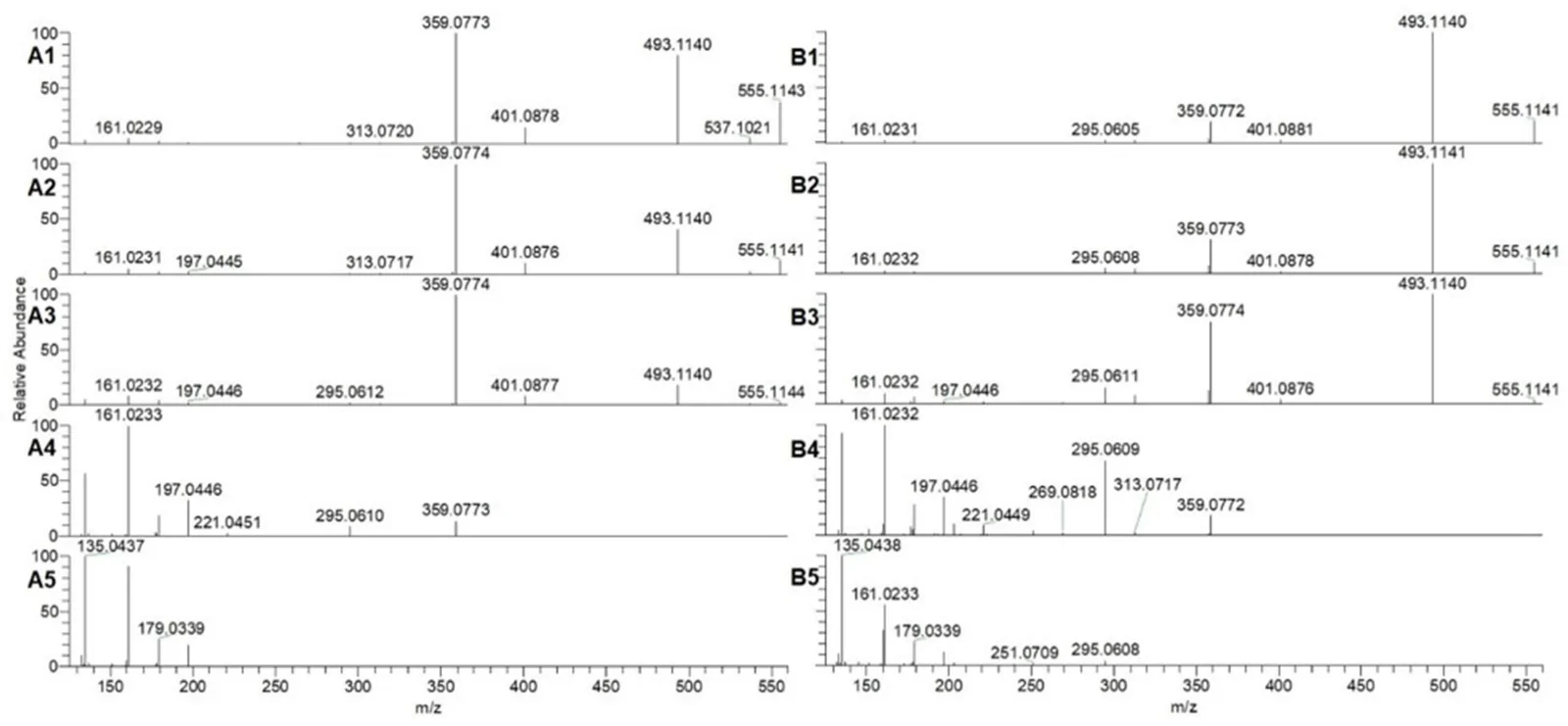
HR-MS/MS spectra of salvianolic acid K (A1–A5) and salvianolic acid O (B1–B5) acquired from their deprotonated molecular ions ([M − H]^−^, *m*/*z* 555) in negative ionization mode at collision-induced dissociation energies of 10 (A1, B1), 12 (A2, B2), 15 (A3, B3), 30 (A4, B4), and 45 eV (A5, B5).

**Figure 8:**
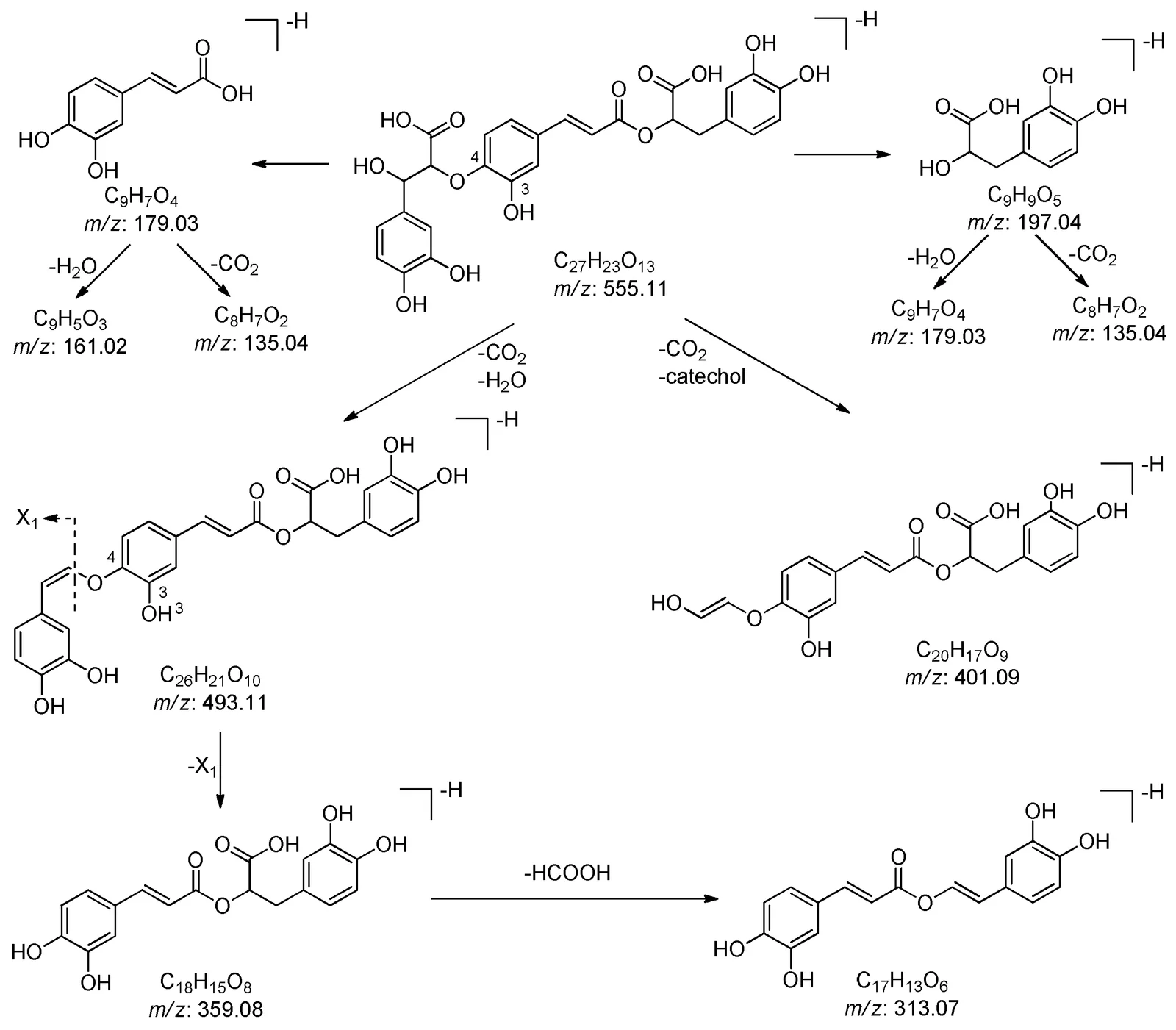
Proposed fragmentation pathways of the deprotonated salvianolic acid K, illustrating the structures of the characteristic fragment ions observed in the HR-MS/MS spectra of this compound recorded in negative ionization mode at collision-induced dissociation energies between 10 and 45 eV (Figure 7).

The UV spectra of compounds **2** and **4** were highly similar to that of RA. Furthermore, HR-MS analysis indicated the same molecular formula, C_27_H_24_O_13_, for both compounds (Table 1, Supplementary Figures S1 and S2). Based on these spectroscopic characteristics and literature data, compound **2** from the roots of *S. pratensis* was tentatively assigned as the trimeric phenylpropanoid SAA K, previously reported in the aerial parts of this species (Supplementary Table S1). Following its isolation from untreated roots of *S. pratensis* Sample 1 (Supplementary Figure S4), NMR spectroscopic analyses unequivocally confirmed the identity of compound **2** as SAA K (see Section 3.8). NMR analysis of compound **4**, isolated from the roots of *S. verticillata* Sample 3 (Supplementary Figure S4), established its structure as a positional isomer of SAA K. As this compound has not previously been reported from a natural source, it is herein designated as SAA O. Consistent with the positional isomeric relationship between SAAs K and O, the HR-MS/MS spectra acquired from their identical deprotonated molecular ions ([M − H]^−^, *m*/*z* 555) exhibited the same fragment ion species (Figure 7 and Figure 8). Nevertheless, marked differences in the relative abundances of several diagnostic fragment ions enabled the reliable differentiation of the two isomers, as discussed in detail below (Section 3.7).

Calibration curves were generated using the isolated SAAs K and O, enabling quantification of both compounds in the plant tissues (Supplementary Figure S3). Comparative analysis of the metabolite profiles of untreated intact root and aerial tissues of *S. nemorosa*, *S. pratensis*, *S. sclarea*, and *S. verticillata* revealed remarkably high concentrations of SAA K in the roots of *S. nemorosa*, *S. pratensis*, and *S. sclarea*, ranging from 14.3–17.5, 40.0–43.2, and 5.19–6.37 mg/g, respectively (Supplementary Table S2D–F). In contrast, SAA O accumulated predominantly in the roots of *S. verticillata*, reaching concentrations of 20.7–78.6 mg/g (Supplementary Table S2C).

In addition to RA, SAAs K and O were the major phenylpropanoid compounds of the roots in these four *Salvia* species. The high concentrations of SAAs K and O in the roots, together with their substantially lower levels in the aerial tissues, indicate that these compounds are root-specific metabolites in the four *Salvia* species examined.

Incubation of the root and aerial tissues of *S. nemorosa*, *S. pratensis*, *S. sclarea*, and *S. verticillata* in water at room temperature resulted not only in the decomposition of RA and its glucoside, as discussed above, but also in the rapid decomposition of SAAs K and O. The decomposition of SAA K in *S. nemorosa*, *S. pratensis*, and *S. sclarea* tissues was accompanied by the formation of compound **6**, whereas the decomposition of SAA O in *S. verticillata* tissues led to the formation of compound **7** (Figure 6, Supplementary Table S2C–F). Based on HPLC-HR-MS analyses, compounds **6** and **7** were found to share the identical molecular formula C_26_H_22_O_11_ (Table 1, Supplementary Figure S2). Comparison of this molecular formula with that of SAAs K and O (C_27_H_24_O_13_) indicates the formal loss of a formic acid moiety during the conversion of both SAAs, yielding compounds **6** and **7**. Since SAAs K and O both contain an RA structural unit, and the conversion of RA via oxidative decarboxylation at the C8′ position was demonstrated above, a similar elimination of the C8′ carboxyl group in SAAs K and O is highly plausible (Figure 1). On this basis, the structures proposed for compounds **6** and **7** are shown in Figure 1.

Among compounds **6** and **7**, compound **6** could be isolated in pure form from selected plant tissues containing relatively high levels of this metabolite following optimized aqueous incubation, as detailed below) (Supplementary Figure S4). The isolated compound enabled the establishment of a calibration curve for its quantification in various plant tissues (Supplementary Figure S3), while NMR analysis confirmed its proposed structure as a previously undescribed natural product, designated as SAA P (see Section 3.8). In contrast, compound **7** could not be obtained in pure form. Its structural assignment was therefore based on its presumed formation from SAA O, as discussed above, together with its HR-MS/MS fragmentation characteristics (see Section 3.7). These results tentatively confirmed that compound **7** is a previously undescribed natural product, which was designated as SAA Q.

Untreated intact tissues containing SAA K, namely the roots of *S. nemorosa*, *S. pratensis*, and *S. sclarea*, contained only negligible amounts of SAA P (up to 0.05 mg/g), and the aerial tissues of these species contained either no detectable (*S. pratensis*) or only trace amounts of this compound (Supplementary Table S2D–F). Likewise, untreated intact tissues of *S. verticillata*, which contained SAA O, also accumulated SAA Q, although only at trace levels (Supplementary Table S2C).

Incubation of tissues in water at room temperature resulted in the rapid decomposition of SAA K in *S. nemorosa*, *S. pratensis*, and *S. sclarea*, accompanied by the formation of SAA P (Figure 6, Supplementary Table S2D–F). The concentration of SAA P reached its maximum after 5 min of incubation in the root tissues, attaining 0.55 mg/g in *S. sclarea* (Sample 2), 0.45 mg/g in *S. nemorosa* (Sample 3), and 0.42 mg/g in *S. pratensis* (Sample 1). Prolonged incubation led to a gradual decline in SAA P levels. A similar conversion was observed in *S. verticillata*, where the rapid decomposition of SAA O was accompanied by the formation of SAA Q (Figure 6, Supplementary Table S2C). The latter reached its highest concentration after 5 min of incubation in root tissues (0.42 mg/g; Sample 8, named SV/R/8/DW5 in Supplementary Table S2C), followed by a decrease during longer incubation periods (Figure 6).

Because the highest concentration of SAA P (0.55 mg/g) was detected in the roots of *S. sclarea* after 5 min of water incubation, this tissue was selected for the isolation of the compound (Supplementary Table S2F, Sample 2, named SS/R/2/DW5). Although SAA Q also accumulated to a relatively high level in the roots of *S. verticillata* after 5 min of incubation (0.42 mg/g), it could not be isolated in pure form because co-eluting impurities could not be separated by preparative HPLC. Owing to the limited availability of SAA Q, its concentrations in tissue samples were estimated using the calibration curve established for SAA P.

Evidence for the enzymatic nature of the decomposition of SAAs K and O, as well as that of RA and its glucoside, was obtained using root tissues of *S. pratensis* (Sample 1) and *S. verticillata* (Sample 9). When the tissues were heated at 100 °C for 2 min prior to incubation in water at room temperature for 45 min, the concentrations of these compounds remained comparable to those measured in the untreated samples (all related measurements, including replicate measurements, are provided in Supplementary Table S2C,D). This observation indicates that thermal inactivation of the enzyme(s) responsible for their conversion effectively prevented their decomposition. However, whether the decomposition of SAAs P and Q, formed enzymatically from SAAs K and O, respectively, is also enzyme-catalyzed was not investigated in the present study.

To evaluate the conversion efficiency, the initial SAAs K and O contents in untreated root tissues of *S. sclarea* (6.37 mg/g, RPD = 21.5% in Sample 2, named SS/R/2/UT in Supplementary Table S2F) and *S. verticillata* (78.6 mg/g, RPD = 4.0% in Sample 8, named SV/R/8/UT in Supplementary Table S2C) were compared with the maximum levels of SAAs P and Q detected after 5 min of incubation (0.55 mg/g and 0.42 mg/g, respectively, in SS/R/2/DW5 and SV/R/8/DW5 samples). Based on a molar conversion that accounts for the differences in molecular weights, approximately 10% of SAA K was recovered as SAA P, whereas only 0.6% of SAA O was recovered as SAA Q. This markedly lower recovery of SAA Q compared to SAA P (0.6% vs. 10%) suggests that SAA Q undergoes a substantially higher extent of decomposition during aqueous incubation.

### 3.7. HPLC-HR-MS/MS-Based Mass Fragmentation Study of Nepetoidins A and B and Salvianolic Acids K, O, P, and Q

To differentiate structurally related phenylpropanoids sharing identical molecular formulas, namely NEPs A and B (C_17_H_14_O_6_), SAAs K and O (C_27_H_24_O_13_), and SAAs P and Q (C_26_H_22_O_11_), their deprotonated molecular ions were subjected to fragmentation in negative ionization mode using collision-induced dissociation (CID) energies ranging from 10 to 45 eV. The MS/MS spectra of NEPs A and B, generated from the precursor ion at *m*/*z* 313, exhibited identical fragment ions. These included an ion at *m*/*z* 161.02 (C_9_H_5_O_3_), corresponding to a dehydrated caffeoyl moiety (derived from *m*/*z* 179.03, C_9_H_7_O_4_), alongside a deprotonated hydroxyvinyl catechol moiety at *m*/*z* 151.04 (C_8_H_7_O_3_) and its dehydrated fragment at *m*/*z* 133.03 (C_8_H_5_O_2_). Because the MS/MS spectra of both NEPs contained the same product ions with comparable relative intensities, the two isomers could not be differentiated by HPLC-HR-MS/MS (Supplementary Figure S5).

**Figure 9:**
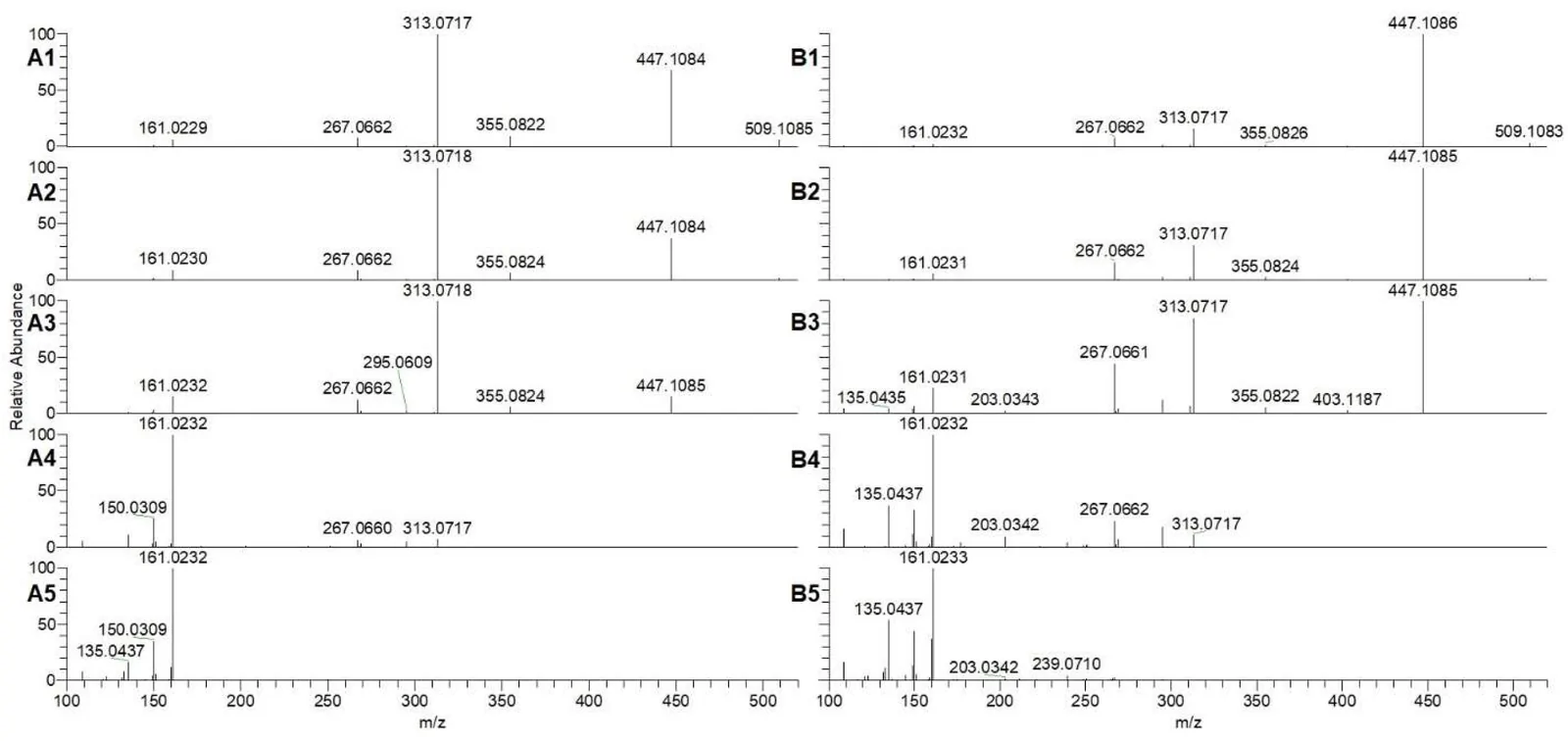
HR-MS/MS spectra of salvianolic acid P (A1–A5) and salvianolic acid Q (B1–B5) acquired from their deprotonated molecular ions ([M − H]^−^, *m*/*z* 509) in negative ionization mode at collision-induced dissociation energies of 10 (A1, B1), 12 (A2, B2), 15 (A3, B3), 30 (A4, B4), and 45 eV (A5, B5).

**Figure 10:**
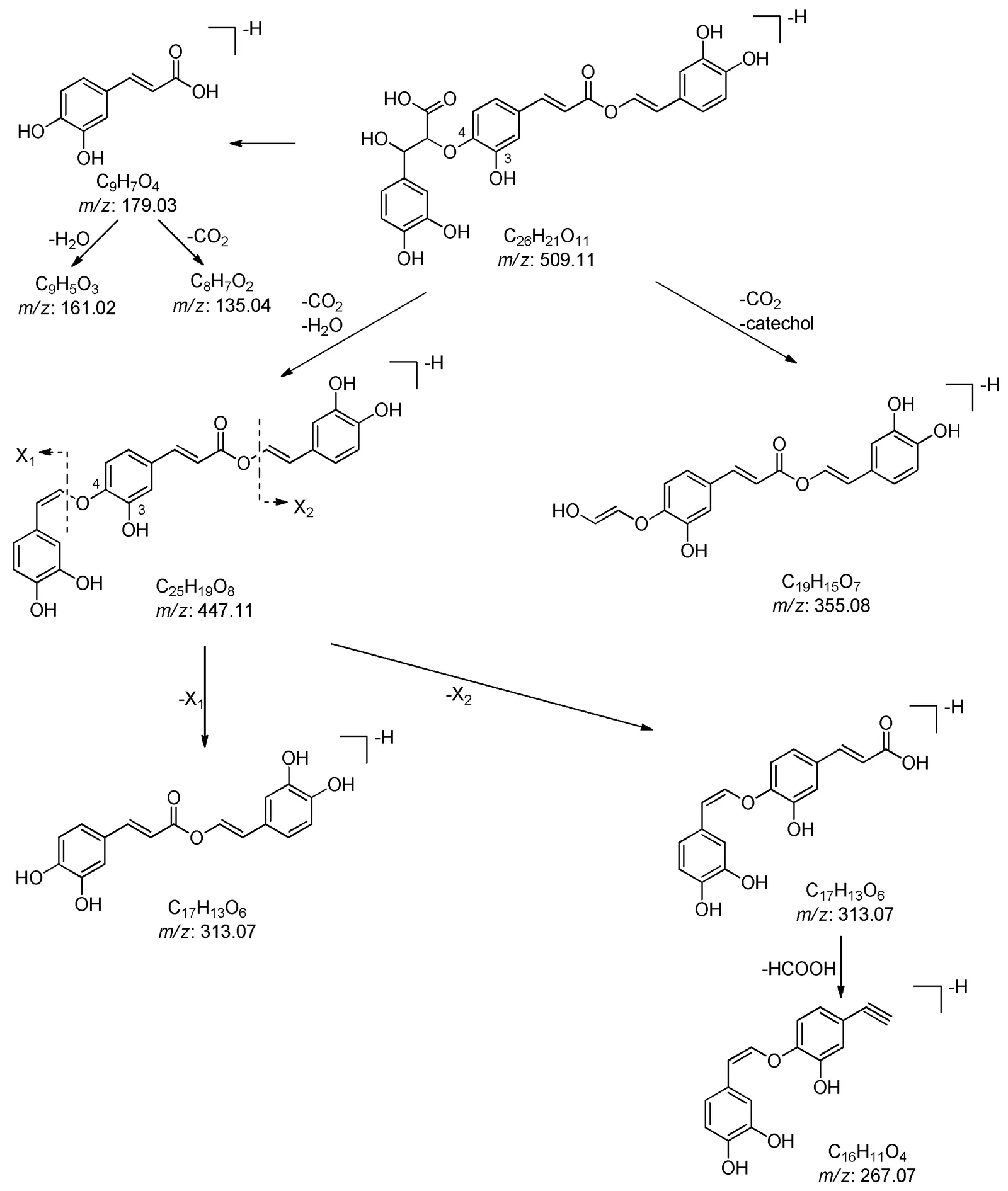
Proposed fragmentation pathways of the deprotonated salvianolic acid P, illustrating the structures of the characteristic fragment ions observed in the HR-MS/MS spectra of this compound recorded in negative ionization mode at collision-induced dissociation energies between 10 and 45 eV (Figure 9).

The four structurally related trimeric phenylpropanoids, SAAs K, O, P, and Q, exhibit highly similar fragmentation pathways (Figure 7, Figure 8, Figure 9 and Figure 10). As an initial fragmentation step, all four compounds undergo the sequential loss of H_2_O and CO_2_ from their common 3-(3,4-dihydroxyphenyl)-2,3-dihydroxypropanoic acid moiety, yielding fragment ions containing a vinylcatechol unit (X_1_) (Figure 8 and Figure 10). These ions were observed at *m*/*z* 493.11 (C_26_H_21_O_10_) for SAAs K and O and at *m*/*z* 447.11 (C_25_H_19_O_8_) for SAAs P and Q. Subsequent elimination of the vinylcatechol (X_1_) unit from these precursor fragments generated ions at *m*/*z* 359.08 (C_18_H_15_O_8_) and *m*/*z* 313.07 (C_17_H_13_O_6_), respectively (Figure 7, Figure 8, Figure 9 and Figure 10).

The relative intensity ratios of the fragment ion pairs *m*/*z* 493.11/359.08 and *m*/*z* 447.11/313.07 provide a reliable means for distinguishing the respective isomeric pairs, SAAs K and O, and SAAs P and Q. In the MS/MS spectra of SAA K, the relative intensity of the ion at *m*/*z* 493.11 was consistently lower than that of the ion at *m*/*z* 359.08 at CID energies of 10, 12, and 15 eV (Figure 7). In contrast, SAA O exhibited the opposite trend, with the ion at *m*/*z* 493.11 being more abundant than the ion at *m*/*z* 359.08 (Figure 7). Consequently, the intensity ratio I_493.11_/I_359.08_ was consistently <1 for SAA K and >1 for SAA O. For example, at a CID energy of 12 eV, this ratio was 0.45 for SAA K (45%/100%) and 3.3 for SAA O (100%/30%). This collision energy, therefore, provides effective differentiation of the isomers, yielding a significant difference between their I_493.11_/I_359.08_ ratios (0.5 for SAA K versus 2.5 for SAA O).

A similar relationship was observed for SAAs P and Q. The intensity ratio I_447.11_/I_313.07_ was consistently <1 for SAA P and >1 for SAA Q at CID energies of 10, 12, and 15 eV, providing a diagnostic criterion for differentiating these isomeric compounds (Figure 9).

Considering that the loss of the vinylcatechol unit (X_1_) from the *m*/*z* 493.11 ions of both SAAs K and O generated the common fragment ion at *m*/*z* 359.08, and that the I_493.11_/I_359.08_ ratio was higher for SAA O (>1) than for SAA K (<1), the *m*/*z* 493.11 ion of SAA O appears to be more stable toward CID-induced fragmentation than the corresponding ion of SAA K (Figure 7 and Figure 8). This difference may be attributed to the attachment of the vinylcatechol unit at the C3 position in SAA O, whereas in SAA K the same moiety is attached at the C4 position (Figure 1).

Similarly, elimination of the vinylcatechol unit from the *m*/*z* 447.11 ions of both SAAs P and Q generated the common fragment ion at *m*/*z* 313.07 (Figure 9 and Figure 10). The higher intensity ratio I_447.11_/I_313.07_ observed for SAA Q (>1) compared with SAA P (<1) suggests that the *m*/*z* 447.11 ion of SAA Q, containing the vinylcatechol unit at the C3 position, is more stable during CID-induced fragmentation than the corresponding ion of SAA P, in which the vinylcatechol unit is attached at the C4 position.

These observations suggest that the vinylcatechol unit attached at the C4 position undergoes cleavage more readily than that attached at the C3 position during CID-induced fragmentation of SAAs K, O, P, and Q, resulting in isomer-specific differences in the relative intensities of the corresponding fragment ions.

In addition to the diagnostic fragment ions discussed above, several other characteristic fragment ions were observed in the MS/MS spectra of SAAs K, O, P, and Q at CID energies ranging from 10 to 45 eV. Although these ions did not exhibit pronounced isomer specificity, they provided additional evidence supporting the proposed structures of the compounds (Figure 7, Figure 8, Figure 9 and Figure 10).

### 3.8. Structural Elucidation by NMR Spectroscopy

Structural characterization was based on 1D (^1^H, ^13^C) and 2D (^1^H–^1^H COSY, ^1^H–^1^H NOESY, ^1^H–^13^C HSQC, and ^1^H–^13^C HMBC) experiments (Table 2 and Table 3; Supplementary Figures S6–S42).

The ^1^H-NMR spectrum of compound **4** (C_27_H_24_O_13_) was initially recorded in DMSO-*d*_6_, and fourteen carbon-bound protons were noticed, while two additional proton signals appeared (4.65 br s, 7.03 br s) upon addition of a few µL of D_2_O to the NMR sample (Table 2). The remaining eight exchangeable protons (hydroxyl and carboxyl OHs) completely disappeared due to the presence of D_2_O, thereby simplifying the ^1^H NMR spectrum. As a starting point in the NMR-based structure elucidation nine aromatic proton signals at *δ*_H_ 7.03 (1H, br s, H-2), 6.66 (1H, m (ov), H-2′), 6.88 (1H, d, *J* = 2 Hz, H-2″), 6.8 (1H, d, *J* = 8.1 Hz, H-5), 6.64 (1H, d, *J* = 8.0 Hz, H-5′), 6.67 (1H, m (ov), H-5″), 7.15 (1H, dd, *J* = 8.4, 2.0 Hz, H-6), 6.53 (1H, dd, *J* = 8.1, 2.1 Hz, H-6′), 6.73 (1H, dd, *J* = 8.2, 2.0 Hz, H-6″), corresponding to three ABX systems were indicative of 3 distinct 1,2,4-trisubstituted benzene units. The ^1^H-NMR spectrum also revealed two doublets in the aromatic region at *δ*_H_ 7.43 (1H, d, *J* = 15.8 Hz, H-7) and 6.26 (1H, d, *J* = 16 Hz, H-8), indicating a C=C moiety with trans-oriented protons. In the aliphatic region a methylene unit with protons at *δ*_H_ 2.90 (1H, dd, *J* = 14.4, 8.5 Hz, H-7′) and 2.98 (1H, dd, *J* = 14.4, 4.4 Hz, H-7′) was coupled to an oxymethine proton *δ*_H_ 5.03 (1H, dd, *J* = 8.4, 4.5 Hz, H-8′), which indicates a CH_2_-CH(OH) unit. Moreover, two neighboring oxymethine protons were noticed in a low field region *δ*_H_ 4.83 (1H, d, *J* = 5.8 Hz, H-7″) and 4.65 (1H, br s, H-8″), corresponding to a CH(OH)-CH(OH) unit. The ^13^C NMR spectrum and HSQC/HMBC recorded at 328 K (signals shown with *) also confirmed the presence of one methylene group *δ*c (36.3), three oxymethine moieties *δ*c (73.13, 73.26, *83.2), in addition to three carbonyl carbons *δ*c (166.3, 171.16, *171.49). The presence of 1,2,4-trisubstituted benzene units was further confirmed based on nine aromatic C-H pairs *δ*c (115.1–125.25), three quaternary carbons *δ*c (125.52, 127.6, 132.3), and six additional quaternary oxygen-bound carbons *δ*c (144.13–*151.1). Moreover, two olefinic carbons *δ*c (114.1, 145.95) were also noticed. Based on ^1^H–^13^C HMBC correlations (Supplementary Figure S6), one ABX system was connected with the CH_2_-CH(OR)-COOH unit, thus constituting a 3,4-dihydroxyphenyllactic acid subunit. The second ABX system was linked to a CH=CH-COOR moiety, indicating a caffeic acid residue. The third ABX system was coupled to a CH(OH)-CH(OR)-COOH unit, thus forming (3,4-dihydroxyphenyl)glyceric acid moiety.

The HMBC correlation of H-8′ (*δ*_H_ 5.03 ppm) with C-9 (*δ*_C_ 166.3 ppm) confirmed the esterification between the α-hydroxyl of 3,4-dihydroxyphenyllactic acid and the carbonyl group of caffeic acid subunits. Subsequently, the ^1^H–^1^H NOESY spectrum showed a cross-peak between H-8″ (*δ*_H_ 4.65 ppm; α-glyceric acid proton) and H-2 (*δ*_H_ 7.03 ppm; aromatic proton of caffeic acid) (Supplementary Figure S28). Based on the proximity of the C-3 hydroxyl to the H-2 proton, we can conclude that the C-3 position of caffeic acid is connected with the (3,4-dihydroxyphenyl)glyceric acid moiety. To the best of our knowledge, this structural feature has not been reported in the literature; therefore, compound **4** represents a novel entity, designated as SAA O (Table 2, Supplementary Figures S24–S31).

The NMR analysis of compound **2** also confirmed the presence of an esterified caffeic acid through its carboxylate function. It can also be confirmed that 3,4-dihydroxyphenyllactic acid is linked to the caffeic acid moiety, identical to that of SAA O. However, the connection of (3,4-dihydroxyphenyl)glyceric acid moiety to the caffeic acid differed from that of SAA O. In the case of compound **2**, H-8″ (*δ*_H_ 4.49 ppm) exhibited an intense NOESY crosspeak with that of H-5 (*δ*_H_ 6.62 ppm) located on the aromatic ring of caffeic acid (Supplementary Figure S16). This indicative intramolecular proximity between the two protons unequivocally confirmed that the C-4 hydroxyl group of caffeic acid is linked with (3,4-dihydroxyohenyl)glyceric acid moiety. Based on these findings, compound **2** was identified as SAA K, and its NMR data are consistent with those reported in the literature (Table 2, Supplementary Figures S12–S18) [45].

Compounds **1** and **3** were identified as rosmarinic acid-3-*O*-β-D-glucoside [15] and RA [46], respectively, based on the comparison of their NMR spectroscopic data with those found in the literature (Table 2, Supplementary Figures S7–S11 and S19–S23).

The NMR assignments of conversion product **6** closely resembled those of its precursor SAA K (**2**), with the exception of positions 7′ and 8′, where olefinic resonances at *δ*_H_ 5.67 (1 H, d, *J* = 7.2 Hz), and *δ*_H_ 7.15 (1 H, d, *J* = 7.1 Hz) were observed instead of α-methine and β-methylene protons. Compound **6** represents a previously unreported derivative, which was named SAA P (Table 3, Supplementary Figures S34–S40). Compounds **5** and **8** differed from their precursor RA (**3**) by the presence of olefinic bonds at the 7′ and 8′ positions. Compound **8** contained ortho-positioned hydroxyls, whereas in compound **5**, the hydroxyls of ring B were are located at the meta positions. Compounds **5** and **8** were identified as NEP A (*Z*, *E*) [47] (Table 3, Supplementary Figures S32 and S33), and NEP B (*Z*, *E*) [28] (Table 3, Supplementary Figures S41 and S42), respectively.

The absolute configurations of the novel SAAs O and P were not independently established. The NMR experiments were primarily performed to establish the constitution and connectivity of the compounds and to distinguish the positional isomers. In particular, the diagnostic NOESY correlations supported the different attachment positions of the (3,4-dihydroxyphenyl)glyceric acid moiety in SAAs O and K. The stereochemical representation of SAA O was based on its close structural relationship to the known SAA K and comparison of their spectroscopic characteristics, whereas SAA P was identified as an enzymatic conversion product of SAA K and was considered to retain the stereochemical elements unaffected by this transformation. Accordingly, the absolute configurations of the stereogenic centers in SAAs O and P should be regarded as inferred rather than experimentally established in the present study.

### 3.9. In Vitro Antioxidant and Metabolic Inhibitory Activities of the Isolated Compounds

**Figure 11:**
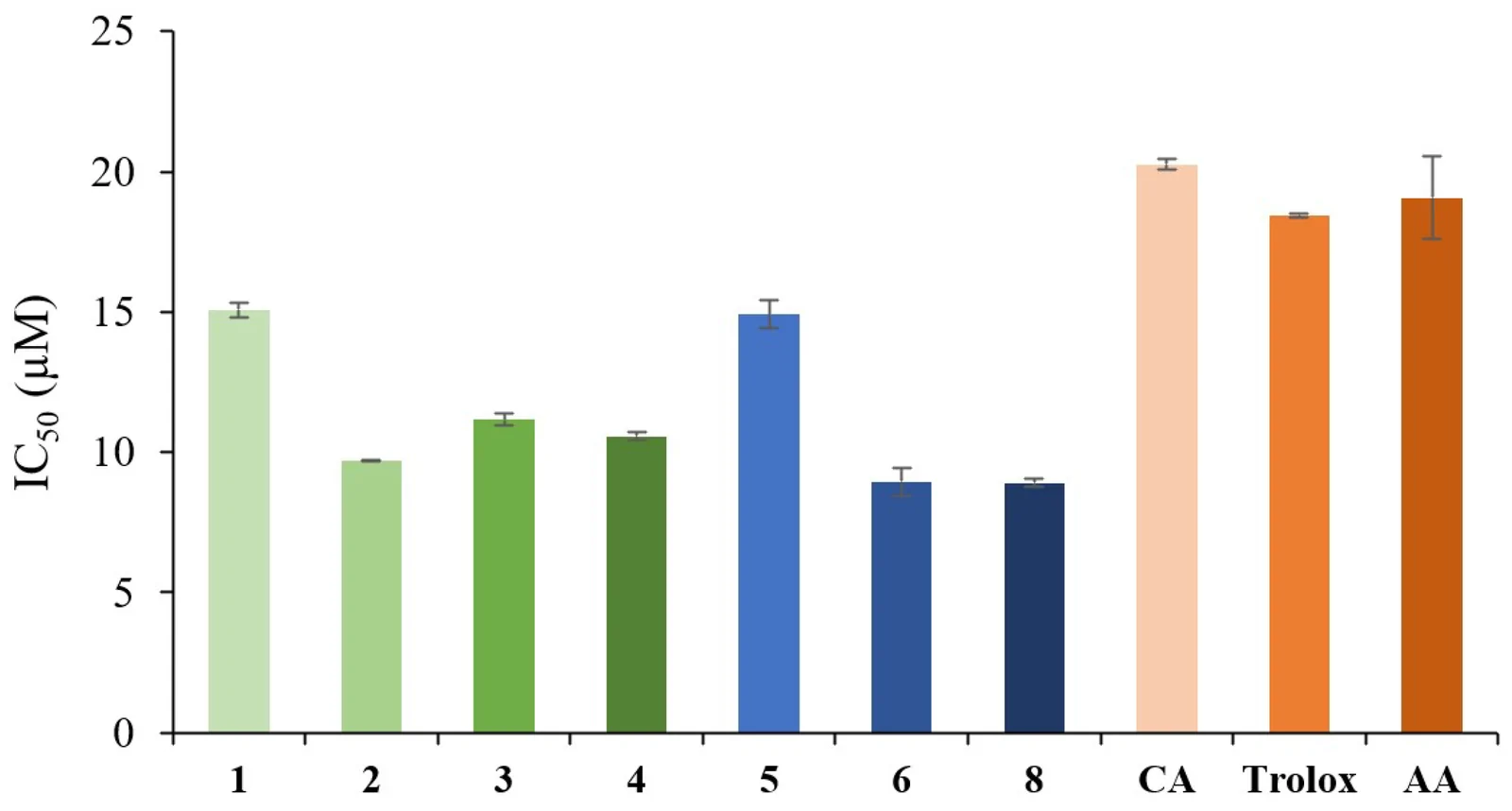
The DPPH radical scavenging activity of rosmarinic acid-3-*O*-β-D-glucoside (**1**), salvianolic acid K (**2**), rosmarinic acid (**3**), salvianolic acid O (**4**), nepetoidin A (**5**), salvianolic acid P (**6**), and nepetoidin B (**8**), compared with standard antioxidants ascorbic acid (AA), chlorogenic acid (CA), and Trolox (values are mean ± SD).

Given that the medicinal relevance of RA is closely associated with its potent radical-scavenging antioxidant activity, the structurally related compounds isolated in the present study were also evaluated for their antioxidant potential [1]. The in vitro antioxidant potential of the isolated compounds was evaluated using the DPPH radical-scavenging assay (Figure 11). Consistent with a previous report [30], RA exhibited stronger DPPH radical-scavenging activity than its glucoside, with IC_50_ values of 11.2 ± 0.2 and 15.1 ± 0.3 µM, respectively (*p* < 0.0001). This observation is consistent with the established structure-activity relationship of phenolic antioxidants, according to which catechol moieties bearing free ortho-dihydroxyl groups are key determinants of DPPH radical-scavenging activity. Consequently, glycosylation of one hydroxyl group of the catechol unit decreases antioxidant potency [48].

SAAs K, O, and P, together with NEP B, exhibited comparable antioxidant activities, with IC_50_ values of 9.6 ± 0.2, 10.6 ± 0.2, 8.9 ± 0.5, and 8.9 ± 0.1 µM, respectively (*p* > 0.05). These values are similar to those of RA (*p* > 0.05) and are consistent with the literature data reported for NEP B [24]. Notably, all four compounds contain two catechol moieties, whereas NEP A, which possesses only one catechol moiety and one resorcinol-type aromatic ring, showed significantly lower activity (IC_50_ = 14.9 ± 0.5 µM; *p* < 0.0001). This comparison further supports the predominant contribution of catechol units to the antioxidant activity of the isolated compounds.

In addition to providing the first experimental confirmation of the antioxidant activities of SAA K and NEP A, this study also demonstrates the antioxidant potential of the newly identified SAAs O and P. Moreover, all isolated compounds exhibited stronger DPPH radical-scavenging activity than the reference antioxidants ascorbic acid (IC_50_ = 19.1 ± 1.5 µM), chlorogenic acid (IC_50_ = 20.3 ± 0.2 µM), and Trolox (IC_50_ = 18.5 ± 0.1 µM), with *p* < 0.0001 for all pairwise comparisons with the reference antioxidants, highlighting their considerable potential as radical scavengers.

In addition to its antioxidant activity, RA has been reported to inhibit various cancer cell lines in vitro [1]. Considering the structural relationship between RA and the related compounds isolated in this work, evaluating their effects on cancer cells is also justified, as this type of bioactivity has not been reported for these compounds. The cytostatic effects of the compounds were assessed in vitro using the Alamar Blue viability assay on five human cancer cell lines: colorectal adenocarcinoma (HT-29), glioblastoma (U87 and U251), lung adenocarcinoma (H838), and monocytic leukemia (MM6). The assay revealed compound-dependent reductions in cellular resazurin-reducing activity across the investigated cell panel. As the assay reflects both cellular metabolic activity and viable-cell number, these results are interpreted as indicative of an apparent cytostatic/metabolic inhibitory effect. Compounds lacking a carboxyl group at the C8′ position, namely SAA P, NEP A, and NEP B, produced greater reductions in the Alamar Blue signal than the more polar carboxylated analogs (Table 4). The higher activity of SAA P, NEP A, and NEP B may be attributed to their greater apolarity relative to RA, RA glucoside, and SAAs K and O, which may facilitate cellular uptake and thereby enhance their cytostatic effects. However, in our next study, we plan to perform additional orthogonal assays, such as apoptosis/necrosis marker analysis and clonogenic assays, to provide evidence for specific cell-death and/or cell-cycle mechanisms, together with selectivity assessment using non-tumorous cell cultures.

## 4. Conclusions

In conclusion, pulverized tissues from 13 medicinally relevant Nepetoideae species (*Melissa officinalis*, *Mentha aquatica*, *M. × piperita*, *M. spicata*, *Origanum vulgare*, *Salvia nemorosa*, *S. pratensis*, *S. sclarea*, *S. officinalis*, *S. verticillata*, *Satureja hortensis*, *Thymus serpyllum*, and *T. vulgaris*) exhibited characteristic compound conversions in aqueous medium, which are most likely attributable to the activity of endogenous enzymes present in these plants. However, because the putative endogenous enzymes responsible for these compound conversions were neither isolated nor investigated using specific enzyme inhibition assays, their direct involvement could not be unequivocally demonstrated. Therefore, alternative explanations for the observed compound conversions cannot be excluded. For example, the decomposition of compounds such as rosmarinic acid and salvianolic acids K and O may also result from chemical reactions with as yet unidentified, heat-labile constituents present in the plant tissues.

The rapid decomposition of rosmarinic acid, accompanied by the formation of nepetoidins A and B and their subsequent degradation, was demonstrated to be a common enzymatic process in these species. In addition, salvianolic acid K, identified for the first time in the roots of *S. nemorosa*, *S. pratensis*, and *S. sclarea*, and salvianolic acid O, identified as a previously undescribed natural compound in the roots of *S. verticillata*, underwent analogous enzymatic conversions in pulverized root tissues, yielding two newly identified compounds, salvianolic acids P and Q, respectively, followed by their subsequent degradation.

These findings indicate that extraction conditions can strongly influence the phytochemical composition and, consequently, the biological relevance of extracts prepared from these plants. Conventional extracts obtained using organic solvents or hot water, conditions that inactivate endogenous enzymes, retain the native metabolite profile, with rosmarinic acid as the major constituent in *Melissa officinalis*, *Mentha aquatica*, *M. × piperita*, *M. spicata*, *O. vulgare*, *Salvia officinalis*, *Satureja hortensis*, *T. serpyllum*, and *T. vulgaris*, and with rosmarinic acid together with salvianolic acid K (*Salvia nemorosa*, *S. pratensis*, and *S. sclarea*) or salvianolic acid O (*S. verticillata*) as the predominant compounds. In contrast, extraction with water at room temperature allows endogenous enzyme activity, resulting in the conversion of rosmarinic acid and salvianolic acids K and O into their corresponding decarboxylated products. These results highlight the importance of the extraction method used for Nepetoideae species, particularly whether they are consumed as cold- or hot-brewed preparations and the extraction time, as these factors can substantially influence the chemical composition and, consequently, the medicinal properties of the resulting preparation.

Because intact tissues contained only trace amounts or no detectable levels of nepetoidins A and B and salvianolic acids P and Q, these compounds were isolated from selected tissues after incubation in water for a defined period that promoted their enzymatic formation from the corresponding precursor compounds. The structures of the isolated compounds were established by extensive NMR and HR-MS/MS analyses. Furthermore, the relative intensity ratios of the fragment-ion pairs *m*/*z* 493.11/359.08 and *m*/*z* 447.11/313.07, obtained by HR-MS/MS at CID energies of 10, 12, and 15 eV, proved to be reliable markers for distinguishing the isomeric pairs salvianolic acids K and O and salvianolic acids P and Q, respectively.

The isolated compounds exhibited structure-dependent antioxidant and metabolic inhibitory activities. The antioxidant activities of salvianolic acids K, O, and P and nepetoidin A were demonstrated experimentally for the first time, whereas the antioxidant activities of rosmarinic acid and its glucoside were consistent with previous literature data. The results confirmed the crucial role of the free hydroxyl groups within the catechol moieties of these compounds in mediating their radical-scavenging activity.

Rosmarinic acid glucoside, salvianolic acids K, O, and P, and nepetoidins A and B were identified for the first time as active compounds against human colorectal adenocarcinoma (HT-29), glioblastoma (U87 and U251), lung adenocarcinoma (H838), and monocytic leukemia (MM6) cell lines. Because Alamar Blue provides a metabolic viability readout, the underlying mechanism of the observed activity requires further investigation using orthogonal assays. Therefore, our results should be interpreted as indicating apparent cytostatic/metabolic inhibitory effects. Comparison of the activities of the compounds revealed that the absence of a carboxyl group at the C8′ position is associated with higher activity.

## Figures and Tables

**Table 3:** ^1^H and ^13^C NMR Data (*δ* in ppm, *J* in Hz) of compounds **5**, **6**, and **8**. Spectra were recorded in DMSO-*d*_6_/D_2_O (**6**) and acetone-*d*_6_ (**5** and **8**) at 500/125 MHz.

No	6		5	8
	*δ*_H_	*δ*_C_	*δ*_H_	*δ*_H_
1	-	129.9	-	-
2	7.14, m (ov)	116.41	7.24, d (2.0)	7.26, d (2)
3	-	150.55/150.61	-	-
4	-	150.61/150.55	-	-
5	6.48, d (8.4)	119.5	6.91, d (8.2)	6.91, d (8.2)
6	7.01, dd (8.4, 2.1)	121.9	7.14, dd (8.2, 2.1)	7.14, dd (8.2, 2.1)
7	7.62, d (15.8)	147.6	7.71, d (15.9)	7.74, d (15.9)
8	6.56, d (15.9)	115.41	6.39, d (16.1)	6.51, d (15.9)
9	-	164.5	-	-
1′	-	126.3	-	-
2′	7.22, d (2.1)	116.94	6.80, s	7.38, d (2.0)
3′	-	145.4	-	-
4′	-	145.4	6.95, s	-
5′	6.73, d (8.1)	116.37	-	6.82, d (8.2)
6′	6.87, m (ov)	122.2	6.80, s	7.02, dd (8.2, 2.1)
7′	5.67, d (7.2)	113.1	6.40, d (12.6)	5.68, d (7.2)
8′	7.15, d (7.1)	132.6	7.84, d (12.8)	7.27, d (7.2)
1″	-	133.2	-	-
2″	6.85, d (2.0)	115.60	-	-
3″	-	144.97	-	-
4″	-	144.93	-	-
5″	6.67, d (8.1)	115.60	-	-
6″	6.70, dd (8.4, 2.0)	119.5	-	-
7″	4.78, d (6.3)	73.7	-	-
8″	4.21, Br (s)	* 86.8	-	-
9″		173.9	-	-

* Signal deduced from HSQC spectrum.

**Table 4:** Effect of rosmarinic acid-3-*O*-β-D-glucoside (**1**), salvianolic acid K (**2**), rosmarinic acid (**3**), salvianolic acid O (**4**), nepetoidin A (**5**), salvianolic acid P (**6**), and nepetoidin B (**8**) on human cancer cell cultures: colorectal adenocarcinoma (HT-29), glioblastoma (U87 and U251), lung adenocarcinoma (H838), and monocytic leukemia (MM6).

Compound	IC_50_ (µM) ± SD				
	MM6	HT-29	U87	U251	H838
**1**	47.6 ± 3.4	>100	>100	>100	60.5 ± 4.4
**2**	21.4 ± 0.6	>100	>100	>100	56.6 ± 3.4
**3**	77.4 ± 4.1	>100	>100	>100	45.6 ± 1.4
**4**	46.3 ± 1.3	>100	>100	>100	77.8 ± 2.4
**5**	24.7 ± 1.7	79.6 ± 3.8	67.2 ± 3.8	88.5 ± 5.4	34.5 ± 0.4
**6**	17.6 ± 0.4	24.7 ± 1.5	34.0 ± 2.4	24.7 ± 0.4	25.6 ± 0.6
**8**	20.0 ± 1.4	19.2 ± 1.4	26.3 ± 1.4	32.5 ± 0.7	22.4 ± 0.4

IC_50_ values (as metabolic activity inhibition) are expressed as mean ± SD from two independent experiments, each performed with four technical replicates. Compounds that did not reduce the Alamar Blue signal by at least 50% within the tested concentration range are reported as IC_50_ > 100 µM. IC_50_ values represent the concentration required to reduce the Alamar Blue fluorescence signal by 50% relative to untreated control cultures. The resulting values were interpreted as an apparent metabolic inhibitory/cytostatic effect.

## Data Availability

The data presented in this study are available within the article or its Supplementary Materials. Additional raw datasets generated during the current study are available from the corresponding authors on reasonable request.

## Supplementary Materials

The following supporting information can be downloaded at: https://www.mdpi.com/article/10.3390/pharmaceutics18081043/s1, Figure S1: UV-spectra; Figure S2A–H: Mass spectra, Figure S3: HPLC-UV calibration curves, Figure S4. HPLC-UV peaks of compounds. Figure S5: HR-MS/MS spectra of nepetoidins A and B at different collision-induced dissociation energies; Figure S6: Key 2D-NMR correlations; Figure S7–S42: NMR spectra of isolated compounds. Table S1: Literature summary; Table S2A–F: Contents of the investigated compounds in selected species.

## Abbreviations

The following abbreviations are used in this manuscript:

RA	Rosmarinic acid
SAA	Salvianolic acid
NEP	Nepetoidin
LOQ	Limit of quantitation
LOD	Limit of detection
RPD	Relative percent difference
ESI	Electrospray ionization
DPPH	2,2-diphenyl-1-picrylhydrazyl
DMEM	Dulbecco’s Modified Eagle’s Medium
FBS	Fetal bovine serum
MM6	MonoMac-6, human monocytic leukemia-derived line with monocyte-like features
HT-29	Human colorectal adenocarcinoma epithelial cell line
H838	Human lung adenocarcinoma, non-small cell lung cancer line
U87	Human glioblastoma/glioma cell line
U251	Human glioblastoma/glioma cell line
SD	Standard deviation
