# Supplementary material for: Aqueous Incubation Reveals Transformation of Rosmarinic Acid in Medicinally Important Nepetoideae Species and Leads to Novel Bioactive Compounds
Source: Pharmaceutics. 2026 Aug 21;18(8):1043. doi: 10.3390/pharmaceutics18081043 (PMC13516284; doi:10.3390/pharmaceutics18081043)
Supplement: Supplementary file 1 [file pharmaceutics-18-01043-s001.zip › pharmaceutics-4457101-supplementary.pdf]

Aqueous Incubation Reveals Transformation of Rosmarinic Acid in Medicinally Important Nepetoideae Species and Leads to Novel Bioactive Compounds

Adila Nazli[1 *], Bernadett Szögi-Tatár[1 *], Szilvia Bősze[2, 3], Gergő Tóth[4, 5], Katalin Solymosi[6], Szabolcs Béni[7 **], Imre Boldizsár[1, 5, 6]**

[1]Department of Pharmacognosy, Semmelweis University, 1085 Budapest, Hungary; nazli.adila@phd.semmelweis.hu (A.N.); szogitatarbernadett@gmail.com (B.S.T.); boldizsar.imre@semmelweis.hu (I.B.)
[2]HUN-REN-ELTE Research Group of Peptide Chemistry, Hungarian Research Network, ELTE Eötvös Loránd University, Pázmány Péter sétány 1/A, 1117 Budapest, Hungary; szilvia.bosze@ttk.elte.hu
[3]Department of Genetics, Cell- and Immunobiology, Semmelweis University, Nagyvárad tér 4, 1089 Budapest, Hungary
[4]Department of Pharmaceutical Chemistry, Semmelweis University, Hőgyes Endre u. 9, 1092 Budapest, Hungary; toth.gergo@semmelweis.hu
[5]Center for Pharmacology and Drug Research & Development, Semmelweis University, 1085 Budapest, Hungary
[6]Department of Plant Anatomy, Institute of Biology, Eötvös Loránd University, Pázmány Péter sétány 1/C, 1117 Budapest, Hungary; katalin.solymosi@ttk.elte.hu (K.S.)
[7]Integrative Health and Environmental Analysis Research Laboratory, Department of Analytical Chemistry, Institute of Chemistry, Eötvös Loránd University, 1117 Budapest, Hungary; szabolcs.beni@ttk.elte.hu
*, ** Equal contributions
**Correspondence: boldizsar.imre@semmelweis.hu (I.B.), szabolcs.beni@ttk.elte.hu (S.B.)

Table S1. Literature summary of the occurrence of rosmarinic acid, rosmarinic acid glucoside, salvianolic acid K, and nepetoidins A and B in Nepetoideae species, indicating the source species, the organs or tissues in which these compounds were detected, and their reported contents expressed as percentages of dry plant material.

Compound	Species	Organ/tissue	Extractant	Amount	References
rosmarinic acid	*Mentha aquatica*	aerial parts	H_2O/MeOH/IPA 80:10:10	2.5	[2]
	Mentha × piperita	aerial parts	H_2O/MeOH/IPA 80:10:10	2.8	[2]
		aerial parts	MeOH	1.2; 2.9	[3]
	Mentha spicata	aerial parts	H_2O/MeOH/IPA 80:10:10	5.8	[2]
		aerial parts	MeOH	0.6; 1.1	[3]
		leaf	EtOH	6.7	[4]
		leaf	30% EtOH	0.7; 1.4	[5]
	Melissa officinalis	aerial parts	H_2O/MeOH/IPA 80:10:10	3.7	[2]
		leaf	30% EtOH	2.7	[5]
	Ocimum basilicum	fresh leaf	EtOAc	≈ 0.003*	[6]
			MeOH	≈ 0.12*	[6]
	Origanum vulgare	aerial parts	H_2O/MeOH/IPA 80:10:10	2.5	[2]
	Salvia nemorosa	leaf	MeOH	0.7; 0.6	[7]
		root	MeOH	0.5; 0.7	[7]
	Salvia officinalis	aerial parts	H_2O/MeOH/IPA 80:10:10	3.9	[2]
		leaf	30% EtOH	0.9; 1.4	[5]
	Salvia pratensis	aerial part	H_2O (100°C)	++	[8]
		root	H_2O (100°C)	+	[8]
		aerial part	MeOH	0.8	[9]
		root	MeOH	1.1	[9]
	Salvia sclarea	aerial part	80% MeOH	2.1; 3.8	[10]
		leaf	MeOH	0.4; 0.5	[7]
		root	MeOH	0.1	[7]
	Salvia verticillata	leaf	MeOH	2.4; 3.7	[11]
		leaf	70% MeOH	8.6	[12]
		aerial part	MeOH	3.0	[13]
		leaf	MeOH	0.8; 2.0	[7]
		root	MeOH	1.9; 4.2	[7]
	Satureja hortensis	aerial parts	H_2O/MeOH/IPA 80:10:10	1.6	[2]
	Thymus vulgaris	aerial parts	H_2O/MeOH/IPA 80:10:10	2.3	[2]
		leaf	30% EtOH	0.5; 0.9	[5]
	Thymus serpyllum	whole plant	75% MeOH	2.2	[14]
rosmarinic acid glucoside	*Perilla frutescens*	seed fluor	70% EtOH	0.035	[15]
	Salvia pratensis	aerial part	H_2O (100°C)	+	[8]
		root	H_2O (100°C)	++	[8]
	Salvia verticillata	aerial part	MeOH	0.047	[13]
	Salvia spp. (10 species)	root	H_2O (80°C)	0.0022; 0.21	[16]
	Thymus serpyllum	whole plant	75% MeOH	0.4	[14]
salvianolic acid K	*Salvia pratensis*	aerial part	H_2O (100°C)	+	[8]
		root	H_2O (100°C)	++	[8]
	Salvia spp. (10 species)	root	H_2O (80°C)	0.00015; 0.6	[16]
	Thymus pseudolanuginosus	aerial part	H_2O (100°C)	0.18	[17]
	Thymus vulgaris	aerial part	80% EtOH	0.16	[18]

nepedoidin A	*Hyptis suaveolensis*	stem	95% EtOH	0.00021**	[19]
	Melissa officinalis	fresh leaf	Et_2O	+	[20]
	Mentha aquatica	fresh leaf	Et_2O	+	[20]
	Ocimum basilicum	fresh leaf	acetone	≈ 0.0005*	[6]
			EtOAc	≈ 0.017*	[6]
	Origanum vulgare	fresh leaf	Et_2O	+	[20]
	Perilla frutescens	leaf	EtOH	+	[21]
	Salvia officinalis	fresh leaf	Et_2O	+	[20]
	Thymus serpyllum	fresh leaf	Et_2O	+	[20]
	Elsholtzia bodinieri	aerial part	75% acetone	0.00012**	[22]
nepetoidin B	*Hyptis rhomboides*	aerial part	95% EtOH	0.0002**	[23]
	Hyptis suaveolensis	stem	95% EtOH	0.00088	[19]
	Isodon lophanthoides	aerial part	MeOH	0.00095**	[24]
	Meehania urticifolia	whole plant	MeOH	0.00051**	[25]
	Melissa officinalis	fresh leaf	Et_2O	+	[20]
	Mentha aquatica	fresh leaf	Et_2O	+	[20]
	Nepeta teydea	hairy root culture	EtOH	0.0042**	[26]
	Ocimum basilicum	fresh leaf	acetone	≈ 0.001*	[6]
			EtOAc	≈ 0.021*	[6]
	Origanum vulgare	fresh leaf	Et_2O	+	[20]
	Perilla frutescens	leaf	EtOH	+	[21]
	Salvia miltiorrhiza	ns	95% EtOH	37.2×10^{-6}**	[27]
	Salvia officinalis	fresh leaf	Et_2O	+	[20]
	Thymus serpyllum	fresh leaf	Et_2O	+	[20]

ns: not specified; *: contents expressed as percentages of fresh plant material; **: calculated from isolated amounts; +, ++: not specified (++ more amounts than +); EtOH: ethanol; EtOAc: ethyl acetate; Et_2O: diethyl ether; IPA: 2-propanol; MeOH: methanol; values separated by a semicolon represent the minimum and maximum results obtained from at least two analyzed samples.

Table S2A. Contents of the investigated compounds in aerial tissues of *Salvia officinalis*, *Melissa officinalis*, *Origanum vulgare*, *Satureja hortensis*, *Thymus serpyllum*, and *Thymus vulgaris,* determined following various treatments (abbreviations are listed after Table 2F).

Species	Sample No	Treatment conditions	Sample name	Compounds, mg/g		
				**RA (3)**	**NEP A (5)**	**NEP B (8)**
S. officinalis	1	UT	SO/1/UT-*A*	27.1	0.03	0.04
		UT	SO/1/UT-*B*	27.6	0.03	0.05
		DW5	SO/1/DW5	8.83	0.61	1.59
		DW15	SO/1/DW15-*A*	2.07	0.51	0.97
		DW15	SO/1/DW15-*B*	1.86	0.55	0.98
		DW30	SO/1/DW30	0.36	0.33	0.39
		DW45	SO/1/DW45	0.18	0.27	0.17
		DW60	SO/1/DW60	0.15	0.23	0.13
		100 °C, DW60	SO/1/100°C-DW60	29.3	0.14	0.26
	2	UT	SO/2/UT-*A*	13.9	0.11	0.08
		UT	SO/2/UT-*B*	15.9	0.11	0.09
		DW5	SO/2/DW5	8.42	0.61	3.25
		DW15	SO/2/DW15-*A*	4.66	0.66	3.94
		DW15	SO/2/DW15-*B*	4.55	0.62	3.86
		DW30	SO/2/DW30	2.05	0.90	3.93
		DW45	SO/2/DW45	0.75	0.84	3.21
M. officinalis	1	UT	MO/1/UT	20.6	tr	0.04
		DW5	MO/1/DW5	17.7	0.20	0.85
		DW15	MO/1/DW15	14.2	0.21	0.95
		DW30	MO/1/DW30	5.75	0.18	0.80
		DW45	MO/1/DW45	2.18	0.13	0.56
O. vulgare	1	UT	OV/FG/1/UT	13.2	tr	tr
		DW5	OV/1/DW5	2.74	0.61	0.46
		DW15	OV/1/DW15	0.88	0.42	0.30
		DW30	OV/1/DW30	0.48	0.28	tr
	2	UT	OV/2/UT	25.1	0.04	0.04
		DW5	OV/2/DW5	12.3	0.57	0.40
		DW15	OV/2/DW15	4.79	0.73	0.50
		DW30	OV/2/DW30	2.19	0.62	0.44
		DW45	OV/2/DW45	0.99	0.56	0.43
S. hortensis	1	UT	SH/1/UT	11.7	0.04	tr
		DW5	SH/1/DW5	5.00	0.29	0.24
		DW15	SH/1/DW15	2.81	0.22	0.11
		DW30	SH/1/DW30	0.62	0.22	0.18
		DW45	SH/1/DW45	0.06	0.10	0.06
T. vulgaris	1	UT	TV/1/UT	13.7	0.07	0.07
		DW5	TV/1/DW5	3.29	0.23	0.11
		DW15	TV/1/DW15	2.12	0.19	0.10
		DW30	TV/1/DW30	0.27	0.09	0.06
		DW45	TV/1/DW45	0.18	0.06	0.05
T. serpyllum	1	UT	TS/1/UT	13.0	0.05	tr
		DW5	TS/1/DW5	3.78	0.49	0.20
		DW15	TS/1/DW15	1.38	0.54	0.21
		DW30	TS/1/DW30	0.19	0.31	0.09
		DW45	TS/1/DW45	0.07	0.21	0.05

Table S2B. Contents of the investigated compounds in aerial tissues of field-grown and in vitro-grown *Mentha* species, determined following various treatments (abbreviations are listed after Table 2F).

Species	Origin	Sample No	Treatment conditions	Sample name	Compounds, mg/g		
					**RA (3)**	**NEP A (5)**	**NEP B (8)**
M. × *piperita*	field-grown	1	UT	MP/FG/1/UT	12.1	0.25	tr
			DW5	MP/FG/1/DW5	5.96	0.46	0.21
			DW15	MP/FG/1/DW15	3.83	0.47	0.22
			DW30	MP/FG/1/DW30	1.22	0.36	0.14
			DW45	MP/FG/1/DW45	0.03	0.11	tr
			100 °C, DW45	MP/FG/1/100°C-DW45	12.5	0.28	tr
	field-grown, filter tea from local store	2	UT	MP/FG/2/UT	8.51	0.20	0.10
			DW5	MP/FG/2/DW5	5.92	0.60	0.38
			DW15	MP/FG/2/DW15	3.47	0.62	0.48
			DW30	MP/FG/2/DW30	1.47	0.54	0.46
			DW45	MP/FG/2/DW45	0.85	0.34	0.30
	in vitro	1	UT	MP/IV/1/UT	8.59	tr	0.06
			DW5	MP/IV/1/DW5	4.56	0.42	2.03
			DW15	MP/IV/1/DW15	1.16	0.62	4.18
			DW30	MP/IV/1/DW30	0.14	0.36	3.94
			DW45	MP/IV/1/DW45	0.07	0.18	2.86
M. spicata	field-grown	1	UT	MS/FG/1/UT	28.8	0.14	0.16
			DW5	MS/FG/1/DW5	22.8	0.28	0.75
			DW15	MS/FG/1/DW15	11.4	0.32	1.08
			DW30	MS/FG/1/DW30	1.99	0.17	0.73
			DW45	MS/FG/1/DW45	0.21	0.10	0.21
			100 °C, DW45	MS/FG/1/100°C-DW45	31.5	0.12	0.13
		2	UT	MS/FG/2/UT	13.4	tr	tr
			DW5	MS/FG/2/DW5	7.05	0.22	0.33
			DW15	MS/FG/2/DW15	0.49	0.14	0.22
			DW30	MS/FG/2/DW30	0.20	0.08	0.10
			DW45	MS/FG/2/DW45	0.06	tr	tr
	in vitro	1	UT	MS/IV/1/UT	10.9	0.06	0.10
			DW5	MS/IV/1/DW5	5.18	0.58	4.57
			DW15	MS/IV/1/DW15	1.12	0.60	6.13
			DW45	MS/IV/1/DW45	0.22	0.07	0.50
M. aquatica	field-grown	1	UT	MA/FG/1/UT	44.1	tr	tr
			DW5	MA/FG/1/DW5	15.2	1.34	1.09
			DW15	MA/FG/1/DW15	5.58	0.90	0.60
			DW30	MA/FG/1/DW30	0.77	0.65	0.32
			DW45	MA/FG/1/DW45	0.20	0.44	0.12
	in vitro	1	UT	MA/IV/1/UT-*A*	22.4	0.24	0.24
			UT	MA/IV/1/UT-*B*	22.9	0.23	0.24
			DW5	MA/IV/1/DW5	16.0	1.93	4.40
			DW15	MA/IV/1/DW15-*A*	5.21	2.17	8.18
			DW15	MA/IV/1/DW15-*B*	5.25	2.23	8.54
			DW30	MA/IV/1/DW30	1.74	2.61	11.5
			DW45	MA/IV/1/DW45	0.36	1.37	10.0
			DW135	MA/IV/1/DW135-*A*	0.25	0.31	3.05
			DW135	MA/IV/1/DW135-*B*	0.23	0.32	3.40
			DW15, 100 °C, DW120	MA/IV/1/DW15-100°C-DW120-*A*	6.28	2.57	8.33
			DW15, 100 °C, DW120	MA/IV/1/DW15-100°C-DW120-*B*	6.62	2.59	8.65
		2	UT	MA/IV/2/UT	17.3	tr	0.03
			DW5	MA/IV/2/DW5	11.8	1.43	3.62
			DW15	MA/IV/2/DW15	3.8	2.38	7.87
			DW30	MA/IV/2/DW30	0.48	1.33	8.41
			DW45	MA/IV/2/DW45	0.12	0.76	6.51

Table S2C. Contents of the investigated compounds in aerial tissues and roots of *Salvia verticillata,* determined following various treatments (abbreviations are listed after Table 2F).

Species	Organ	Sample No	Treatment conditions	Sample name	Compounds, mg/g					
					**RA Gl (1)**	**RA (3)**	**NEP A (5)**	**NEP B (8)**	**SAA O (4)**	**SAA Q (7)**
S. verticillata	roots	1	UT	SV/R/1/UT-*A*	0.48	42.2	0.07	0.04	48.2	tr
				SV/R/1/UT-*B*	0.49	42.3	0.07	0.04	50.4	tr
		2		SV/R/2/UT-*A*	0.93	51.2	0.08	0.05	31.3	tr
				SV/R/2/UT-*B*	0.90	49.4	0.08	0.06	29.3	tr
				SV/R/2/UT-*C*	0.94	50.3	0.07	0.06	30.3	tr
		3		SV/R/3/UT-*A*	8.26	39.4	tr	0.06	46.7	tr
				SV/R/3/UT-*B*	8.18	38.6	tr	0.06	45.8	tr
		4		SV/R/4/UT	4.51	27.8	tr	0.05	20.7	tr
		5		SV/R/5/UT	0.31	27.7	tr	0.05	28.5	tr
			DW5	SV/R/5/DW5	0.11	5.08	0.13	0.41	10.1	0.07
			DW15	SV/R/5/DW15	tr	0.46	0.09	0.20	1.07	0.04
			DW30	SV/R/5/DW30	tr	0.42	0.08	0.19	0.96	0.03
			DW45	SV/R/5/DW45	tr	0.27	0.06	0.18	0.42	tr
		6	UT	SV/R/6/UT-*A*	tr	46.6	0.04	0.08	60.5	tr
				SV/R/6/UT-*B*	tr	42.6	0.04	0.08	55.0	tr
		7		SV/R/7/UT-*A*	0.06	33.1	tr	0.05	59.0	tr
				SV/R/7/UT-*B*	0.06	31.6	tr	0.05	55.3	tr
				SV/R/7/UT-*C*	0.05	31.7	tr	0.05	61.4	tr
				SV/R/7/UT-*D*	0.05	32.9	tr	0.05	61.9	tr
		8		SV/R/8/UT-*A*	0.09	57.1	tr	0.07	80.1	tr
				SV/R/8/UT-*B*	0.08	53.8	tr	0.07	77.0	tr
			DW5	SV/R/8/DW5	0.04	14.2	0.24	1.35	33.3	0.42
			DW15	SV/R/8/DW15	-	1.92	0.19	0.67	6.77	0.29
			DW30	SV/R/8/DW30	-	1.11	0.11	0.43	1.87	0.19
			DW45	SV/R/8/DW45	-	0.19	0.05	0.12	0.25	tr
		9	UT	SV/R/9/UT-*A*	3.09	45.8	tr	tr	25.2	tr
				SV/R/9/UT-*B*	3.17	47.2	tr	tr	24.4	tr
			DW45	SV/R/9/DW45	0.05	1.52	tr	0.09	0.20	tr
			100 °C, DW45	SV/R/9/100°C-DW45-*A*	2.58	38.4	0.05	0.20	22.7	tr
				SV/R/9/100°C-DW45-*B*	2.74	37.7	0.04	0.19	21.4	tr
	aerial parts	2	UT	SV/AP/2/UT	tr	17.8	tr	0.03	0.52	-
		3		SV/AP/3/UT-*A*	-	59.4	tr	tr	1.34	-
				SV/AP/3/UT-*B*	-	58.8	tr	tr	1.33	-
		4		SV/AP/4/UT	-	35.4	tr	0.03	4.09	-
		5		SV/AP/5/UT	0.31	13.0	tr	tr	0.27	tr
			DW5	SV/AP/5/DW5	0.11	4.38	0.13	1.59	0.15	tr
			DW15	SV/AP/5/DW15	tr	0.42	0.12	1.32	0.10	tr
			DW30	SV/AP/5/DW30	tr	0.62	0.09	1.10	0.09	tr
			DW45	SV/AP/5/DW45	tr	0.51	0.08	0.99	0.07	tr

Table S2D. Contents of the investigated compounds in aerial tissues and roots of *Salvia pratensis,* determined following various treatments (abbreviations are listed after Table 2F).

Species	Organ	Sample No	Treatment conditions	Sample name	Compounds, mg/g					
					**RA Gl (1)**	**RA (3)**	**NEP A (5)**	**NEP B (8)**	**SAA K (2)**	**SAA P (6)**
S. pratensis	roots	1	UT	SP/R/1/UT-*A*	3.07	25.1	0.07	0.06	41.6	0.05
				SP/R/1/UT-*B*	3.14	24.7	0.06	0.06	40.4	0.05
				SP/R/1/UT-*C*	3.01	22.6	0.06	0.06	38.2	0.04
				SP/R/1/UT-*D*	3.16	23.5	0.06	0.06	39.8	0.05
			100 °C, DW45	SP/R/1/100°C-DW45-*A*	2.99	20.1	0.20	0.14	38.7	0.16
				SP/R/1/100°C-DW45-*B*	2.68	19.4	0.19	0.14	36.3	0.16
			DW5	SP/R/1/DW5	0.99	2.46	0.51	0.40	10.5	0.42
			DW15	SP/R/1/DW15	0.33	0.63	0.49	0.39	2.94	0.41
			DW30	SP/R/1/DW30	0.03	0.12	0.10	0.10	0.07	0.08
			DW45	SP/R/1/DW45	-	0.05	tr	0.05	tr	tr
		2	UT	SP/R/2/UT	2.41	16.2	0.07	0.05	43.2	tr
		3	UT	SP/R/3/UT-*A*	2.02	16.4	0.07	0.07	41.9	0.05
				SP/R/3/UT-*B*	1.96	16.3	0.06	0.07	40.7	0.05
	aerial parts	1	NT	SP/AP/1/UT-*A*	0.13	22.2	tr	tr	tr	-
				SP/AP/1/UT-*B*	0.13	21.2	tr	tr	tr	-
			DW5	SP/AP/1/DW5	tr	5.82	0.22	0.06	tr	-
			DW15	SP/AP/1/DW15	-	0.37	0.14	tr	-	-
			DW30	SP/AP/1/DW30	-	0.10	0.07	tr	-	-
			DW45	SP/AP/1/DW45	-	0.05	tr	tr	-	-
		2	UT	SP/AP/2/UT	-	23.4	tr	tr	tr	-
		3		SP/AP/3/UT	tr	18.6	tr	tr	tr	-

Table S2E. Contents of the investigated compounds in aerial tissues and roots of *Salvia nemorosa,* determined following various treatments (abbreviations are listed after Table 2F).

Species	Organ	Sample No	Treatment conditions	Sample name	Compounds, mg/g					
					**RA Gl (1)**	**RA (3)**	**NEP A (5)**	**NEP B (8)**	**SAA K (2)**	**SAA P (6)**
S. nemorosa	roots	1	UT	SN/R/1/UT	2.89	10.8	tr	0.05	14.3	0.04
		2		SN/R/2/UT-*A*	1.12	18.1	0.07	tr	17.7	0.05
				SN/R/2/UT-*B*	1.13	18.3	0.07	tr	17.3	0.05
		3		SN/R/3/UT-*A*	1.28	14.5	0.06	0.05	14.9	0.04
				SN/R/3/UT-*B*	1.30	15.3	0.06	0.05	15.0	0.04
			DW5	SN/R/3/DW5	0.43	2.60	0.54	0.61	5.41	0.45
			DW15	SN/R/3/DW15	0.06	0.59	0.34	0.35	0.78	0.28
			DW30	SN/R/3/DW30	tr	0.29	0.13	0.16	0.09	0.11
			DW45	SN/R/3/DW45	tr	0.18	0.07	0.09	0.05	0.06
	aerial parts	1	UT	SN/AP/1/UT	0.27	19.3	tr	tr	tr	-
		2		SN/AP/2/UT-*A*	0.46	20.8	tr	tr	tr	tr
				SN/AP/2/UT-*B*	0.46	21.8	tr	tr	tr	tr
			DW5	SN/AP/2/DW5	0.35	4.42	0.23	1.24	tr	tr
			DW15	SN/AP/2/DW15	tr	0.23	0.09	0.35	-	tr
			DW30	SN/AP/2/DW30	-	0.18	tr	0.21	-	tr
			DW45	SN/AP/2/DW45	-	0.27	tr	tr	-	tr

Table S2F. Contents of the investigated compounds in aerial tissues and roots of *Salvia sclarea,* determined following various treatments (abbreviations are listed after Table 2F).

Species	Organ	Sample No	Treatment conditions	Sample name	Compounds, mg/g					
					**RA Gl (1)**	**RA (3)**	**NEP A (5)**	**NEP B (8)**	**SAA K (2)**	**SAA P (6)**
S. sclarea	roots	1	UT	SS/R/1/UT-*A*	0.05	3.33	tr	tr	5.29	tr
				SS/R/1/UT-*B*	0.05	3.08	tr	tr	5.09	tr
		2		SS/R/2/UT-*A*	0.08	3.92	0.06	tr	5.68	0.04
				SS/R/2/UT-*B*	0.08	4.37	0.06	tr	7.05	0.05
			DW5	SS/R/2/DW5	-	0.57	0.22	0.45	0.10	0.55
			DW15	SS/R/2/DW15	-	0.11	0.14	0.36	tr	0.35
			DW30	SS/R/2/DW30	-	0.06	0.09	0.22	-	0.21
			DW45	SS/R/2/DW45	-	tr	tr	0.10	-	0.08
	aerial parts	1	UT	SS/AP/1/UT-*A*	tr	20.0	tr	tr	2.60	tr
				SS/AP/1/UT-*B*	tr	20.1	tr	tr	2.50	tr
		2		SS/AP/2/UT-*A*	tr	17.2	tr	tr	2.23	tr
				SS/AP/2/UT-*B*	tr	16.9	tr	tr	2.22	tr
			DW5	SS/AP/2/DW5	tr	3.46	0.29	0.86	0.32	0.12
			DW15	SS/AP/2/DW15	-	2.29	0.24	0.69	0.20	0.10
			DW30	SS/AP/2/DW30	-	1.40	0.18	0.53	0.05	0.07
			DW45	SS/AP/2/DW45	-	0.42	0.12	0.32	tr	0.05

To prepare the extracts, the following treatment conditions were used.

UT: pulverized tissues were extracted directly with 80% MeOH at 60 °C without pretreatment in water (untreated);

DW5, DW15, DW30, DW45, DW60, DW135: pulverized tissues were suspended in distilled water and incubated at room temperature for 5 min (DW5), 15 min (DW15), 30 min (DW30), 45 min (DW45), 60 min (DW60), and 135 min (DW135);

100 ℃, DW45 and 100 ℃, DW60: pulverized tissues were suspended in hot distilled water and incubated at 100 °C for 2 min, then incubated at room temperature for an additional 45 min or 60 min.

DW15, 100 ℃, DW120: Pulverized tissues were suspended in distilled water and incubated at room temperature for 15 min, then heated at 100 °C for 2 min, followed by a further 120 min incubation at room temperature.

After the aqueous incubation steps, absolute methanol (MeOH) was added to the suspensions to obtain a final concentration of 80% (v/v) MeOH. The samples were subsequently heated at 60 °C.

Compound numbers and abbreviations correspond to rosmarinic acid-3-*O*-β-D-glucoside (RA Gl, **1**), salvianolic acid K (SAA K, **2**), rosmarinic acid (RA, **3**), salvianolic acid O (SAA O, **4**), nepetoidin A (NEP A, **5**), salvianolic acid P (SAA P, **6**), salvianolic acid Q (SAA Q, **7**), and nepetoidin B (NEP B, **8**).

-, not detected: compounds were not detected by HR-MS based on the deprotonated molecular ion signal in negative ionization mode.

tr, trace amount: the amount of the compound was lower than the smallest amount included in the corresponding calibration curve and therefore could not be quantified accurately.

Italic letters *A*–*D* in the sample names indicate independently prepared extracts that were analyzed separately. Based on these parallel measurements, compounds **1**, **2**, **3**, **4**, **5**, **6**, and **8** were quantified in 13, 8, 25, 7, 16, 5, and 16 parallel extracts, respectively, with average Relative Percent Difference (RPD) values of 3.1%, 5.3%, 4.4%, 4.3%, 4.2%, 4.4%, and 3.8%, respectively. These measurements resulted in an overall average RPD of 4.1% across all 90 parallel quantifications. Furthermore, two plant samples (SP/R/1/UT-*A*–*D*, Table S2D and SV/R/7/UT-*A*–*D*, Table S2C) were analyzed in quadruplicate, whereas one plant sample (SV/R/2/UT, Table S2C) was analyzed in triplicate. Based on these replicate analyses, the Relative Standard Deviation (RSD) values calculated for compounds **1**, **2**, **3**, **4**, **5**, **6**, and **8** were 5.0%, 3.5%, 3.0%, 4.2%, 7.8%, 10.5%, and 3.4%, respectively, with an overall average RSD of 4.8%.

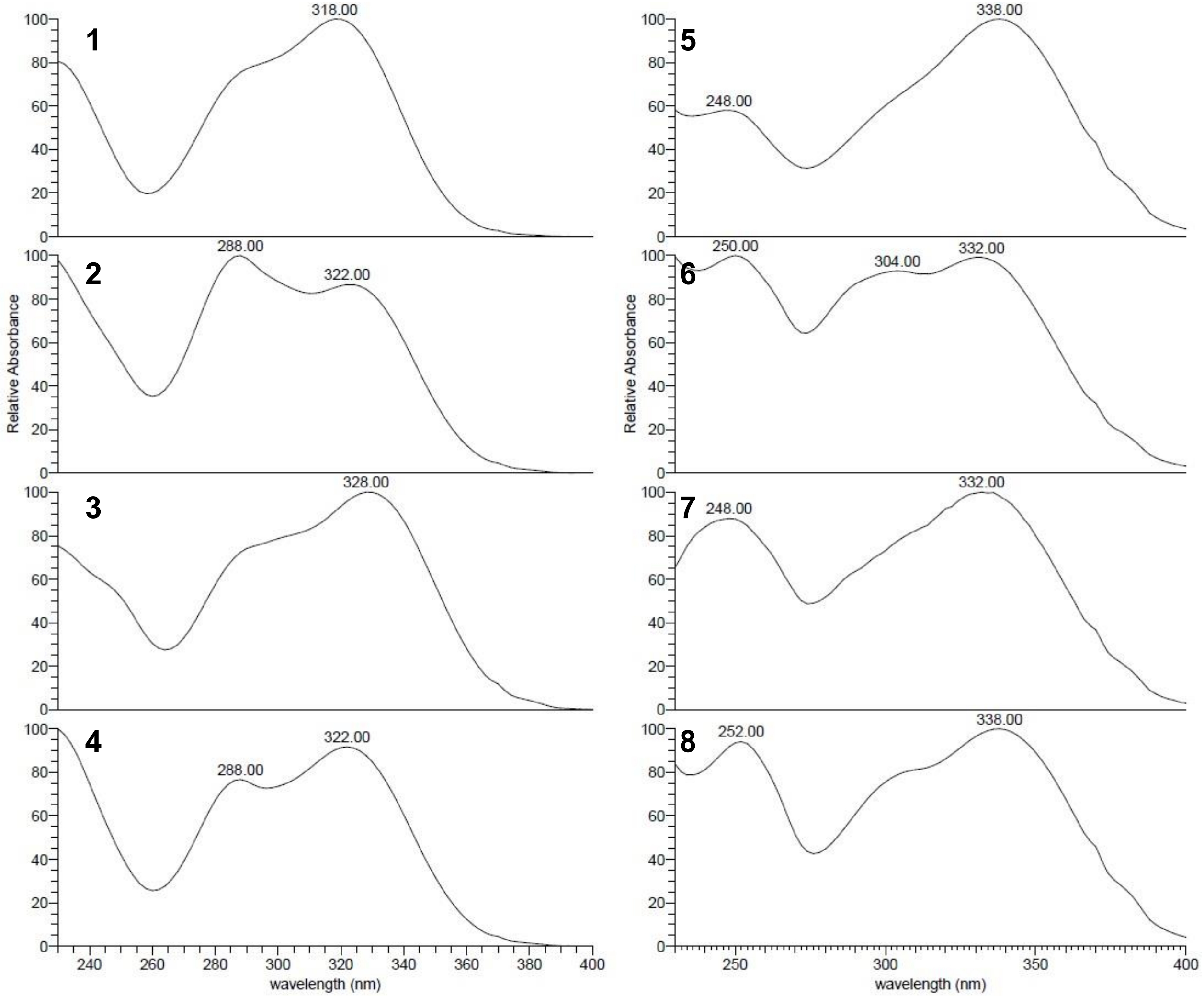

Figure S1. UV spectra of rosmarinic acid-3-*O*-β-D-glucoside (**1**), salvianolic acid K (**2**), rosmarinic acid (**3**), salvianolic acid O (**4**), nepetoidin A (**5**), salvianolic acid P (**6**), salvianolic acid Q (**7**), and nepetoidin B (**8**), as obtained from HPLC-UV chromatograms.

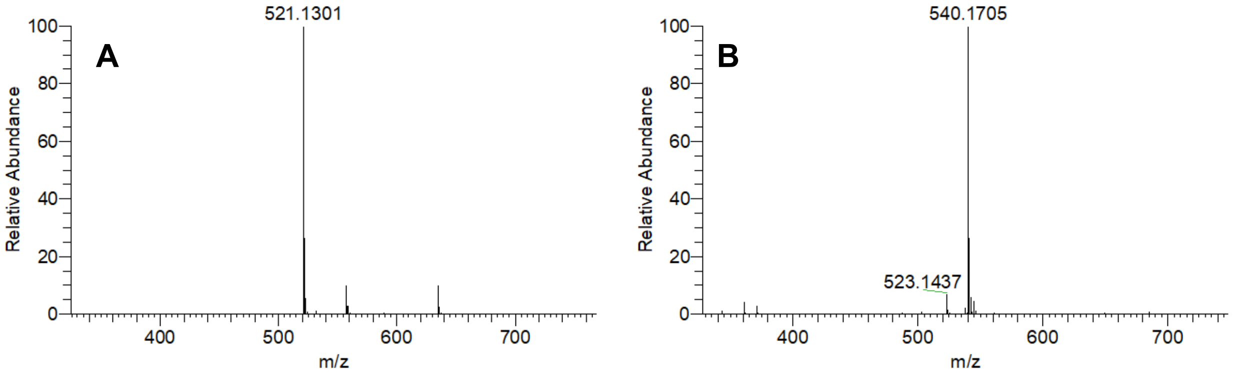

Figure S2A. High-resolution mass spectra of compound **1** (rosmarinic acid-3-*O*-β-D-glucoside) obtained by HPLC-HR-MS in negative (A) and positive (B) ionization modes.

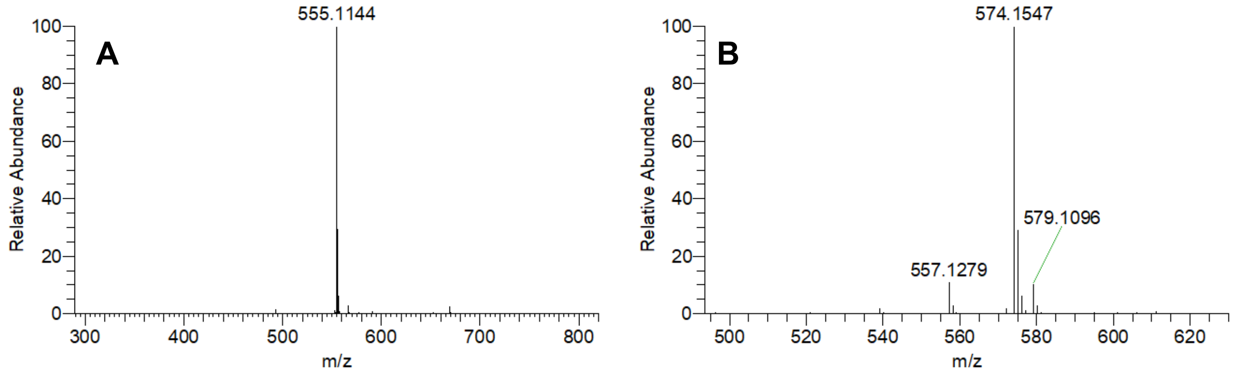

Figure S2B. High-resolution mass spectra of compound **2** (salvianolic acid K) obtained by HPLC-HR-MS in negative (A) and positive (B) ionization modes.

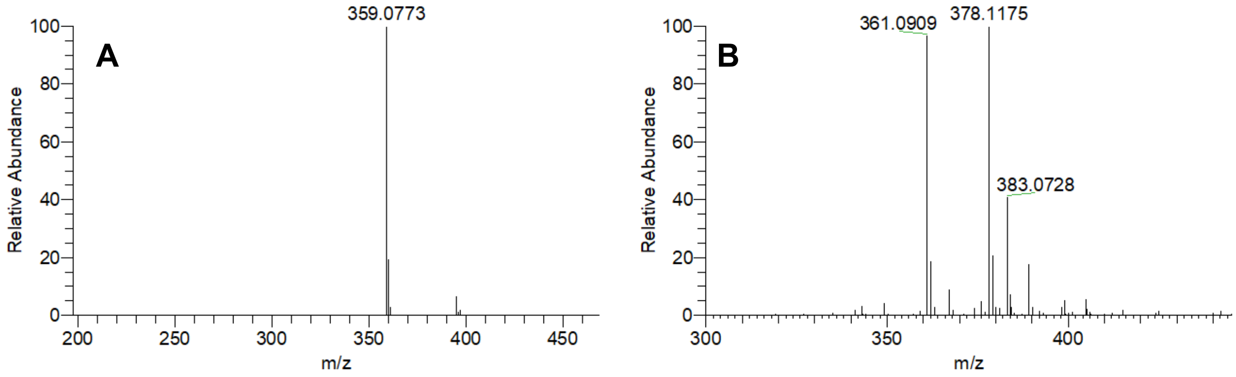

Figure S2C. High-resolution mass spectra of compound **3** (rosmarinic acid) obtained by HPLC-HR-MS in negative (A) and positive (B) ionization modes.

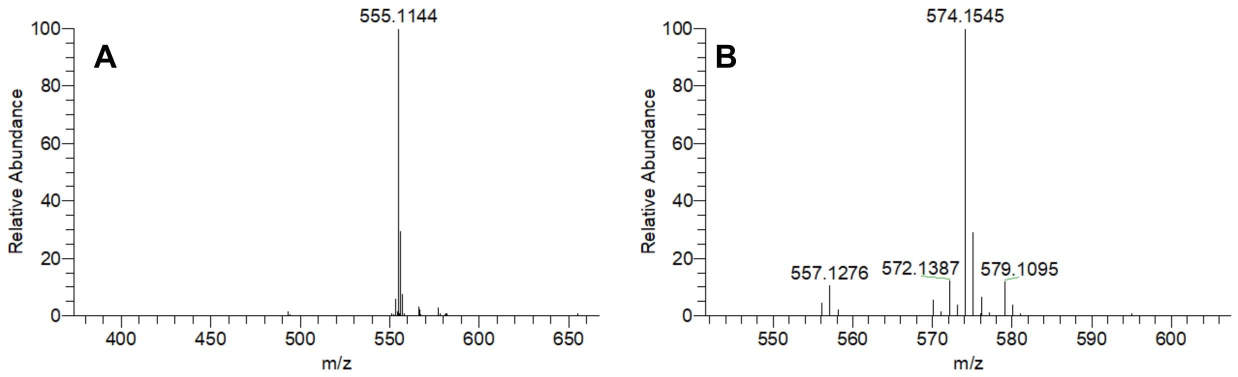

Figure S2D. High-resolution mass spectra of compound **4** (salvianolic acid O) obtained by HPLC-HR-MS in negative (A) and positive (B) ionization modes.

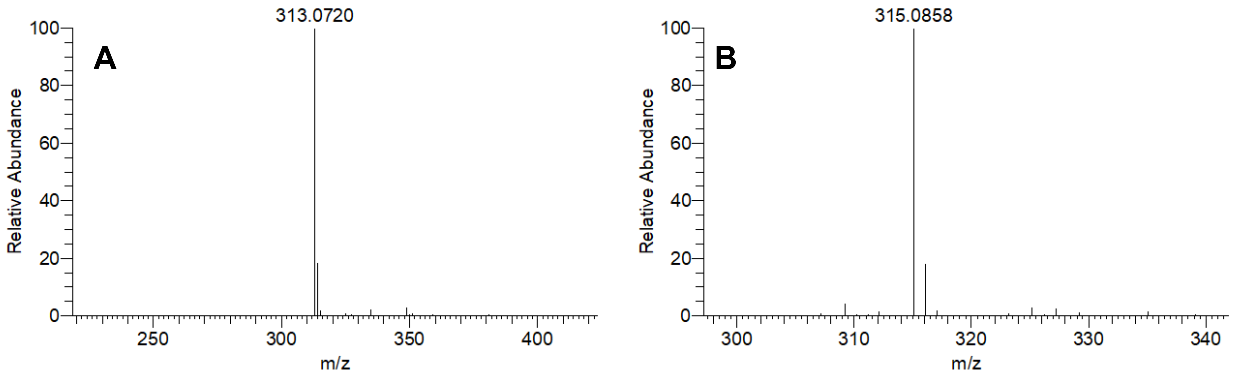

Figure S2E. High-resolution mass spectra of compound **5** (nepetoidin A) obtained by HPLC-HR-MS in negative (A) and positive (B) ionization modes.

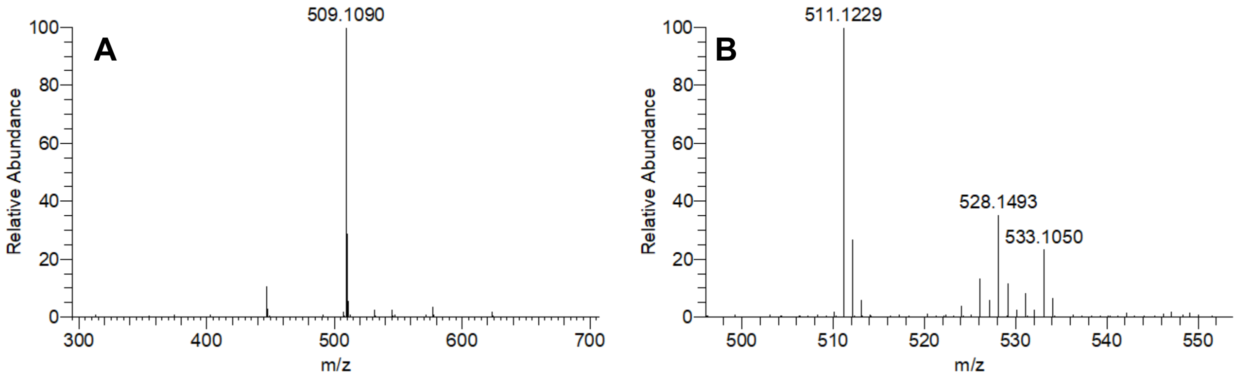

Figure S2F. High-resolution mass spectra of compound **6** (salvianolic acid P) obtained by HPLC-HR-MS in negative (A) and positive (B) ionization modes.

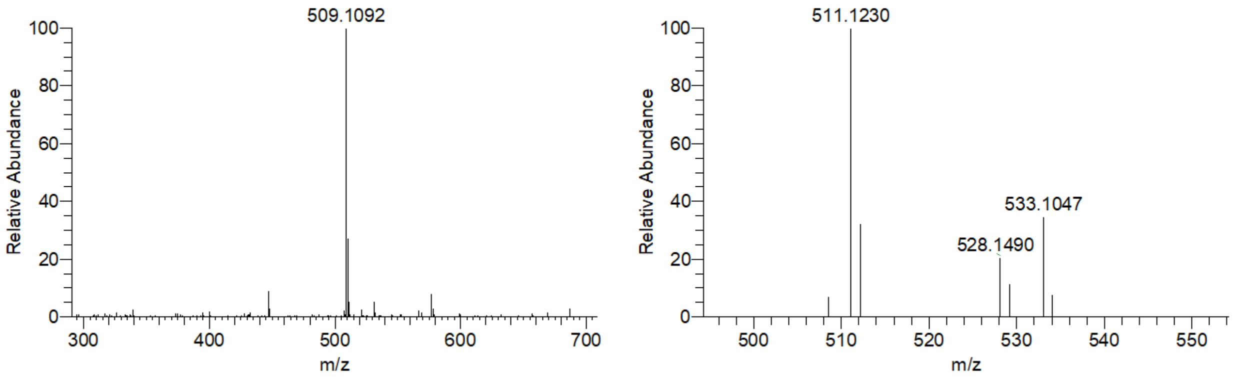

Figure S2G. High-resolution mass spectra of compound **7** (salvianolic acid Q) obtained by HPLC-HR-MS in negative (A) and positive (B) ionization modes.

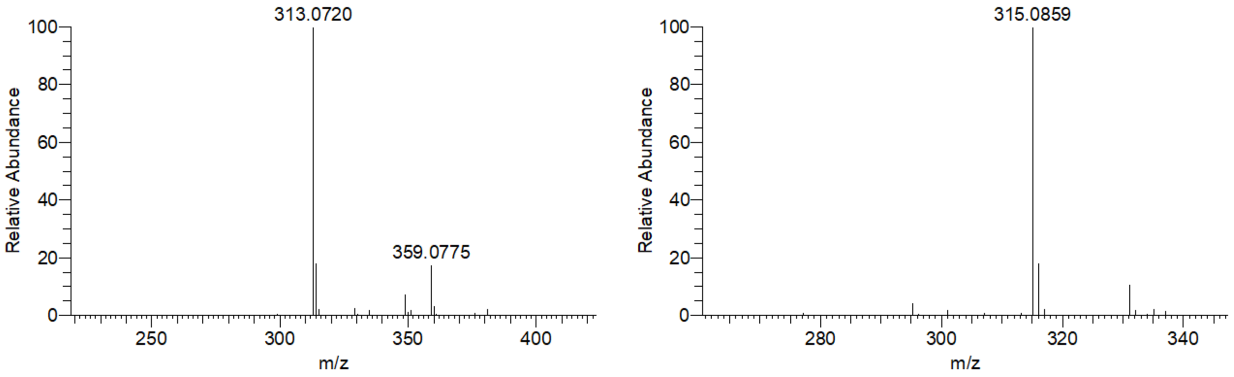

Figure S2H. High-resolution mass spectra of compound **8** (nepetoidin B) obtained by HPLC-HR-MS in negative (A) and positive (B) ionization modes.

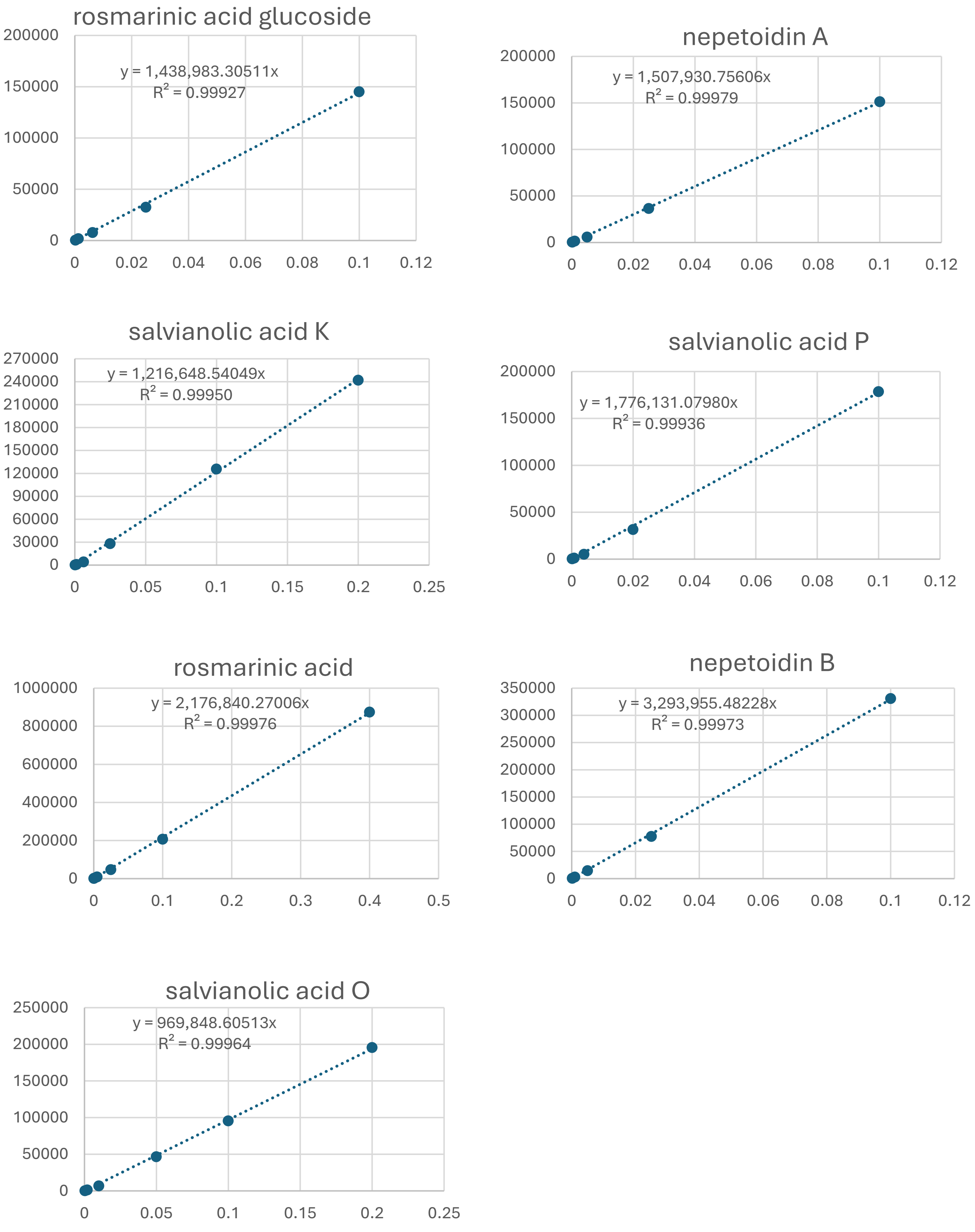

Figure S3. HPLC-UV calibration curves of the isolated compounds (x: concentration, mg/mL; y: peak area). The HPLC-UV chromatograms were recorded across a wavelength range of 230–600 nm, detecting the total signal intensity within this range.

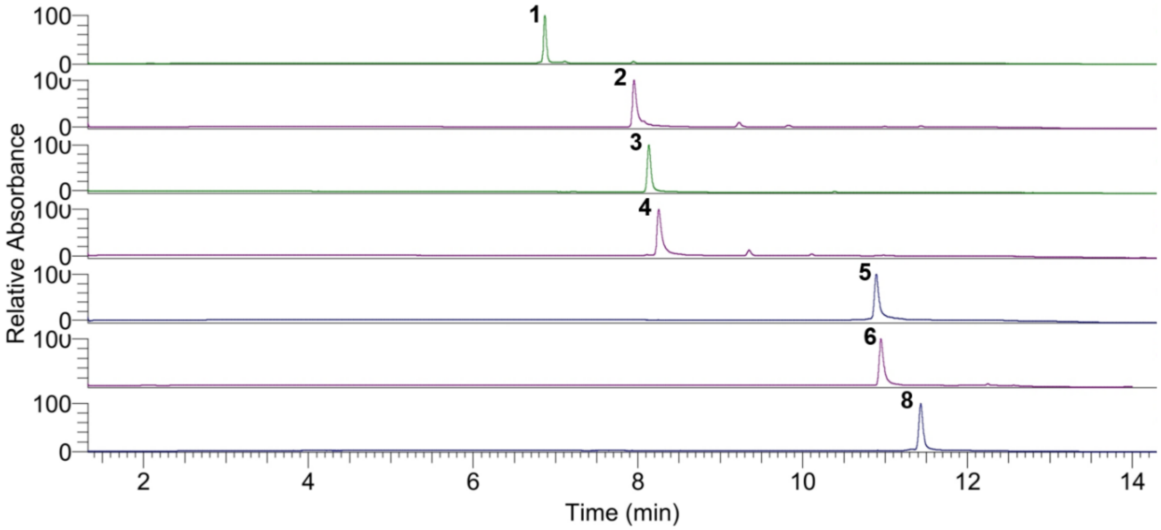

Figure S4. HPLC-UV analysis of the isolated compounds (**1**–**6** and **8**). Peak numbers correspond to the numbering of the identified compounds: rosmarinic acid-3-*O*-β-D-glucoside (**1**), salvianolic acid K (**2**), rosmarinic acid (**3**), salvianolic acid O (**4**), nepetoidin A (**5**), salvianolic acid P (**6**), and nepetoidin B (**8**).

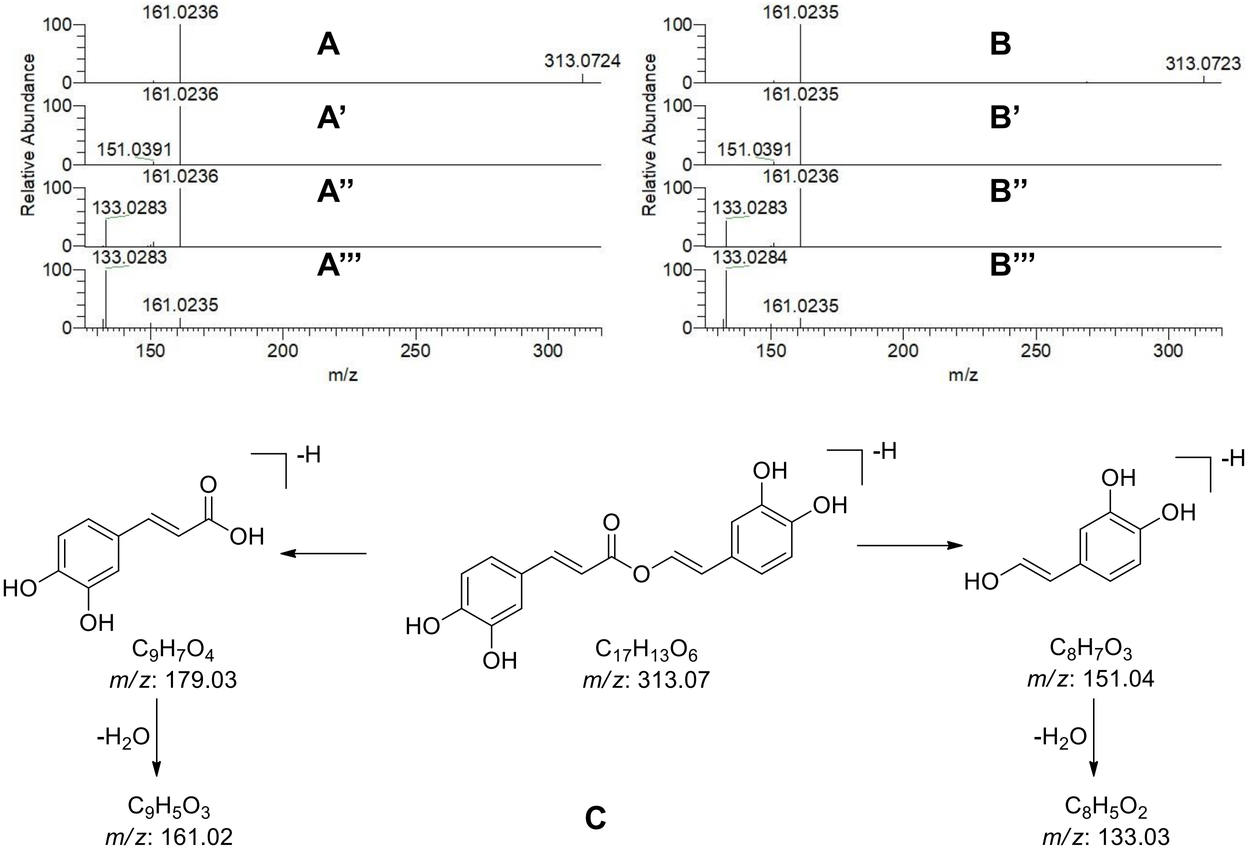

Figure S5. HR-MS/MS spectra of nepetoidin A (A–A′′′) and nepetoidin B (B–B′′′) acquired from the deprotonated molecular ions ($[M–H]^-$, *m/z* 313) in negative ionization mode at collision-induced dissociation energies of 10 (A, B), 15 (A′, B′), 30 (A′′, B′′), and 45 eV (A′′′, B′′′). Panel C shows the proposed fragmentation pathways of nepetoidin A.

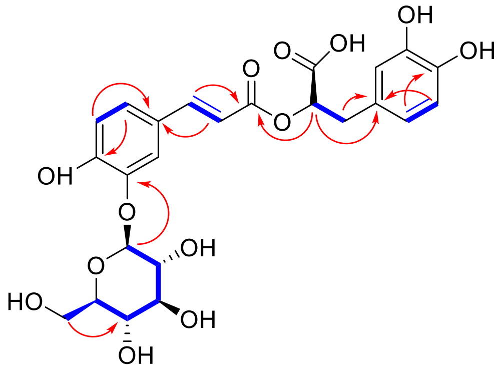

1

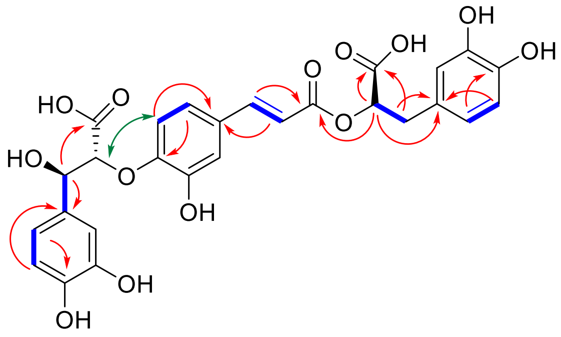

2

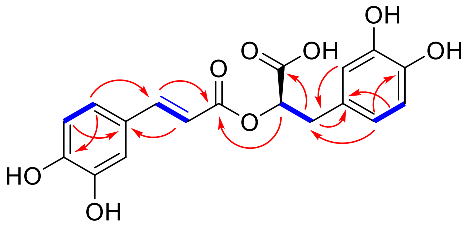

3

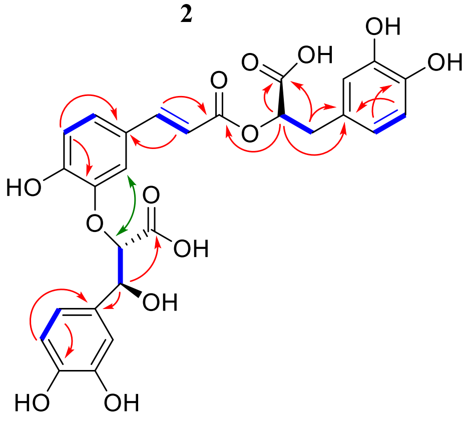

4

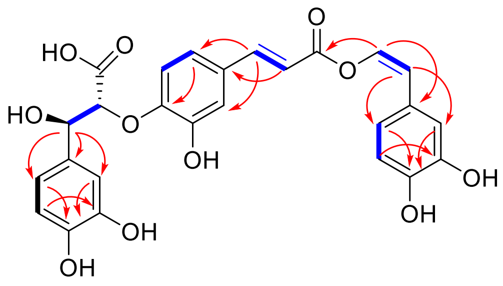

6

Figure S6. Key 2D-NMR correlations (^{1}H–^{1}H COSY; blue, ^{1}H–^{1}H NOESY; green, ^{1}H–^{13}C HMBC; red) of isolated parent compounds (**1–4**) and conversion product (**6**). For conversion products (**5**, **8**), only ^{1}H–^{1}H COSY was recorded therefore, the detailed correlations are not shown. Moreover, conversion product (**7**) was not characterised through NMR.

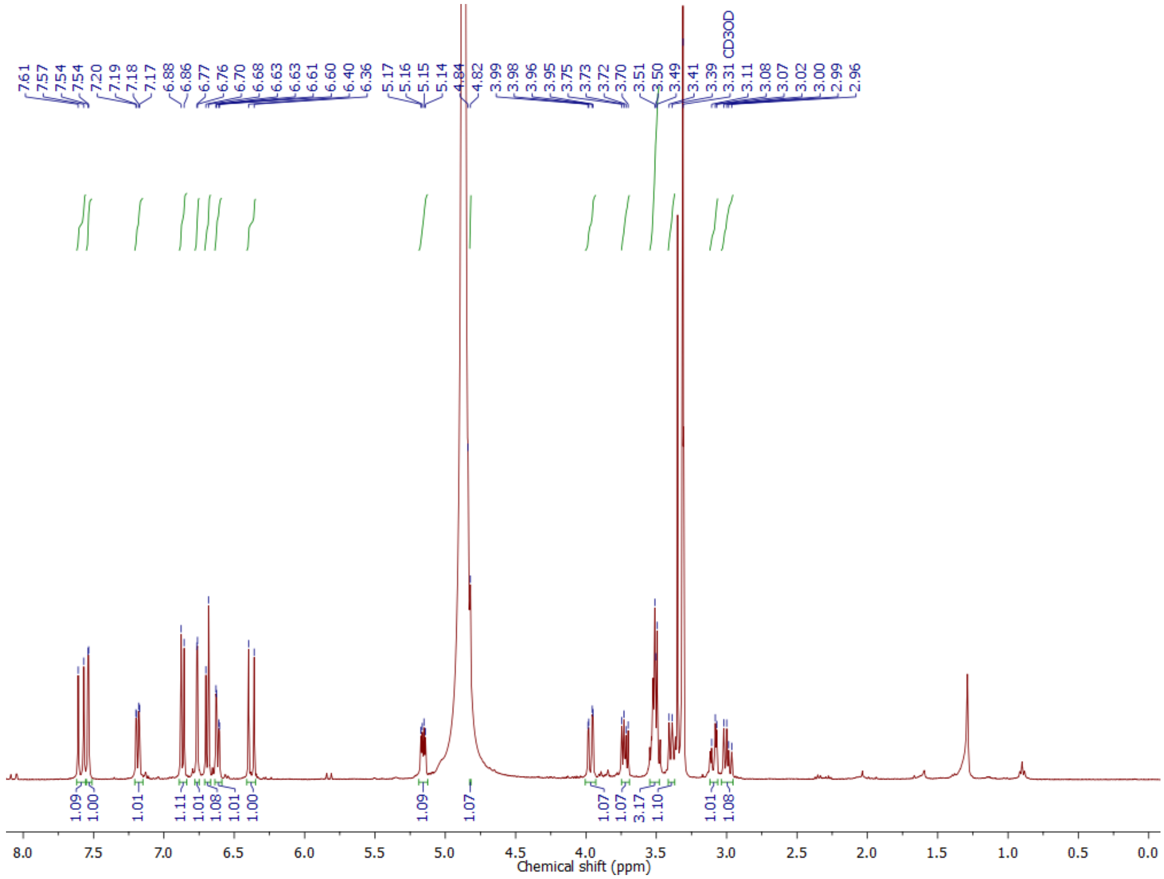

Figure S7. ^{1}H NMR (Methanol-d_4, 500 MHz) spectrum of compound **1**.

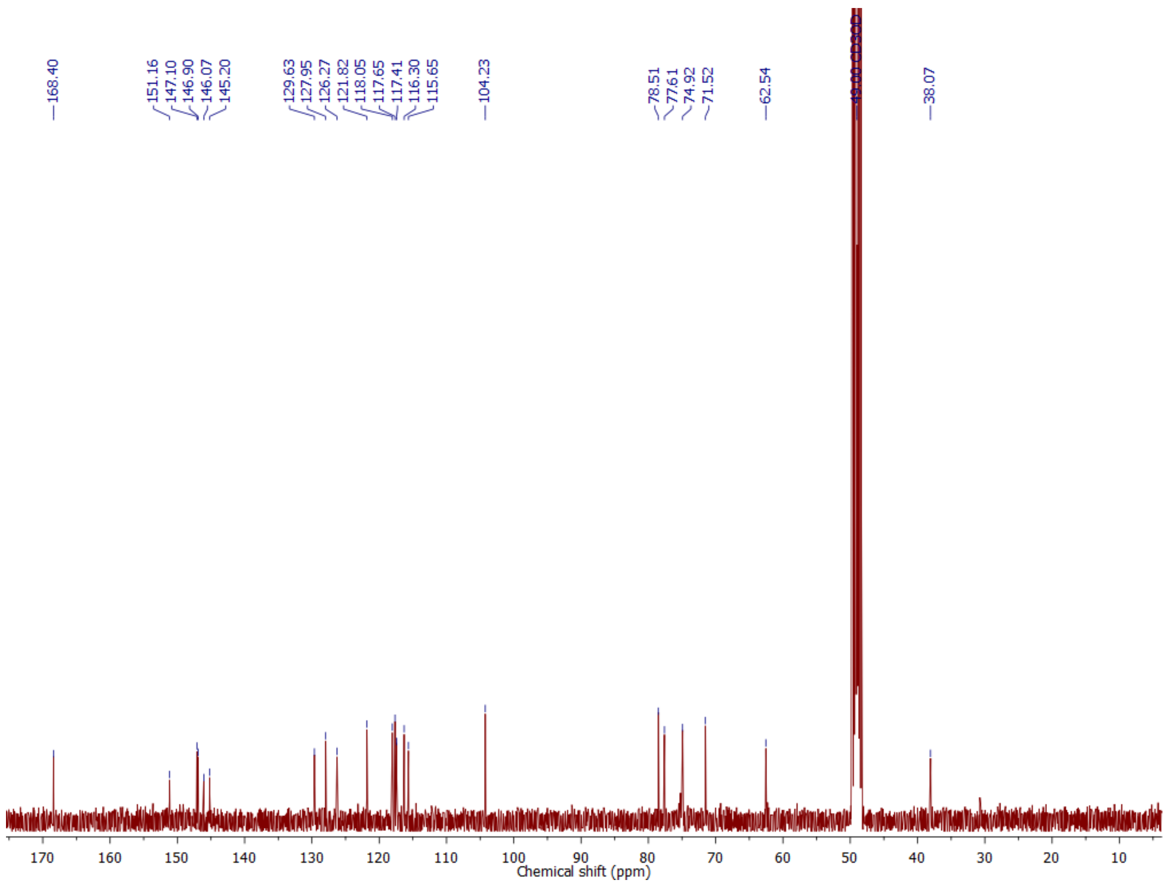

Figure S8. ^{13}C NMR (Methanol-*d*[4], 125 MHz) spectrum of compound **1**.

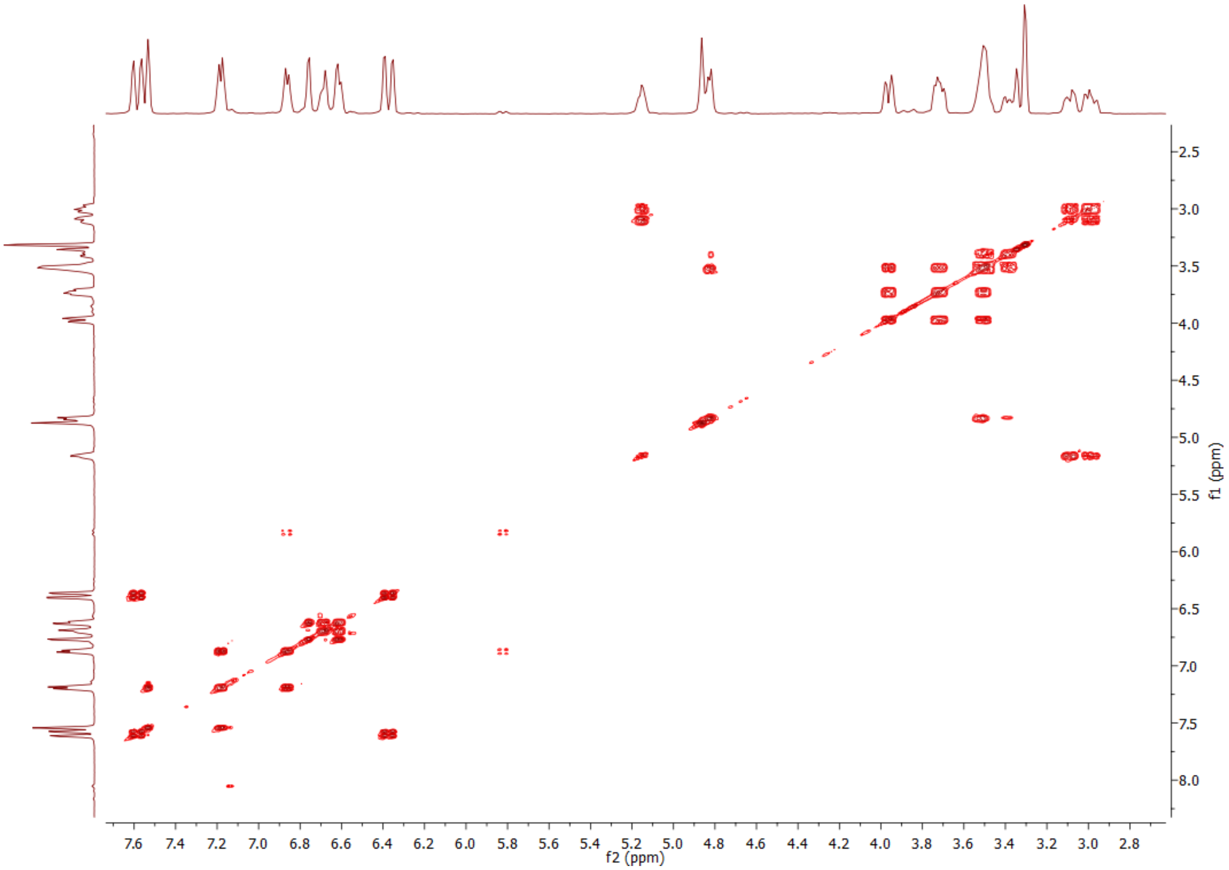

Figure S9. ^{1}H-^{1}H COSY (Methanol-*d*$_4$, 500 MHz) spectrum of compound **1**.

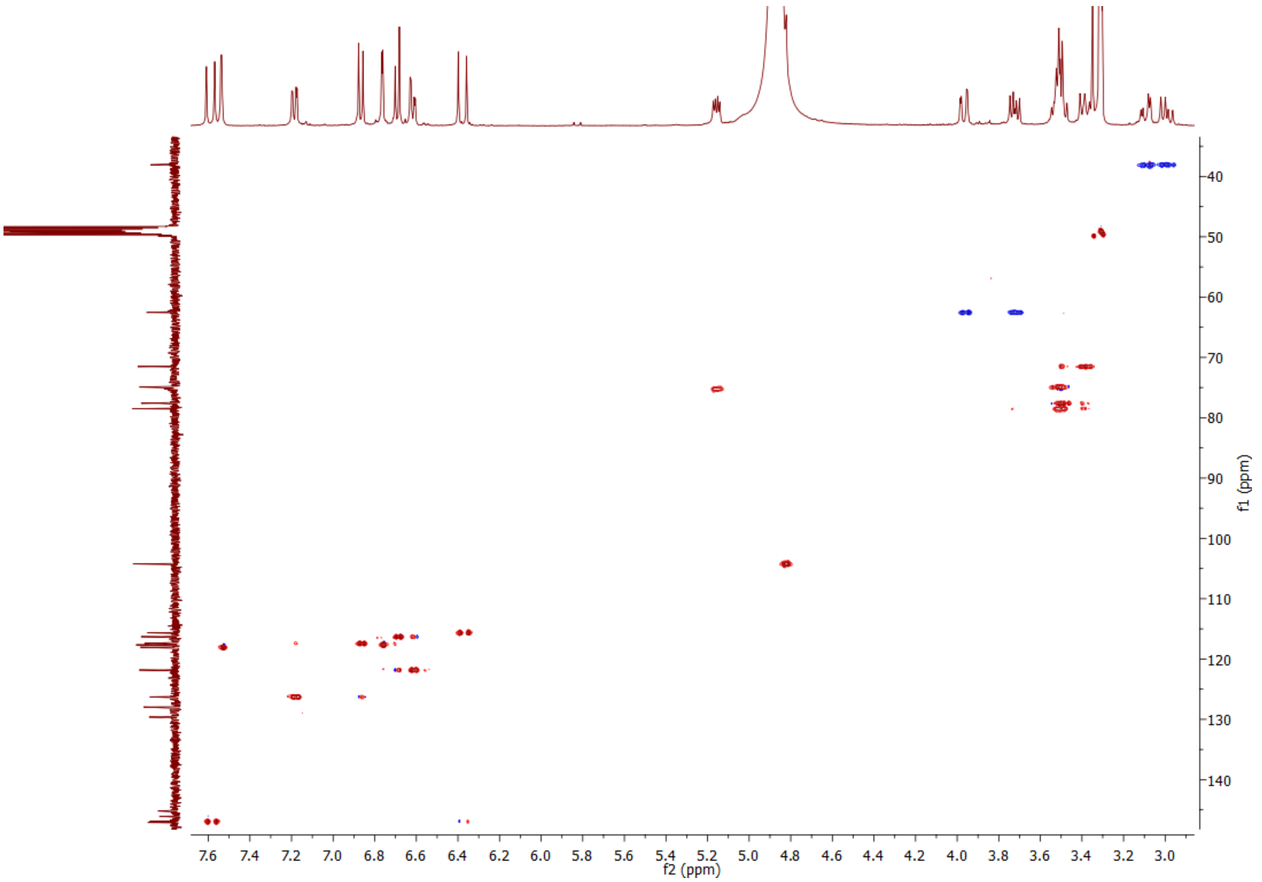

Figure S10. ^{1}H–^{13}C HSQC (Methanol-d_4, 500/125 MHz) spectrum of compound **1**.

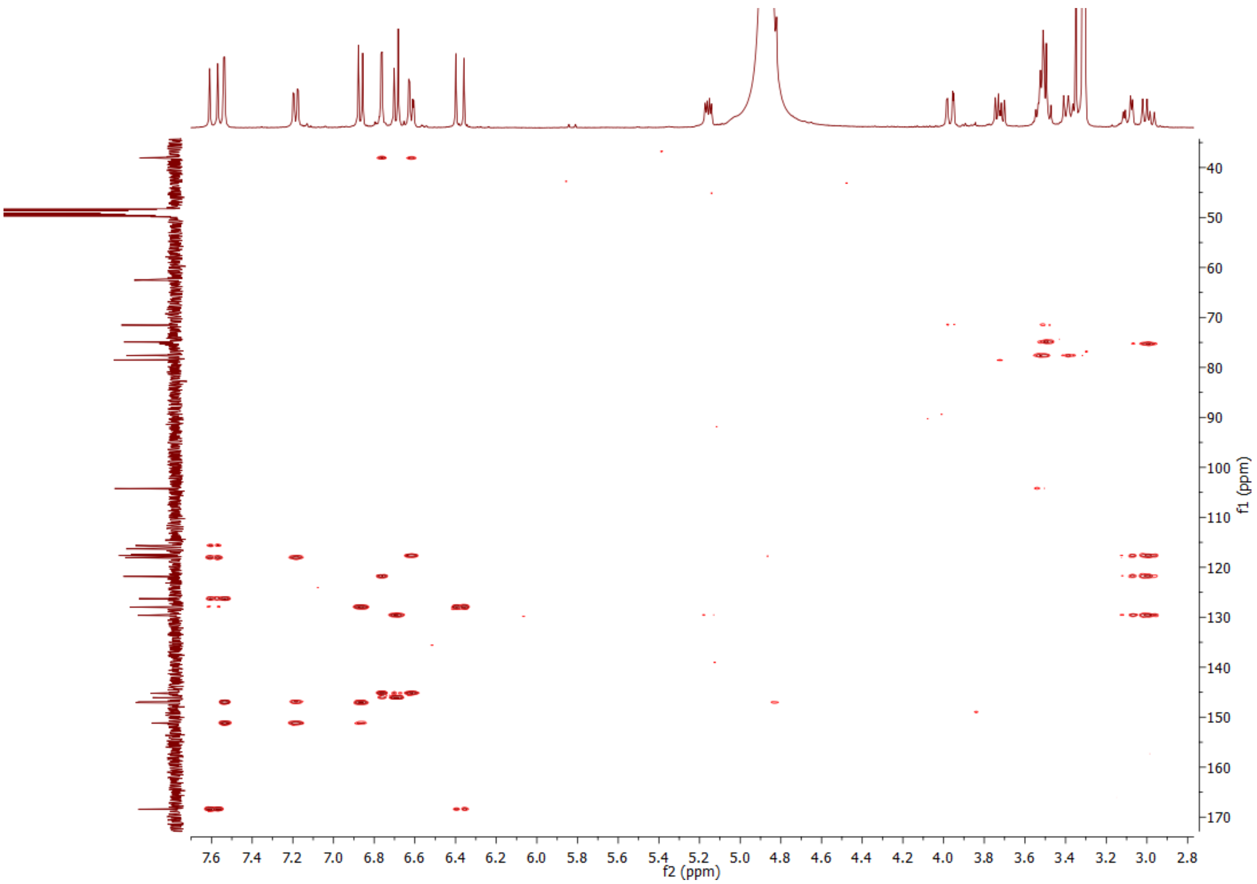

Figure S11. ¹H–¹³C HMBC (Methanol-d_4, 500/125 MHz) spectrum of compound **1**.

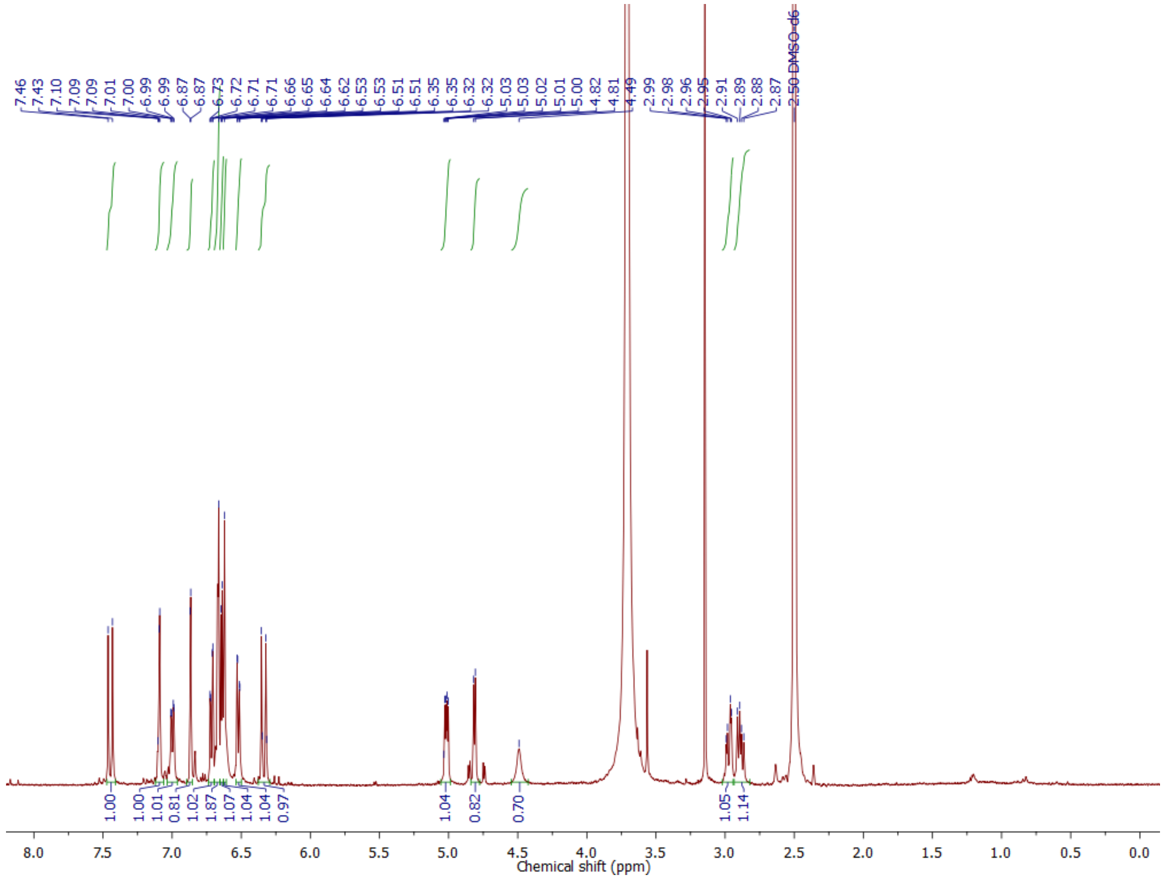

Figure S12. ^{1}H NMR (DMSO-d_6+D_2O, 500 MHz) spectrum of compound **2**.

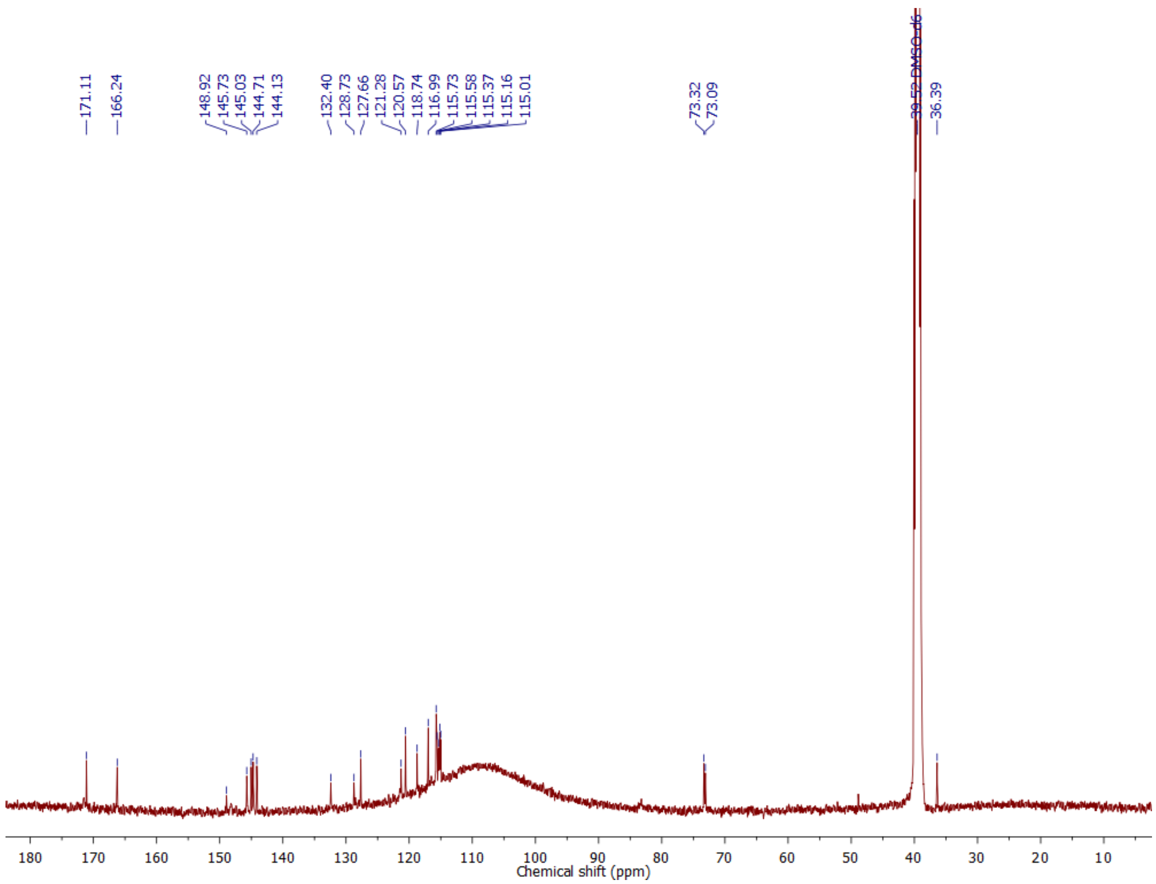

Figure S13. ^{13}C NMR (DMSO-d_6+D_2O, 125 MHz) spectrum of compound **2**.

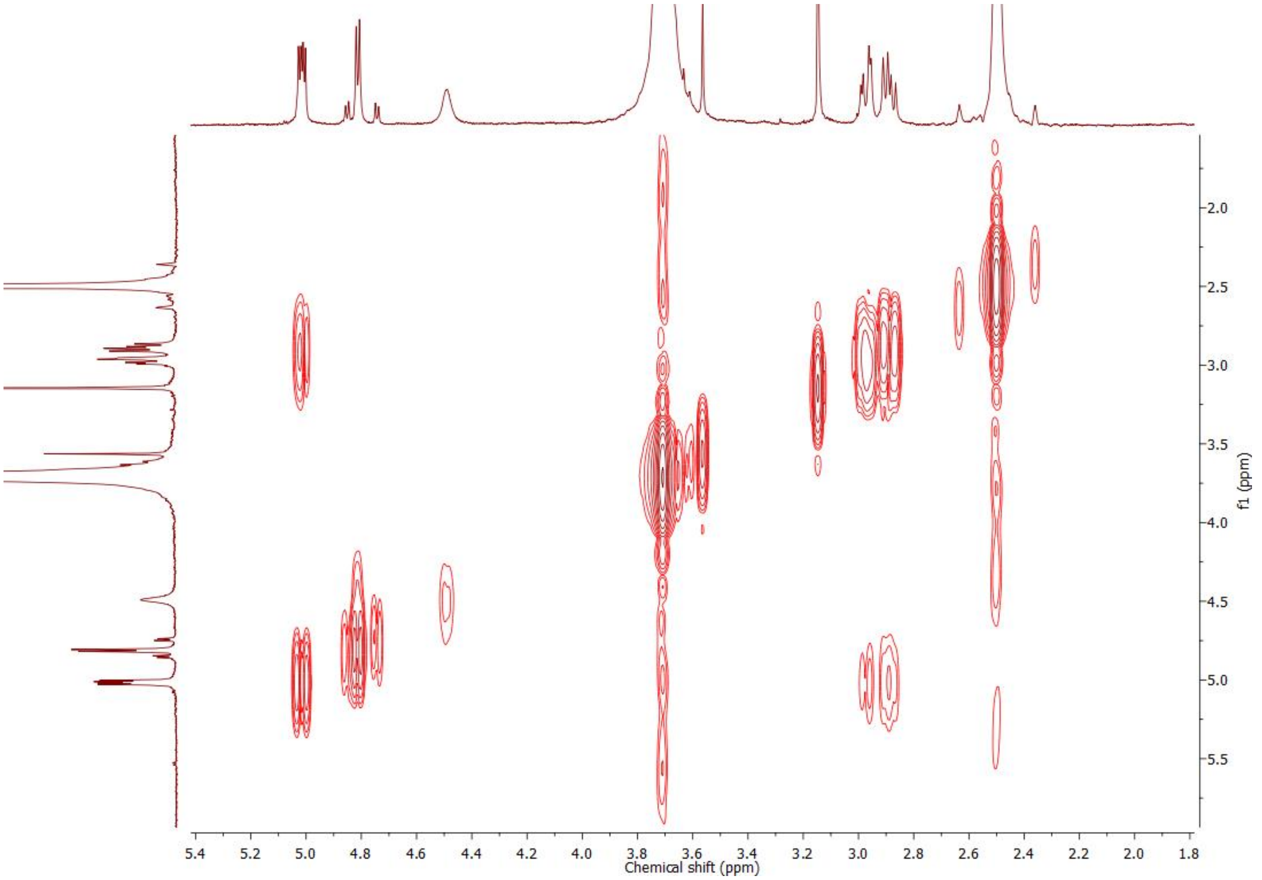

Figure S14. Band-selective ^{1}H-^{1}H COSY (DMSO-d_6+D_2O, 500 MHz) spectrum of compound **2** for aliphatic region.

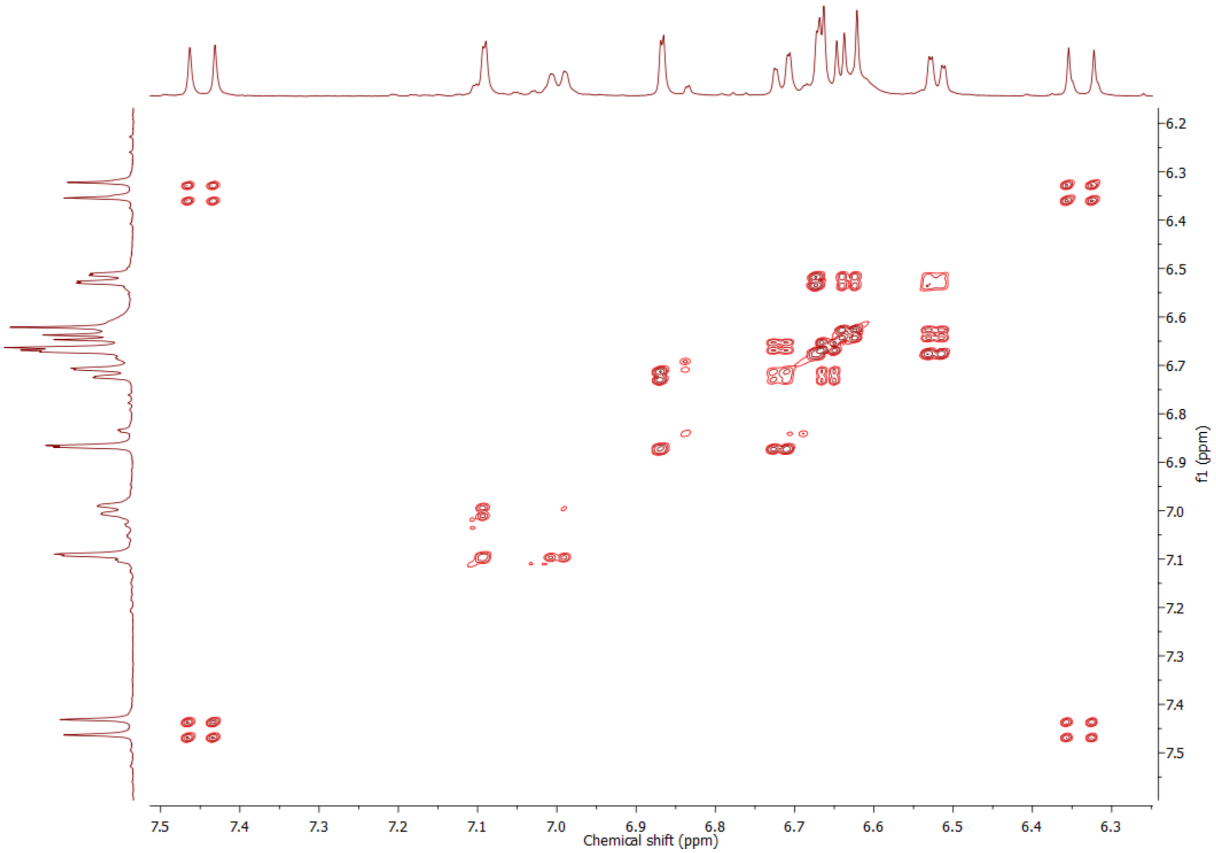

Figure S15. Band-selective ^{1}H-^{1}H COSY (DMSO-d_6+D_2O, 500 MHz) spectrum of compound **2** for aromatic region.

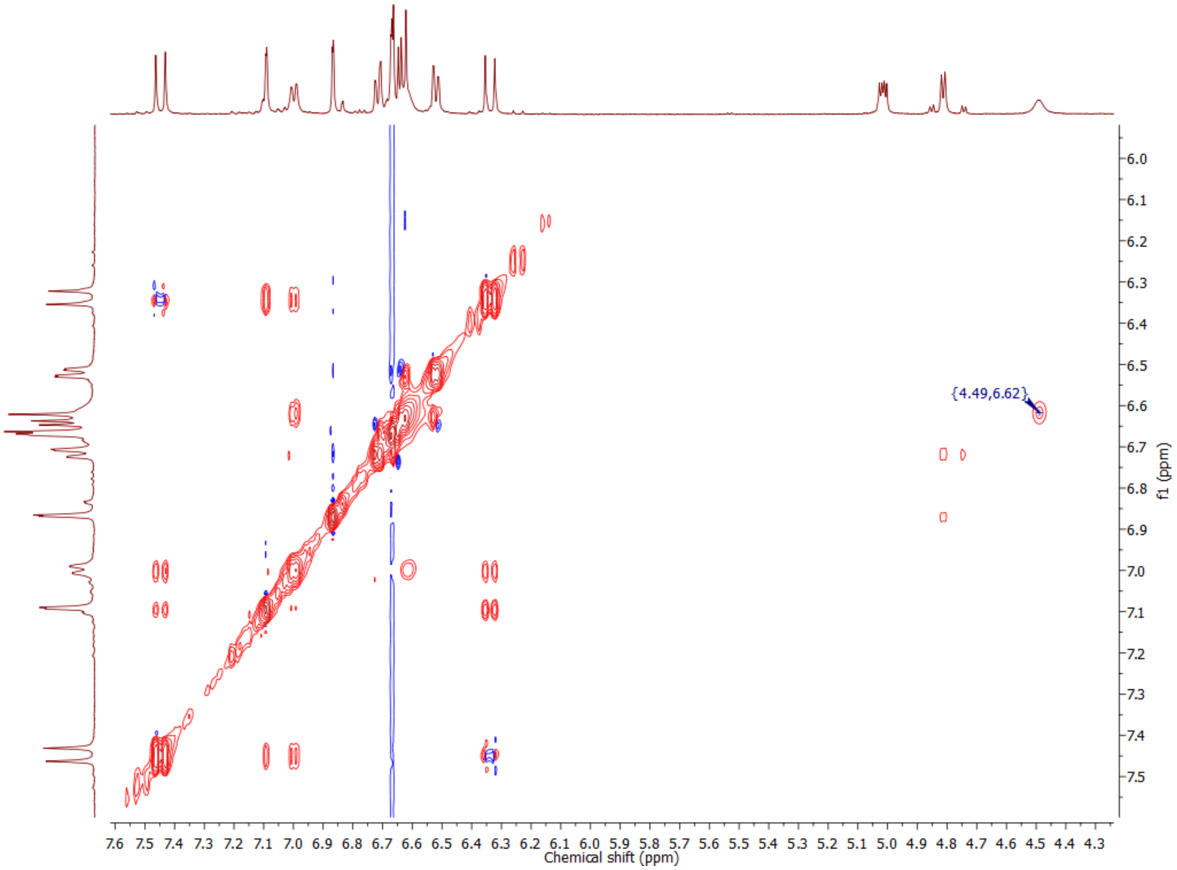

Figure S16. ^1H-^1H NOESY (DMSO-d_6+D_2O, 500 MHz) spectrum of compound **2.** The labelled cross-peak confirms the connecting position between rosmarinic acid and (3,4-dihydroxyphenyl)glyceric acid moieties.

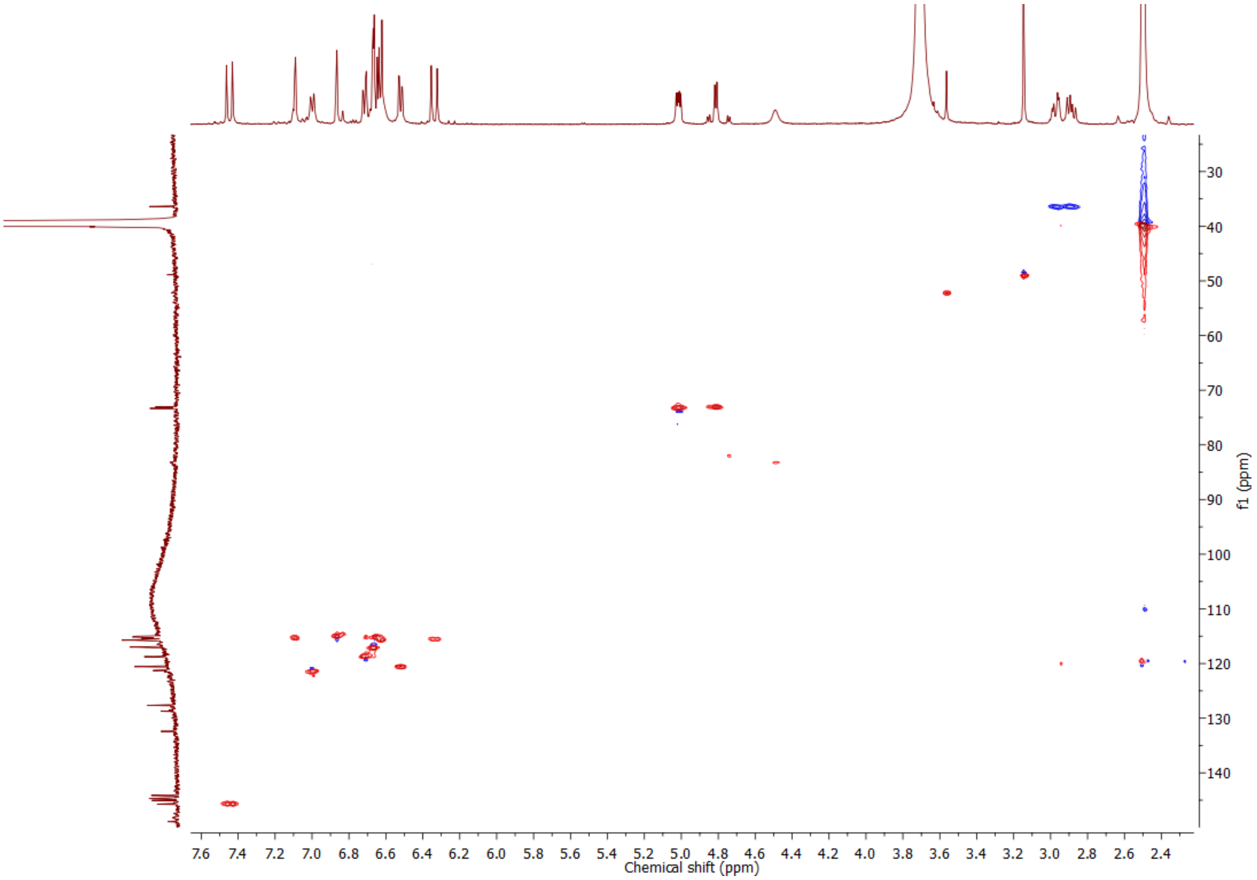

Figure S17. ^{1}H–^{13}C HSQC (DMSO-d_6+D_2O, 500/125 MHz) spectrum of compound **2**.

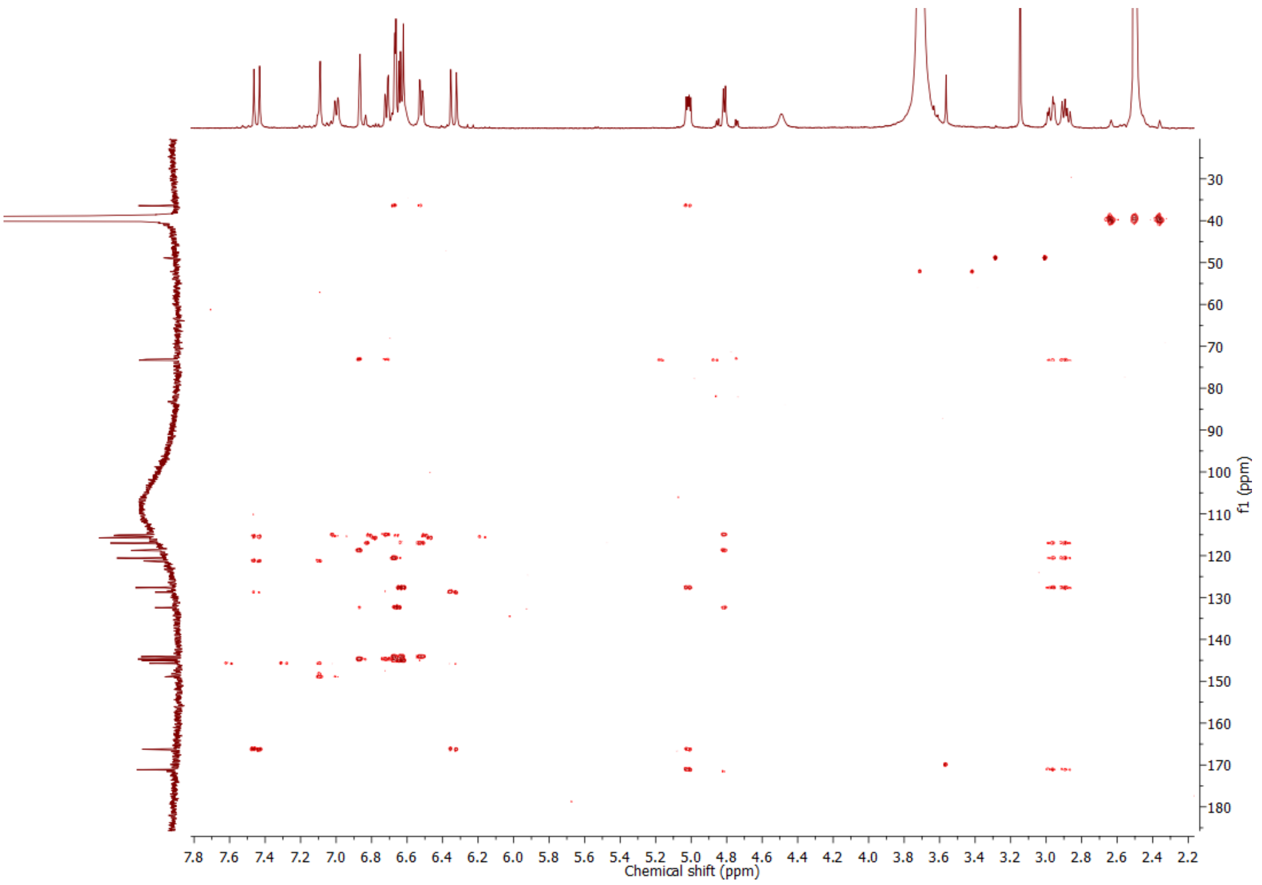

Figure S18. ^{1}H–^{13}C HMBC (DMSO-d_6+D_2O, 500/125 MHz) spectrum of compound **2**.

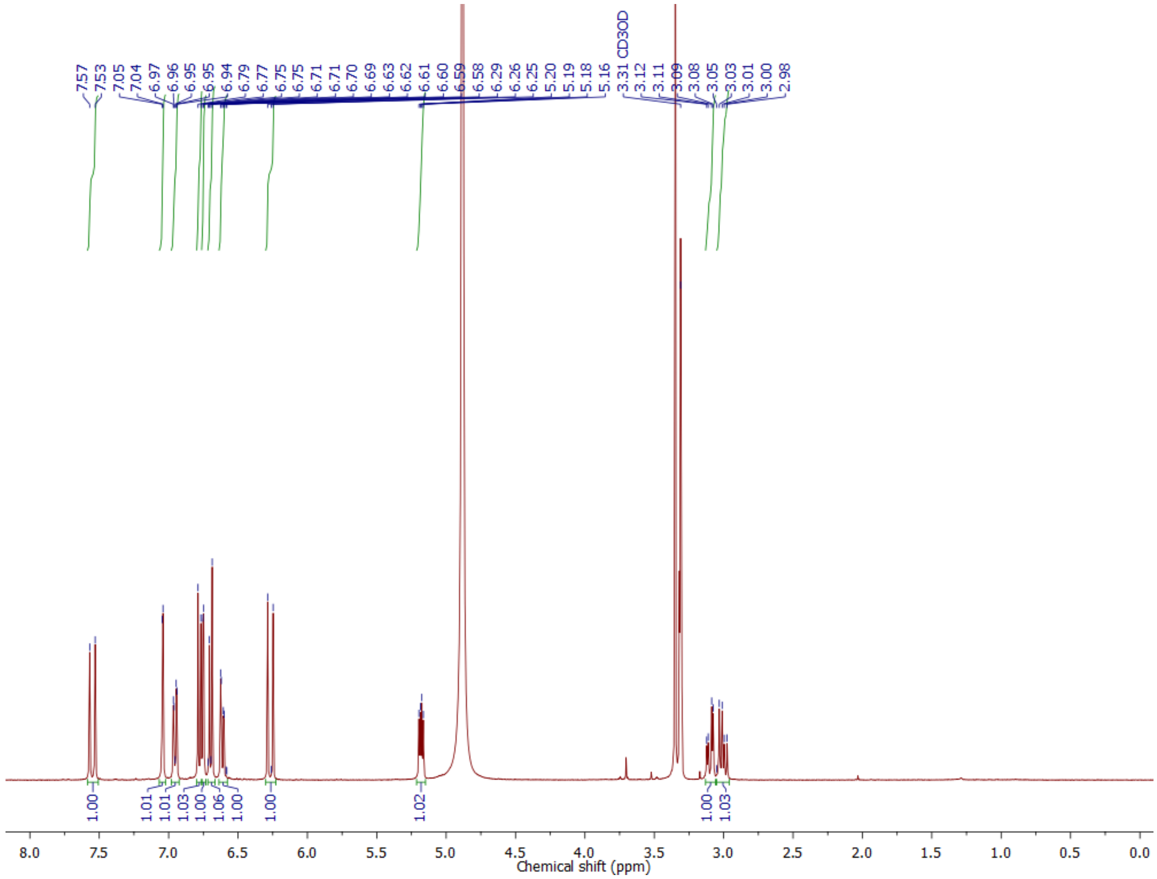

Figure S19. ^{1}H NMR (Methanol-*d*$_4$, 500 MHz) spectrum of compound **3**.

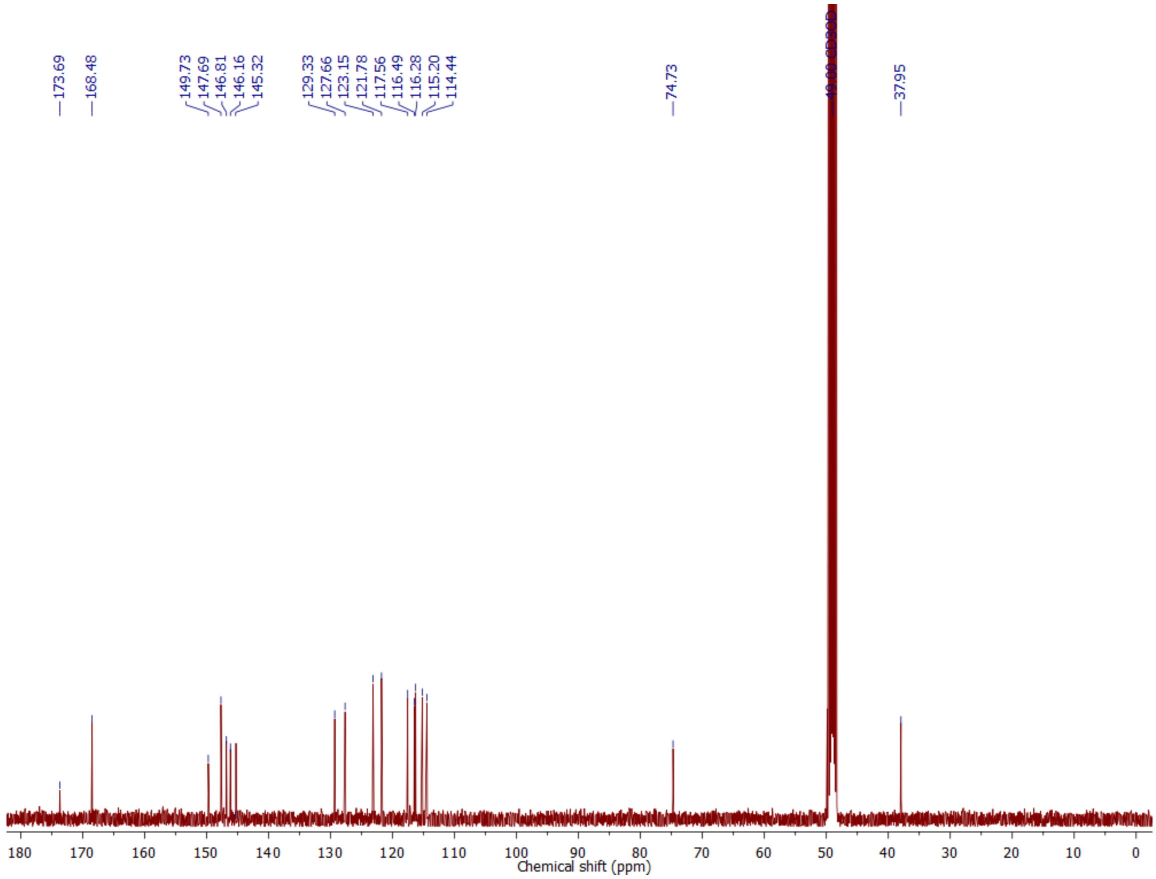

Figure S20. ^{13}C NMR (Methanol-d_4, 125 MHz) spectrum of compound **3**.

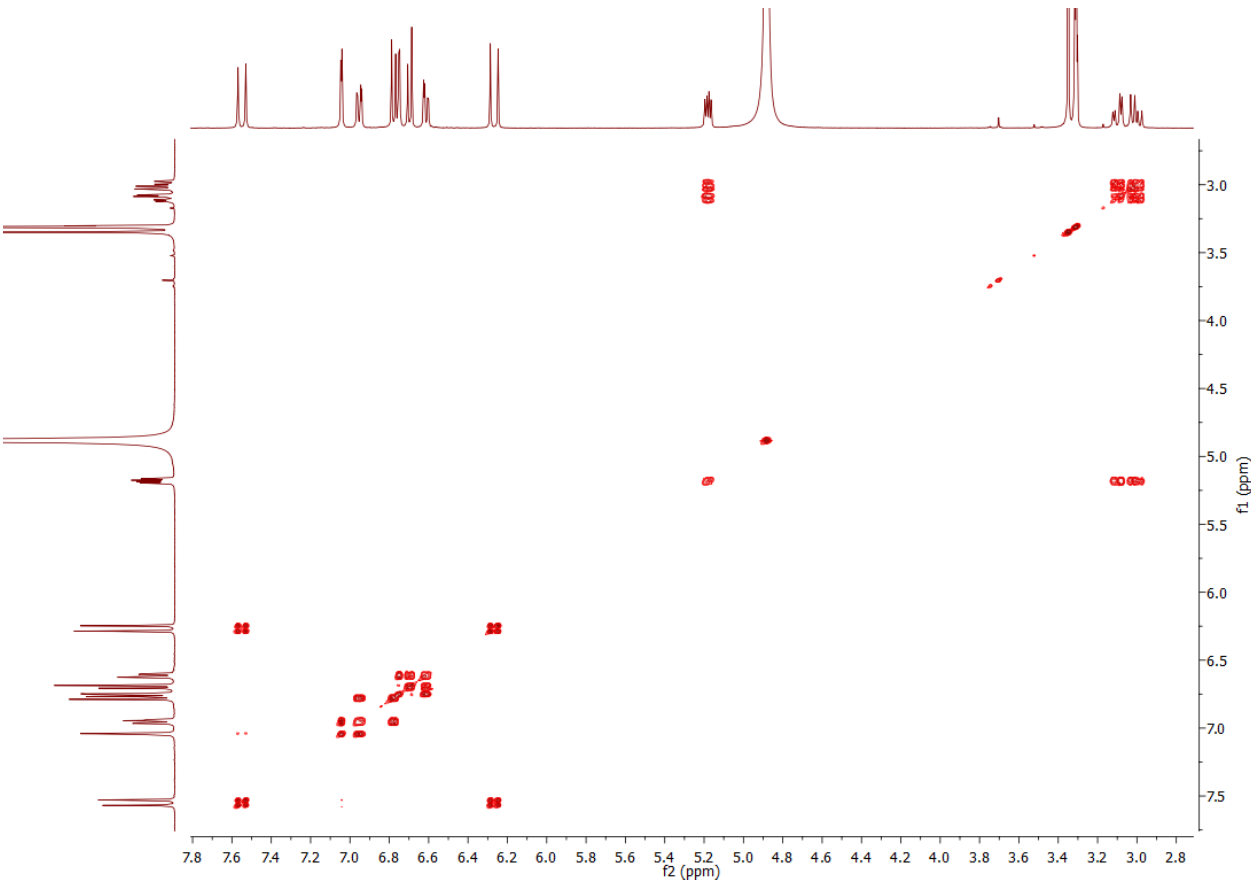

Figure S21. ^1H-^1H COSY (Methanol-d_4, 500 MHz) spectrum of compound **3**.

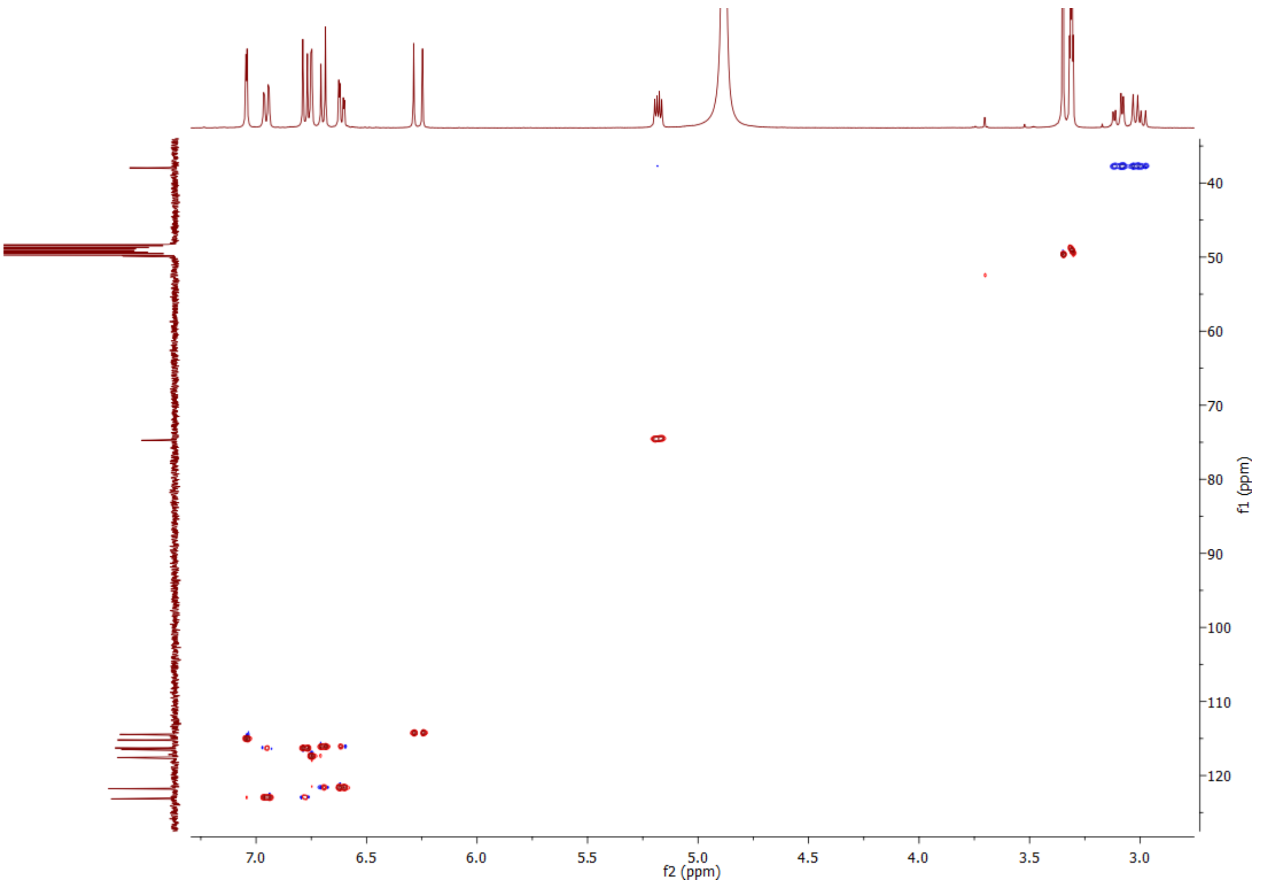

Figure S22. ^{1}H–^{13}C HSQC (Methanol-*d*$_4$, 500/125 MHz) spectrum of compound **3**.

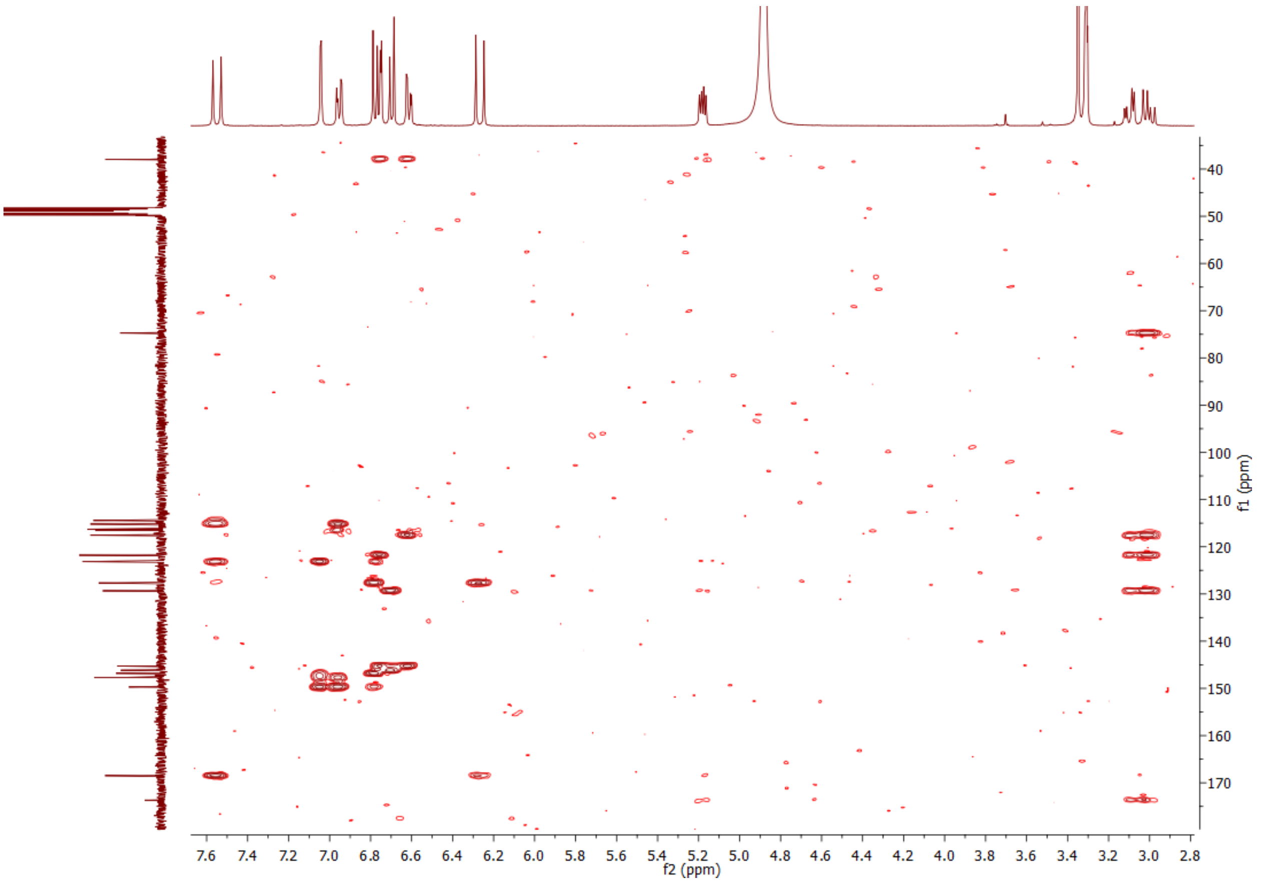

Figure S23. ^{1}H–^{13}C HMBC (Methanol-d_4, 500/125 MHz) spectrum of compound **3**.

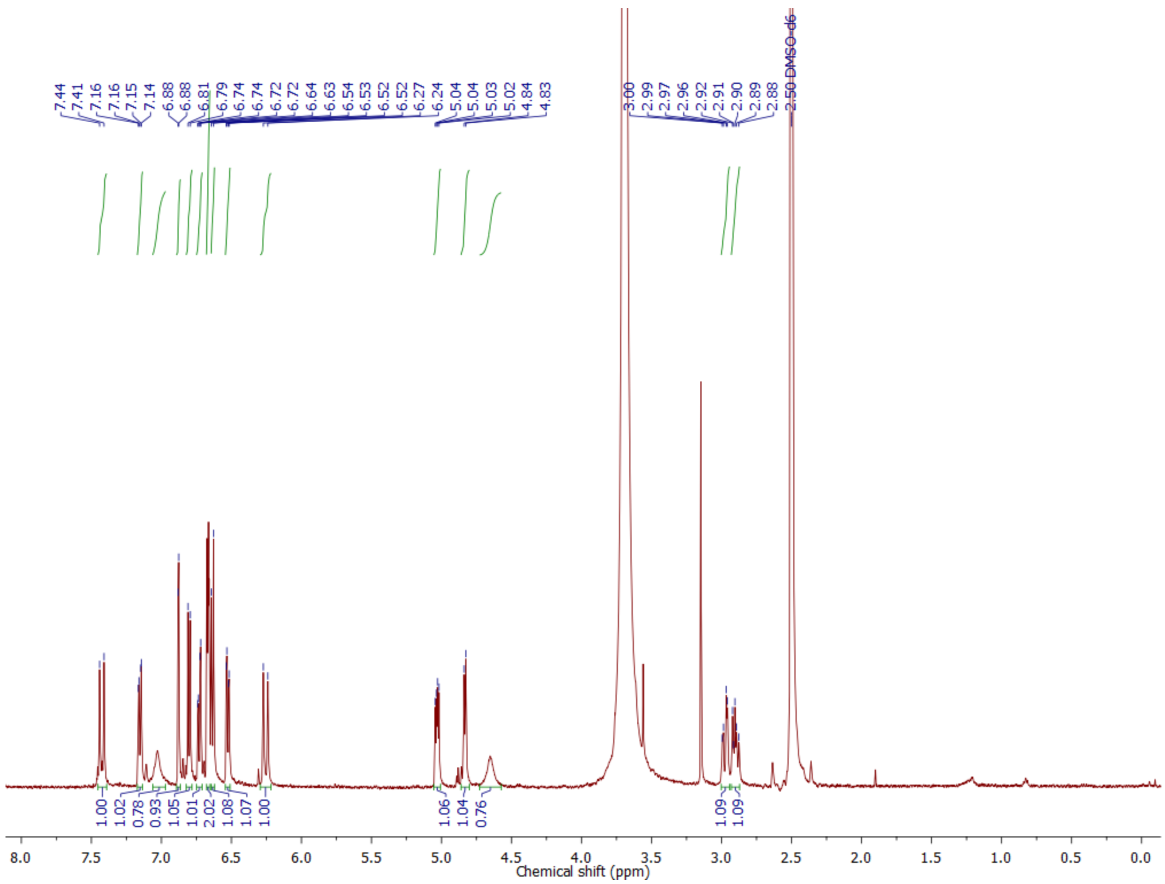

Figure S24. ^{1}H NMR (DMSO-d_6+D_2O, 500 MHz) spectrum of compound **4**.

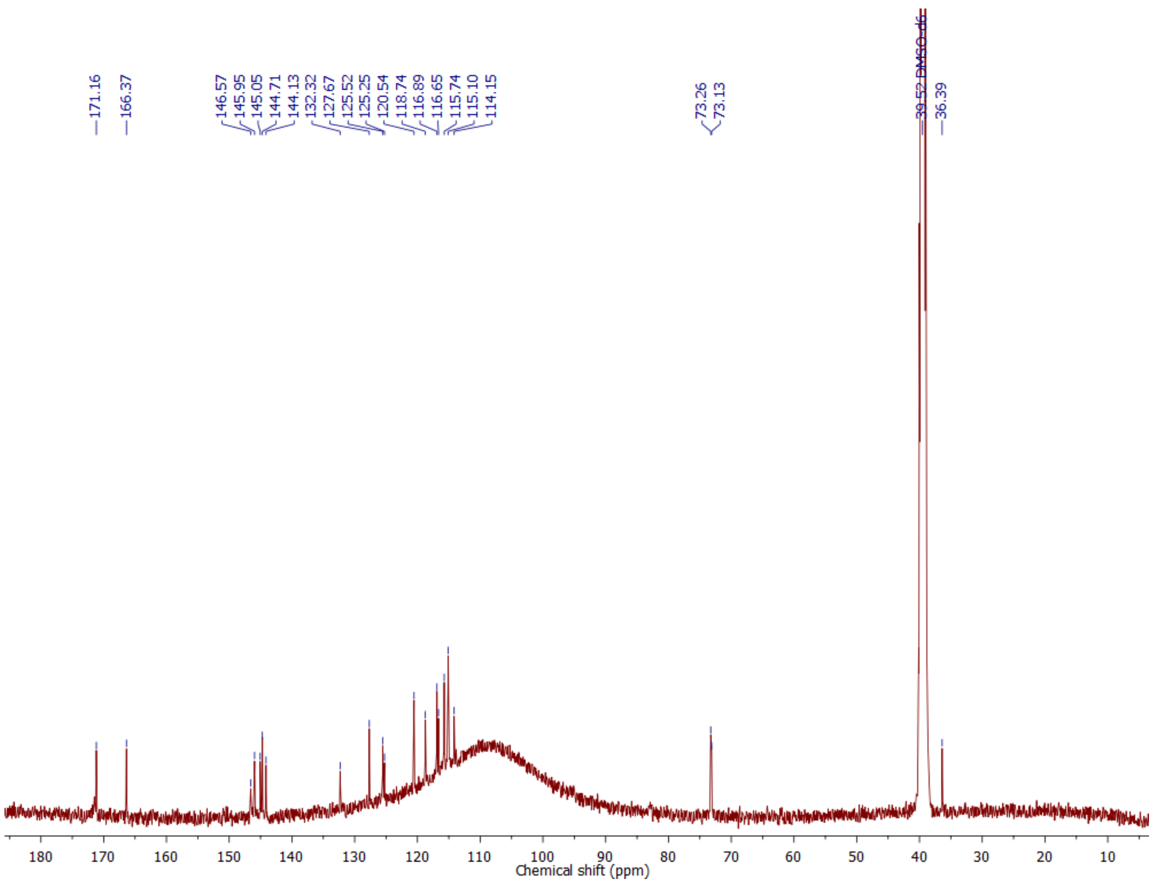

Figure S25. ^{13}C NMR (DMSO-d_6+D_2O, 125 MHz) spectrum of compound **4**.

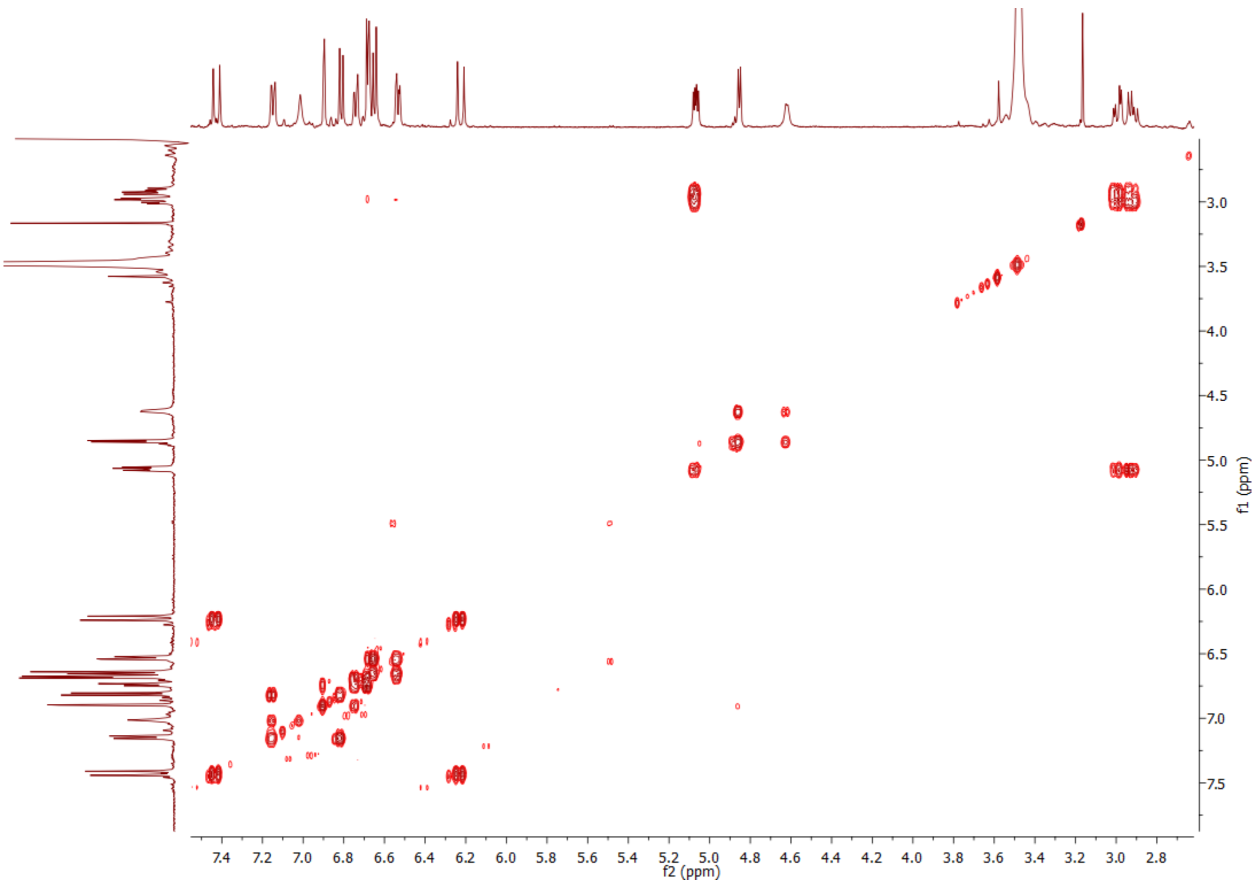

Figure S26. ^1H-^1H COSY (DMSO-d_6+D_2O, 500 MHz) spectrum of compound **4**.

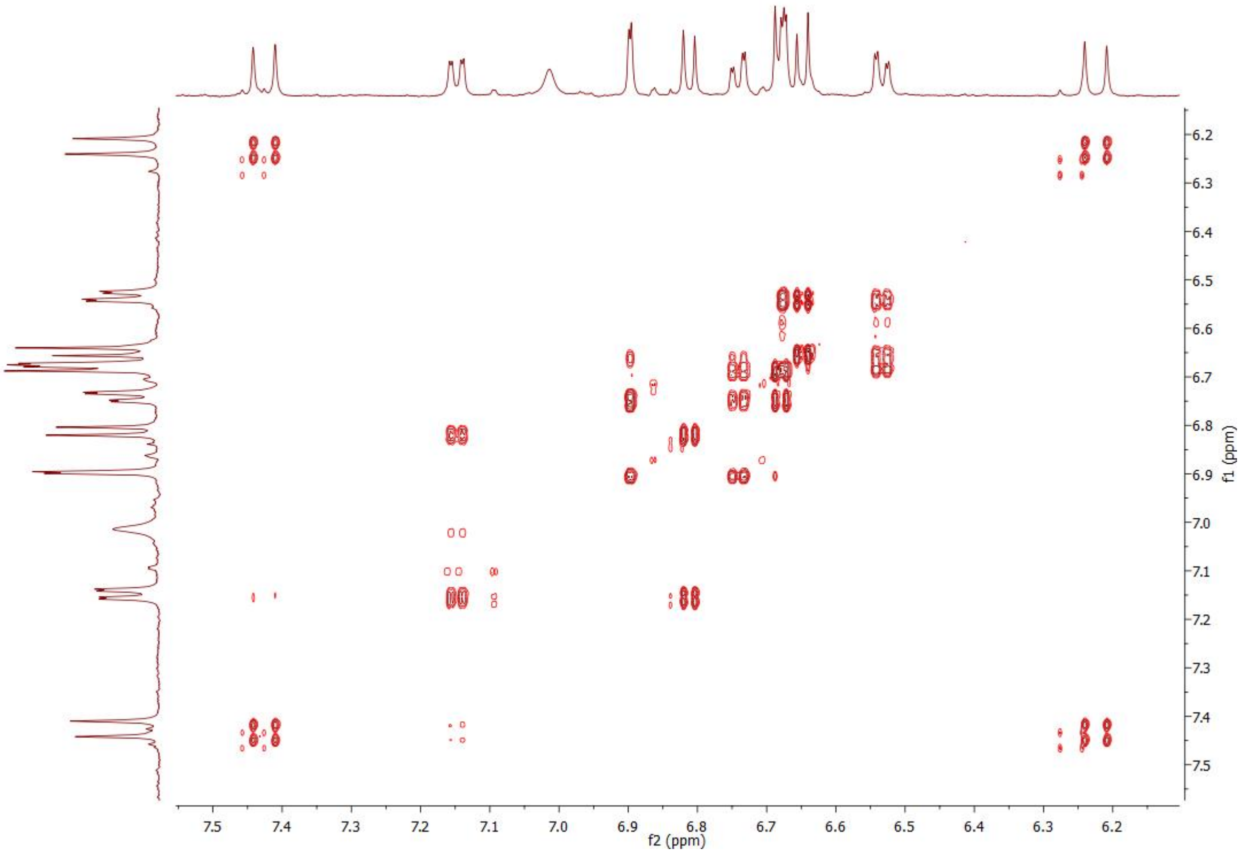

Figure S27. Band-selective ^{1}H-^{1}H COSY (DMSO-d_6+D_2O, 500 MHz) spectrum of compound **4** for aromatic region.

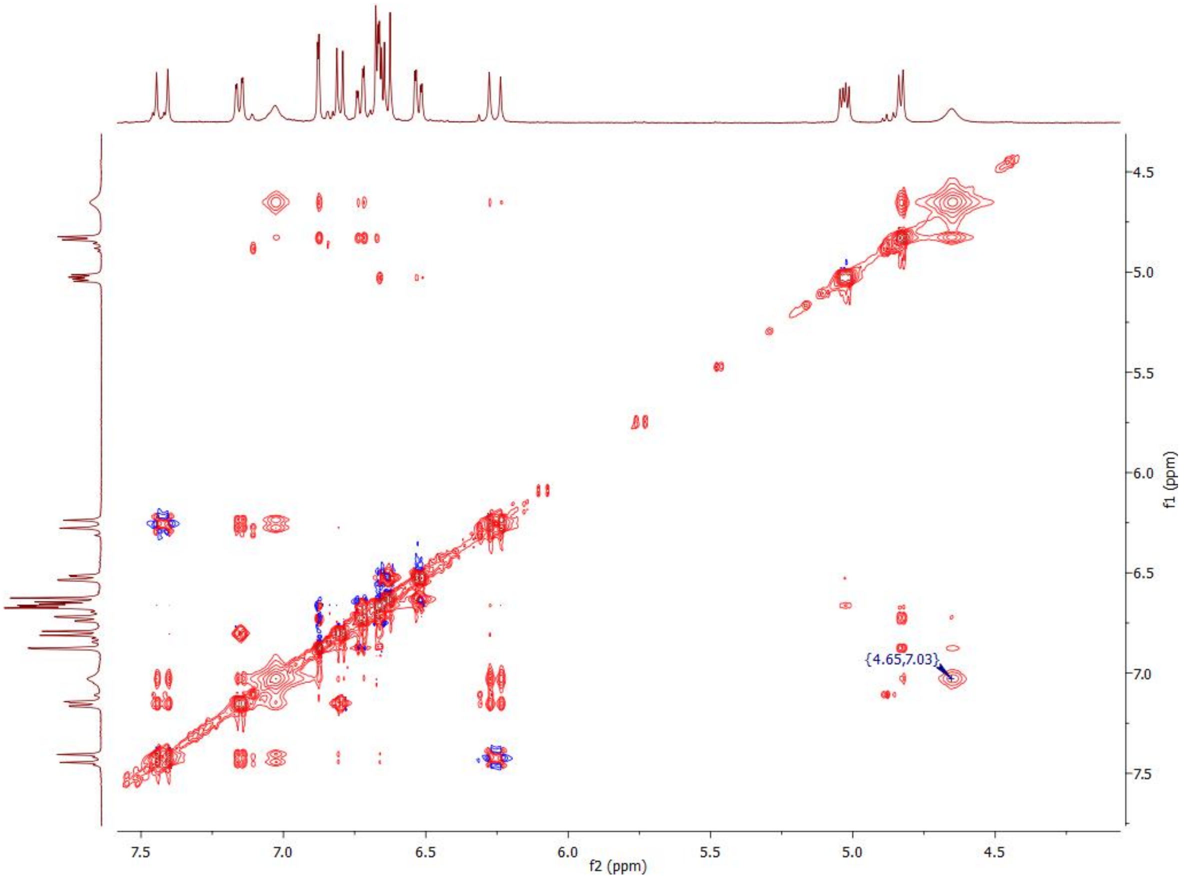

Figure S28. ^{1}H-^{1}H NOESY (DMSO-d_6+D_2O, 500 MHz) spectrum of compound **4**. The labelled cross-peak confirms the connecting position between rosmarinic acid and (3,4-dihydroxyphenyl)glyceric acid moieties.

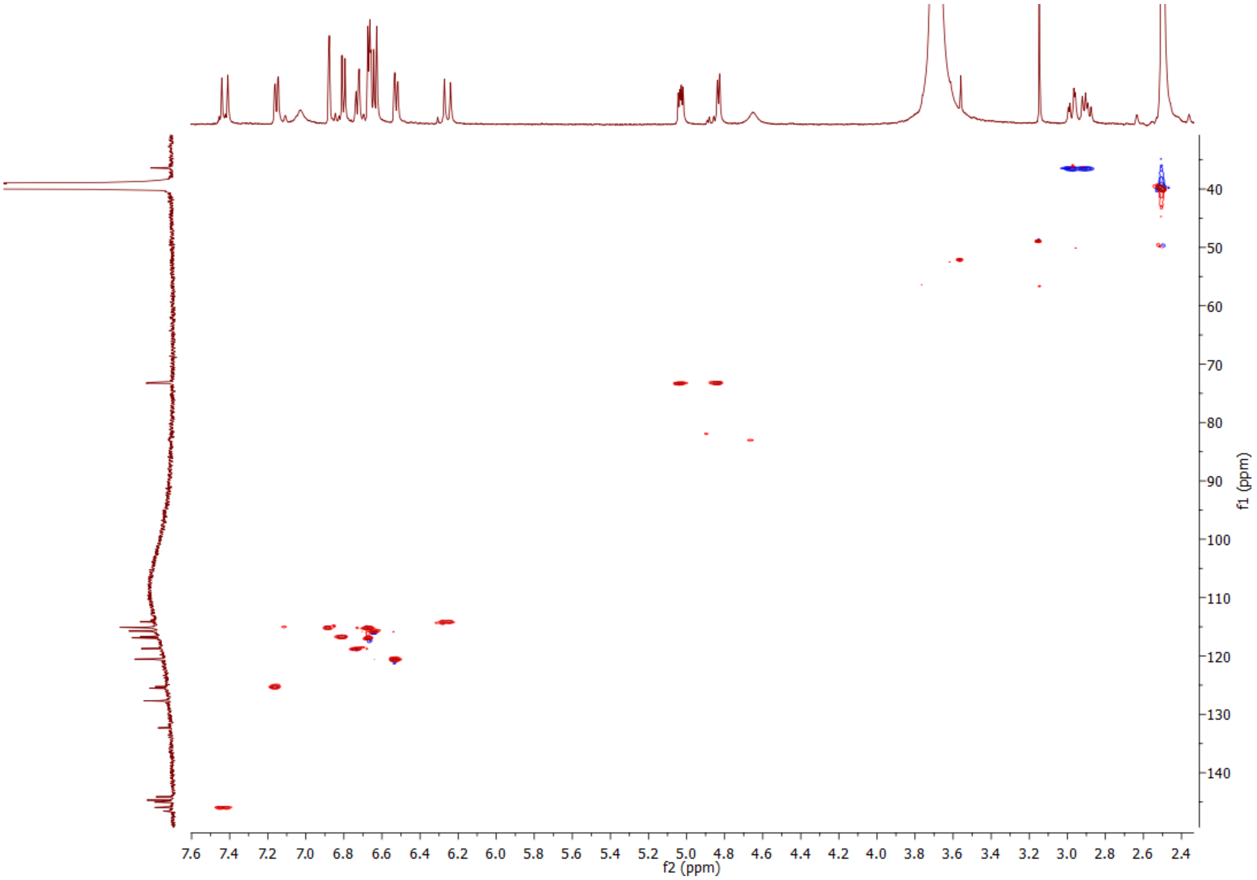

Figure S29. ^{1}H–^{13}C HSQC (DMSO-d_6+D_2O, 500/125 MHz) spectrum of compound **4**.

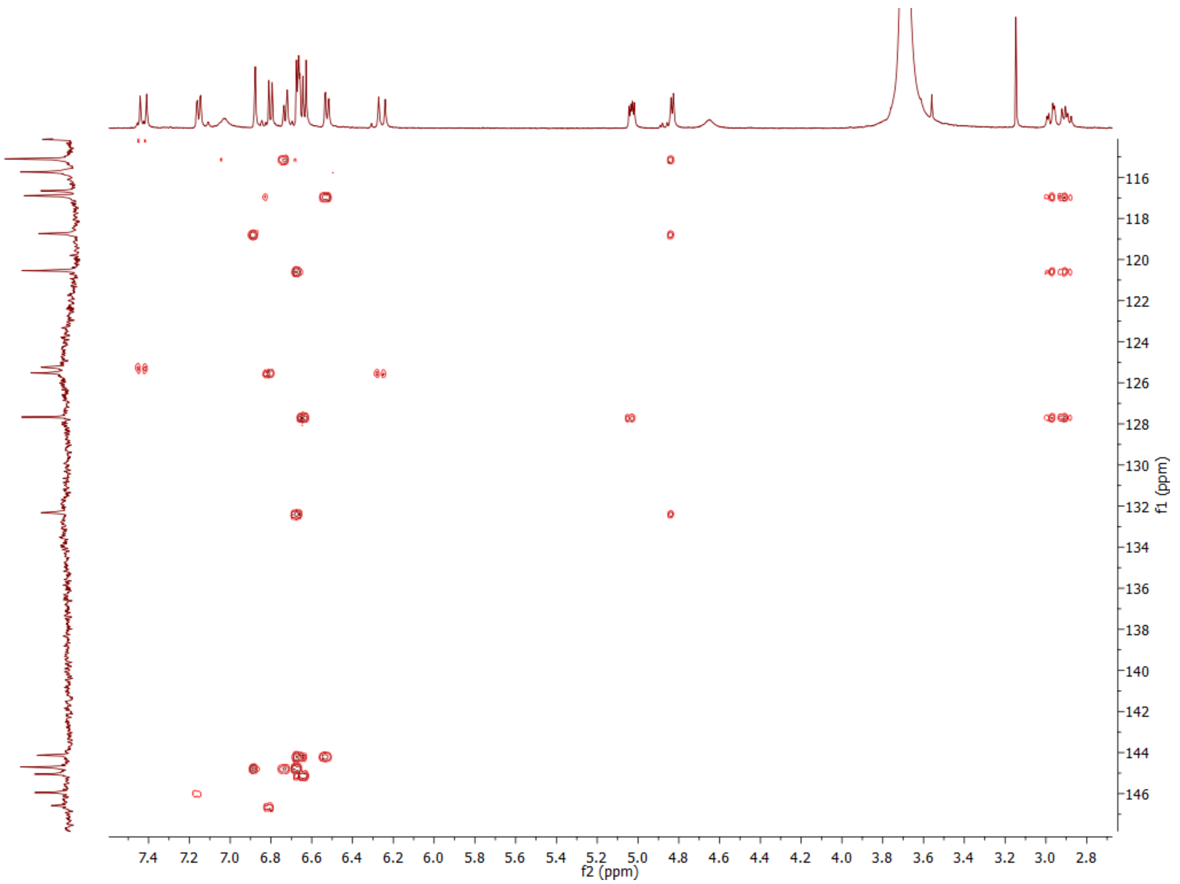

Figure S30. Band-selective ^{1}H–^{13}C HMBC (DMSO-d_6+D_2O, 500/125 MHz) spectrum of compound **4**.

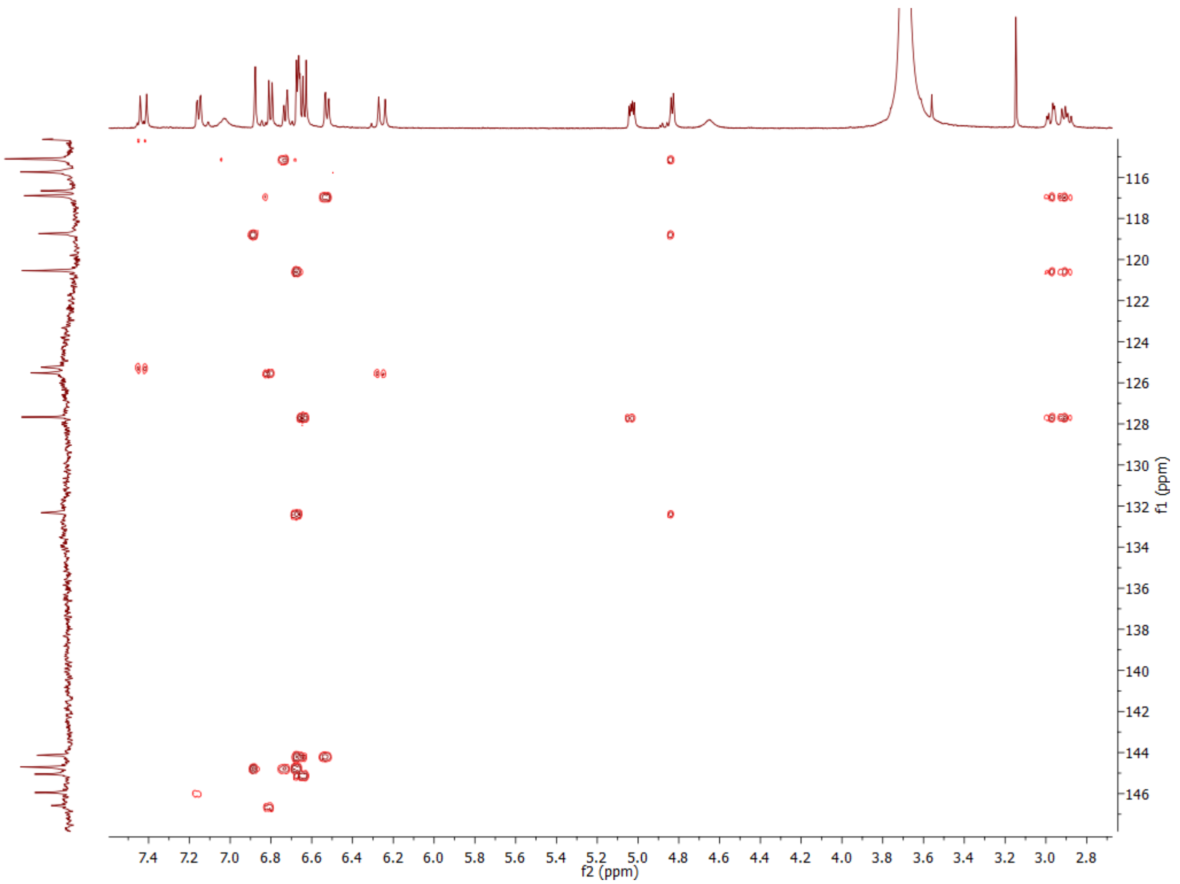

Figure S31. Band-selective ^{1}H–^{13}C HMBC (DMSO-d_6+D_2O, 500/125 MHz) spectrum of compound **4**.

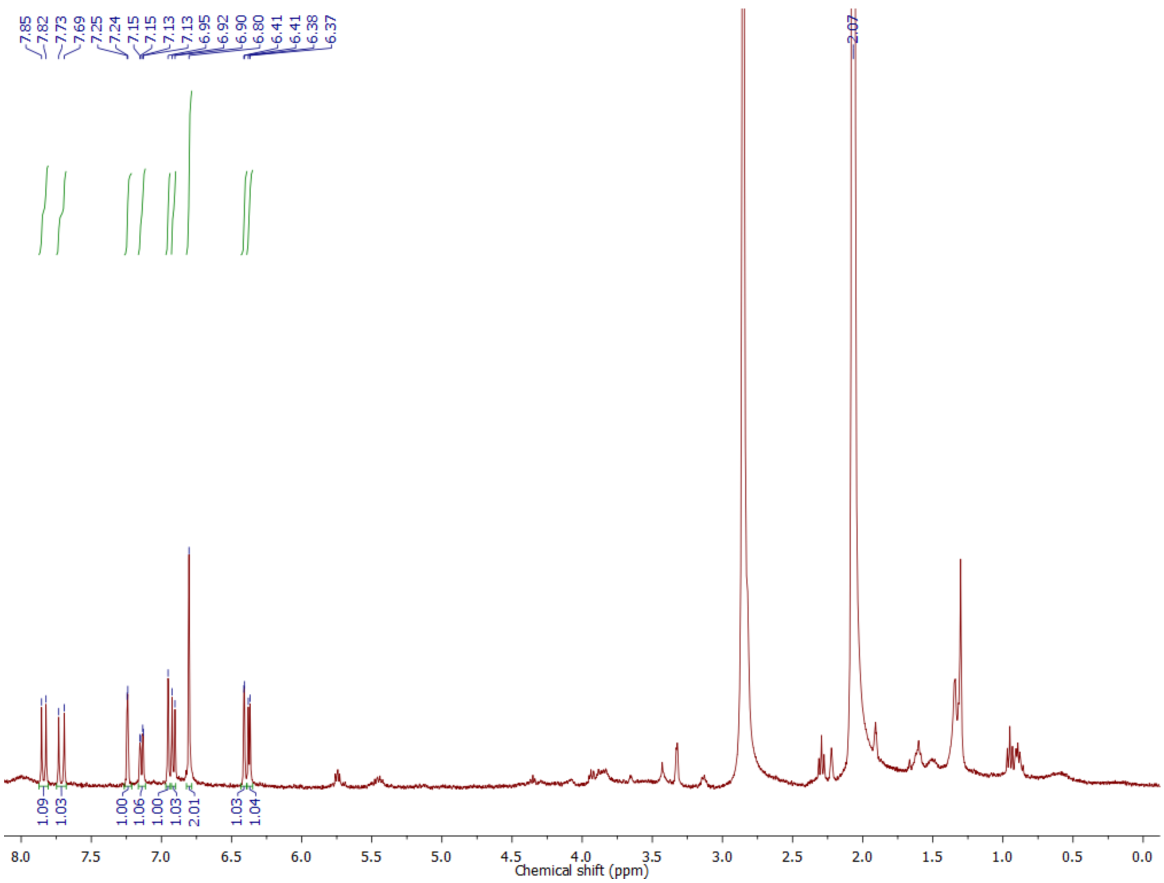

Figure S32. ^{1}H NMR (Acetone-d_6, 500 MHz) spectrum of compound **5**.

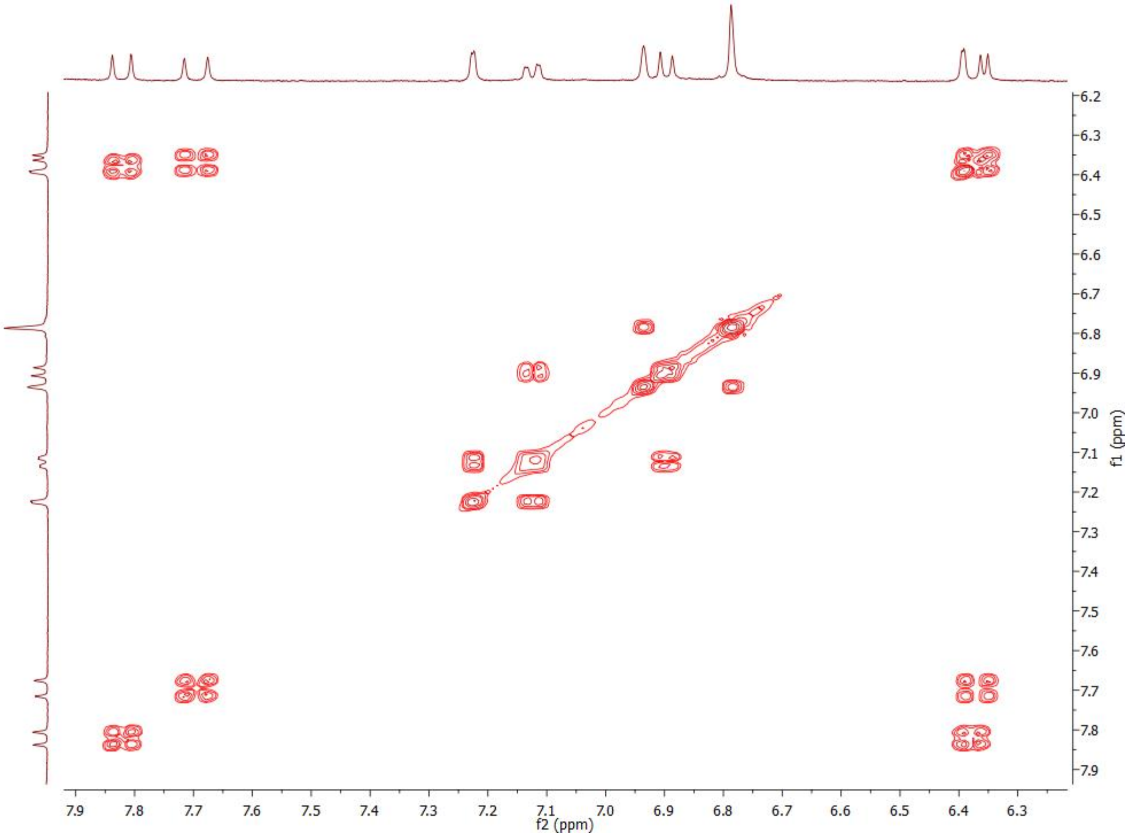

Figure S33. ^1H-^1H COSY (Acetone-d_6, 500 MHz) spectrum of compound **5**.

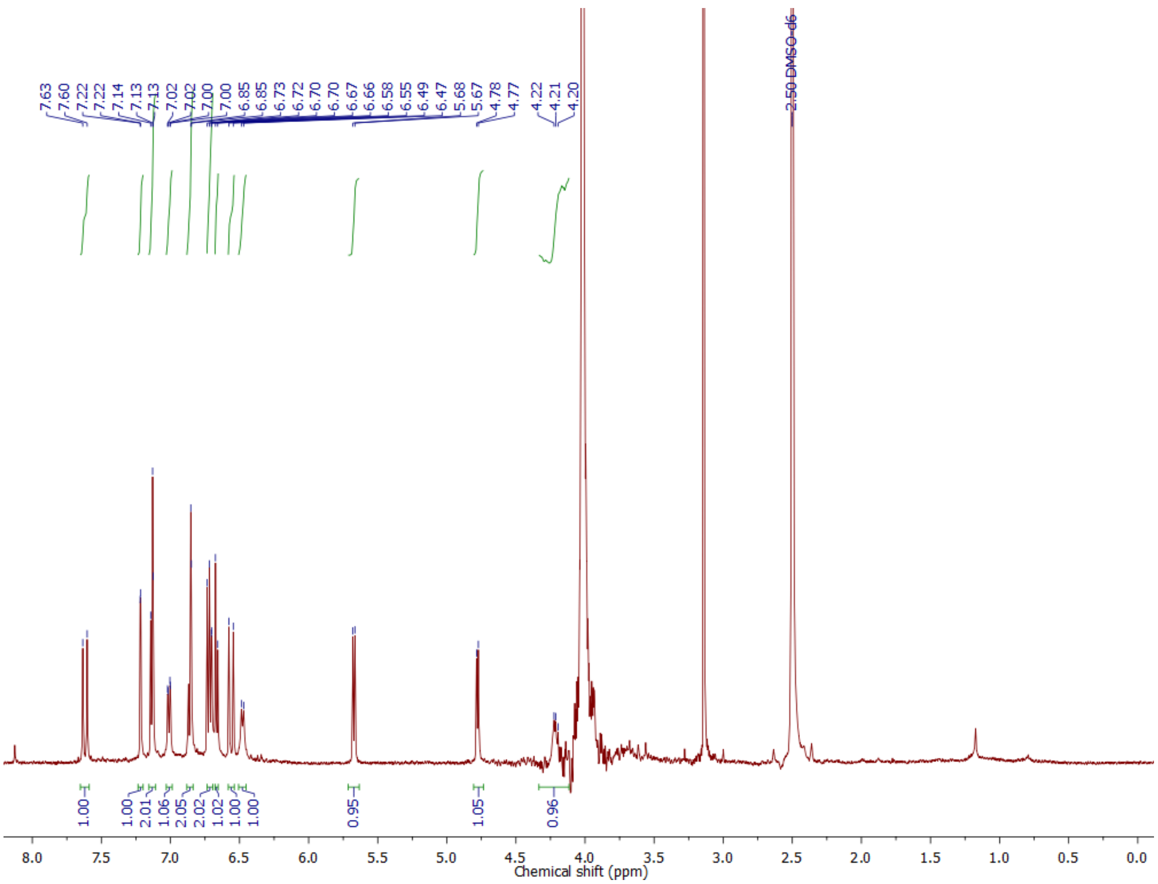

Figure S34. ^{1}H NMR (DMSO-d_6+D_2O, 500 MHz) spectrum of compound **6.**

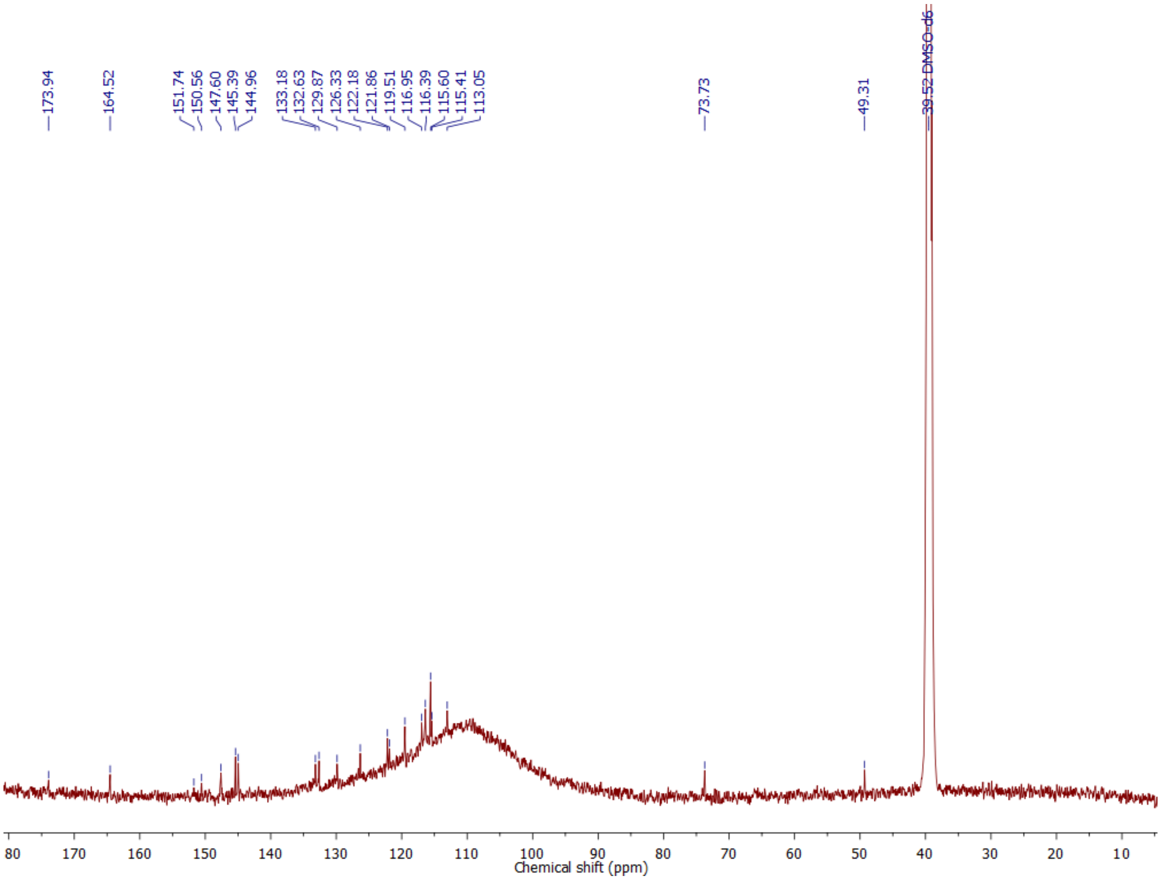

Figure S35. ^{13}C NMR (DMSO-d_6+D_2O, 125 MHz) spectrum of compound **6**.

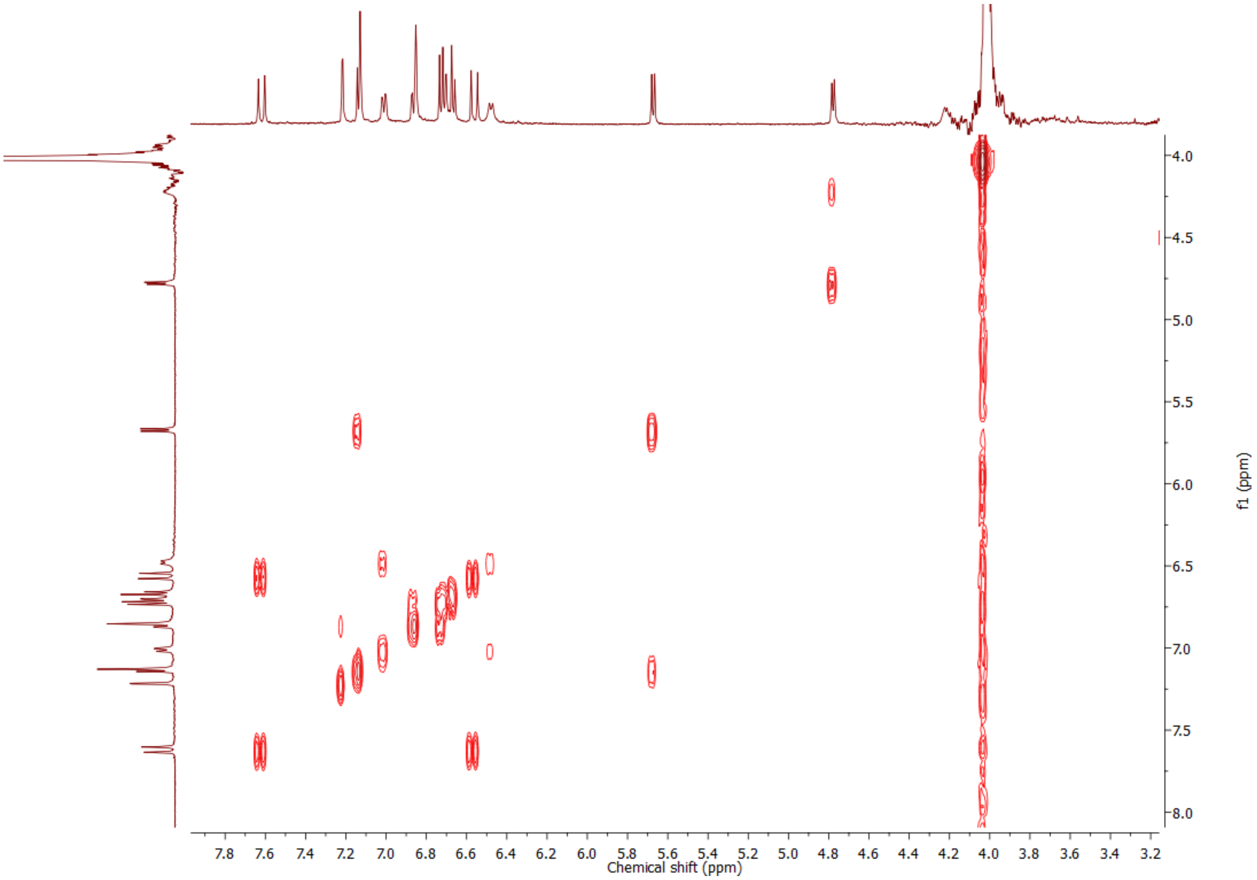

Figure S36. ^{1}H-^{1}H COSY (DMSO-d_6+D_2O, 500 MHz) spectrum of compound **6**.

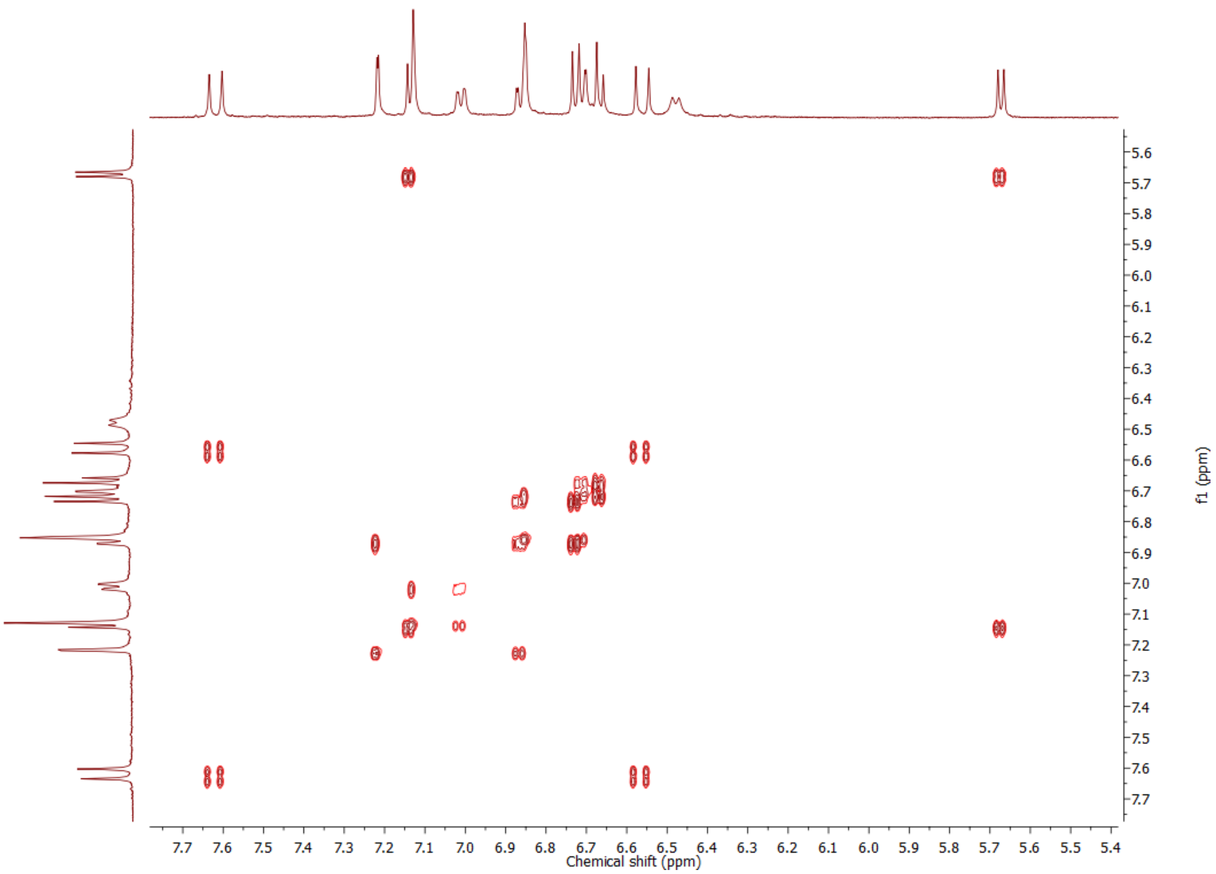

Figure S37. Band-selective ^{1}H-^{1}H COSY (DMSO-d_6+D_2O, 500 MHz) spectrum of compound **6**.

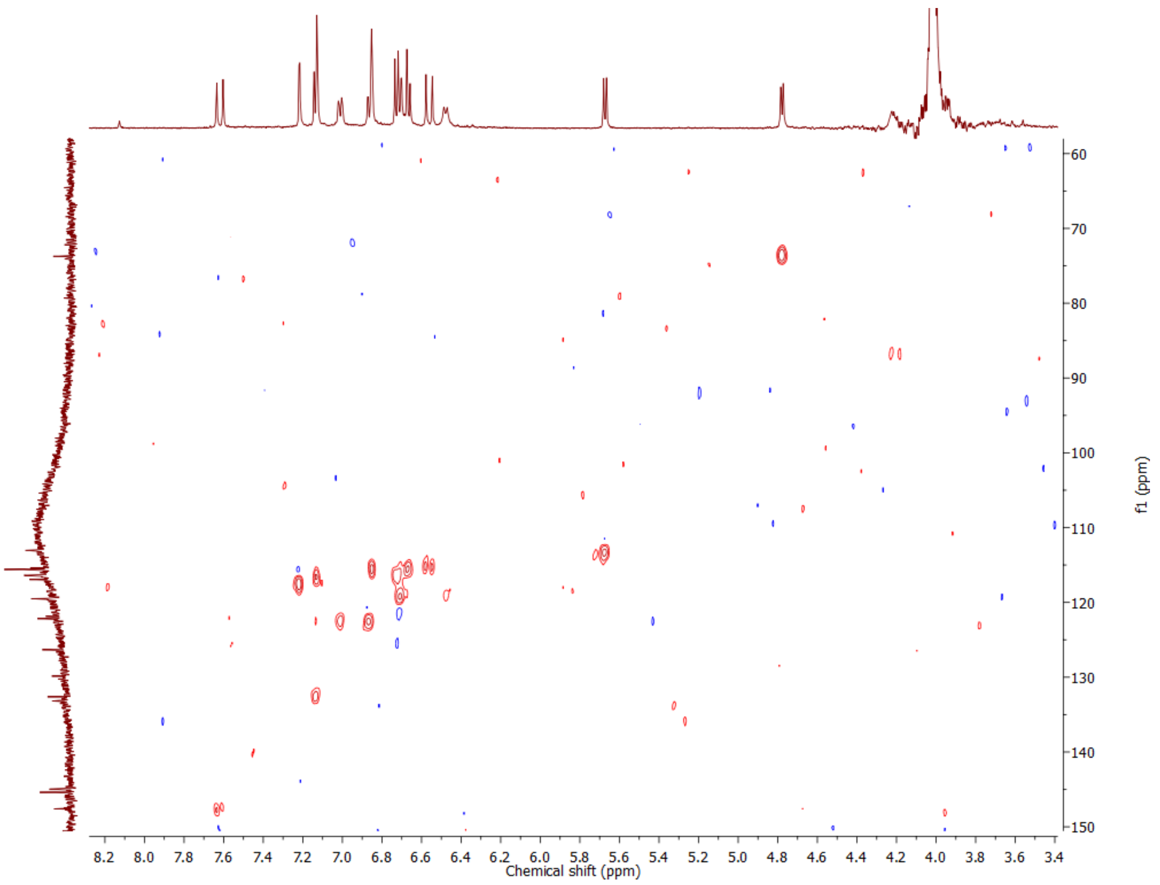

Figure S38. ^1H–^{13}C HSQC (DMSO-d_6+D_2O, 500/125 MHz) spectrum of compound **6**.

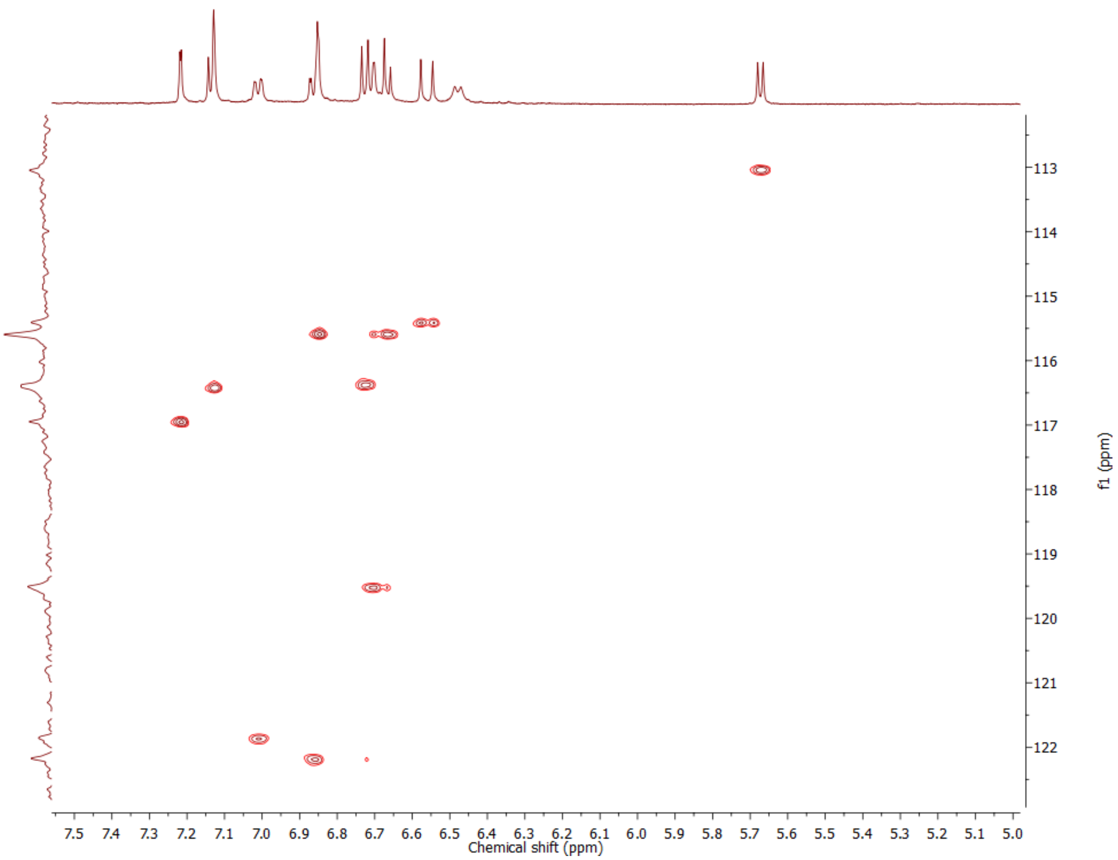

Figure S39. Band-selective ^{1}H–^{13}C HSQC (DMSO-d_6+D_2O, 500/125 MHz) spectrum of compound **6**.

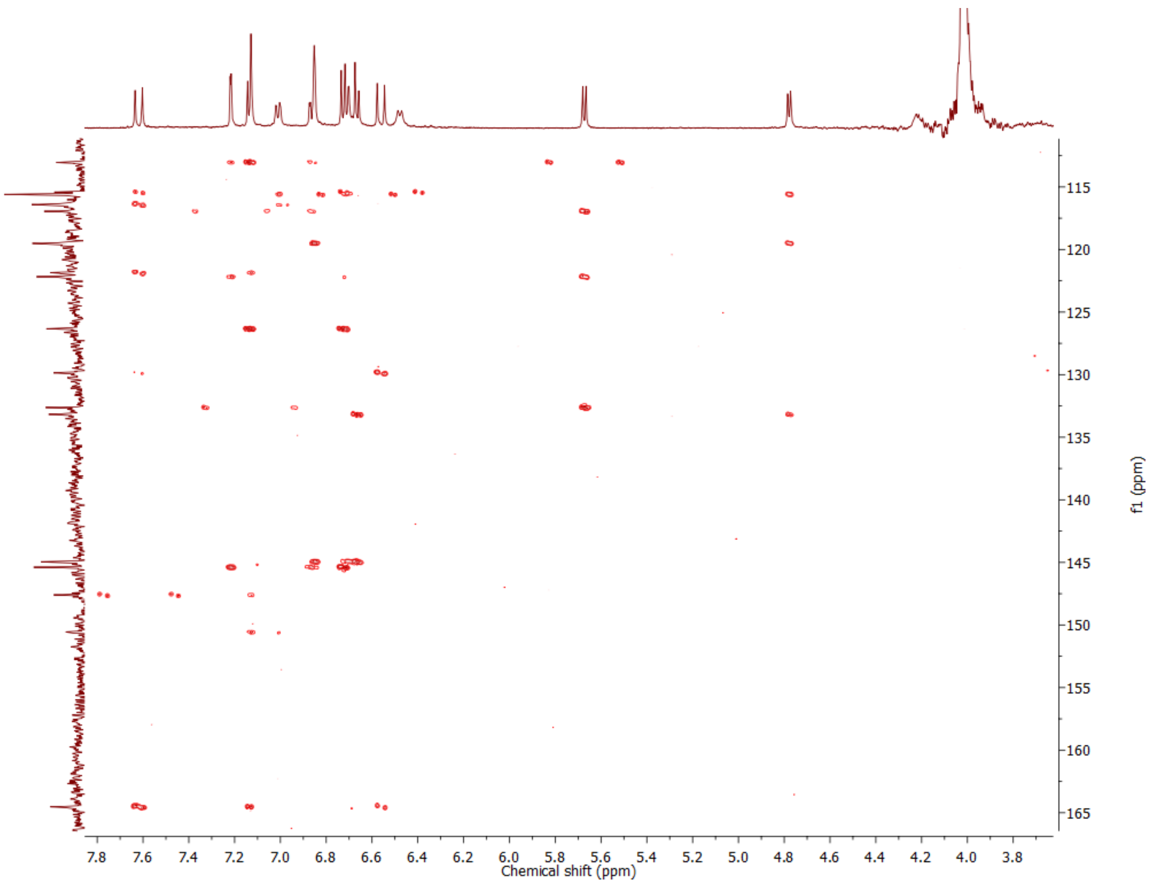

Figure S40. ^{1}H–^{13}C HMBC (DMSO-d_6+D_2O, 500/125 MHz) spectrum of compound **6**.

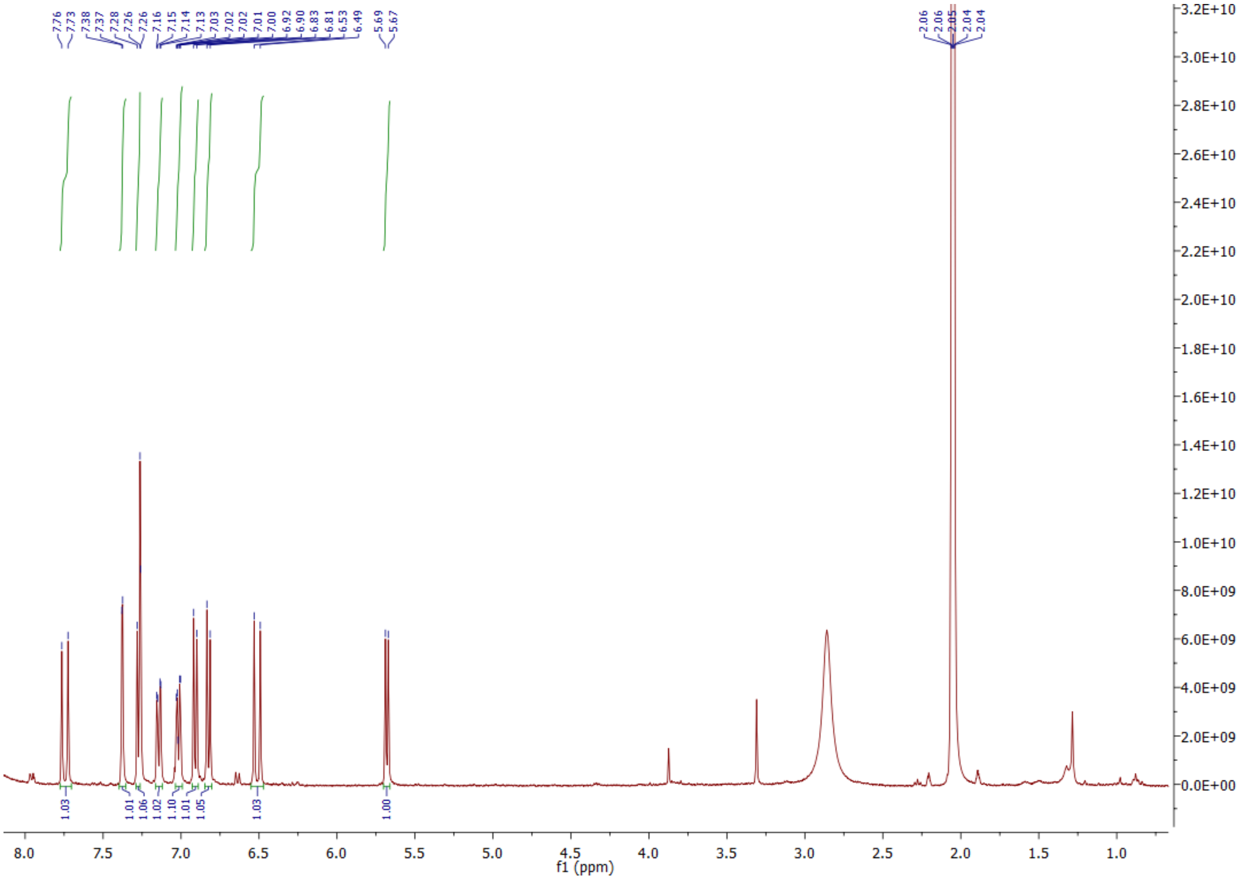

Figure S41. ^{1}H NMR (Acetone-d_6, 500 MHz) spectrum of compound **8**.

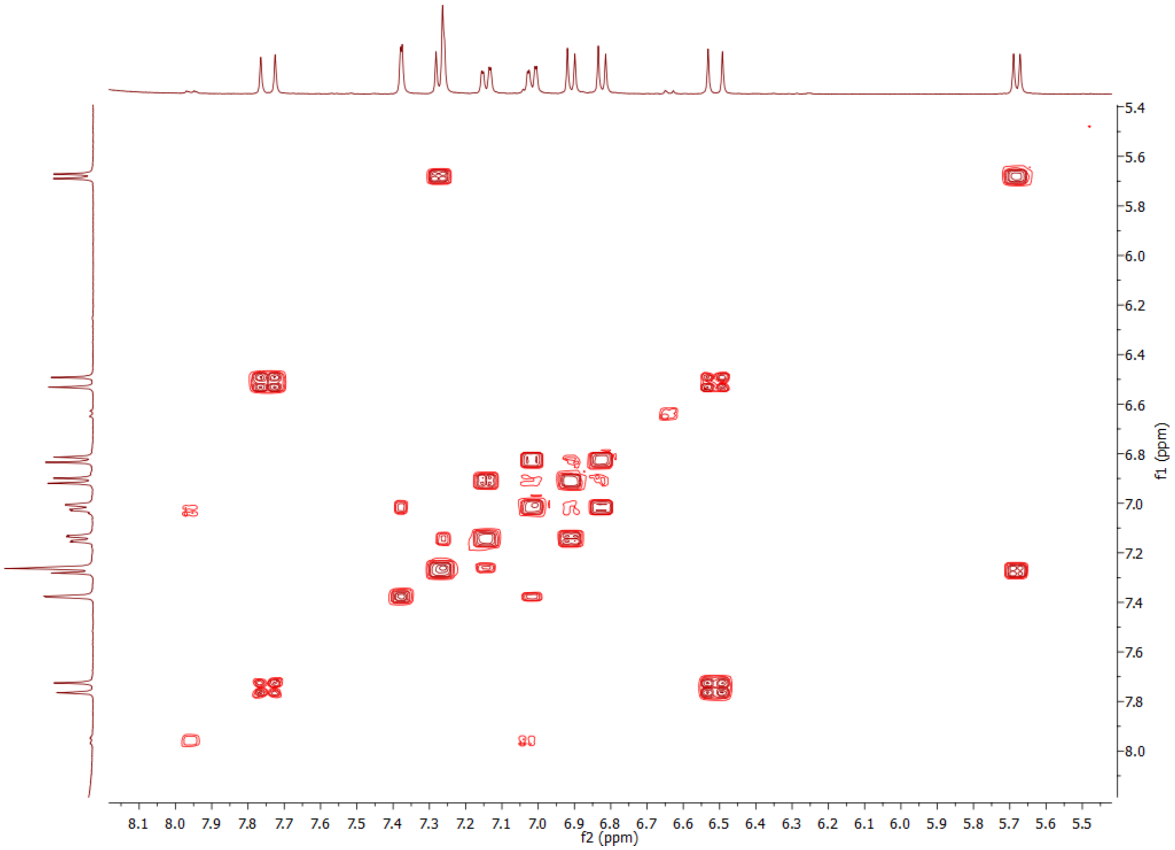

Figure S42. ^{1}H-^{1}H COSY (Acetone-*d*$_6$, 500 MHz) spectrum of compound **8**.
